# Enaminone Substituted Resorcin[4]arene—Sealing of an Upper-Rim with a Directional System of Hydrogen-Bonds

**DOI:** 10.3390/ijms21207494

**Published:** 2020-10-11

**Authors:** Anna Szafraniec, Marcin Grajda, Hanna Jędrzejewska, Agnieszka Szumna, Waldemar Iwanek

**Affiliations:** 1 Faculty of Chemistry, Adam Mickiewicz University in Poznań, Uniwersytetu Poznańskiego 8, 60-614 Poznań, Poland; szafraniec.anna0203@gmail.com; 2 Institute of Organic Chemistry, Polish Academy of Sciences, Kasprzaka 44/52, 01-224 Warsaw, Poland; marcin.grajda@icho.edu.pl (M.G.); hanna.jedrzejewska@icho.edu.pl (H.J.); agnieszka.szumna@icho.edu.pl (A.S.); 3 Faculty of Chemical Technology and Engineering, UTP University of Science and Technology, Seminaryjna 3, 85-326 Bydgoszcz, Poland

**Keywords:** resorcin[4]arene, crystallographic structure, conformation, DFT calculation, energy

## Abstract

The paper presents the synthesis of an enaminone resorcin[4]arene via a thermally activated *o*-quinomethide. The crystal structure indicates that in the solid state all enaminone units participate in a unidirectional seam of 12 intramolecular hydrogen bonds that are formed around the cavity. The molecule exhibits *C*_2_ symmetry, with two opposite-laying enaminone units directed inside the cavity (“*in*”), and the other two units outside the cavity (“*out*”). In the solution the enaminone resorcin[4]arene exists as a mixture of conformers with distribution controlled by temperature and solvent. The experimental data are compared with the results of theoretical calculations using DFT B3LYP/6-31G(d,p) and fast semi-empirical DFTB/GFN2-xTB method in various solvents.

## 1. Introduction

Resorcin[4]arenes are macrocyclic compounds with multidirectional possibilities of modifications of their structures [1]. “*Ortho*” positions and hydroxyl groups of the resorcin[4]arene platform can be functionalized regioselectively [2], resulting in a synthesis of a great number of derivatives with diversified molecular architectures, unique chirality (including inherent chirality), and tailor-made binding domains [3,4]. In a search for ways to control selectivity of binding particular attention is directed towards environment-dependent location of peripheral groups of resorcin[4]arenes. The concept of environmentally controlled position of peripheral groups was demonstrated by seminal works of Cram [5] and later substantially expanded by the Diederich group [6] and by our work [7]. The influence of various factors (temperature, solvent, pH) [8,9] on a vase-kite conformational transformation of cavitands and, thus, on their molecular shapes was exhaustively studied and subsequently applied in switching processes [10].

In this work, we present a new class of resorcin[4]arenes that can exist in “vase” or “kite” or mixed conformations, and these conformations are controlled by a hydrogen bonding motif in the upper rim. Moreover, dissymmetric character of a substituent attached to the central position of a resorcinol ring is able to create inherently chiral conformers [11]. Currently, several types of inherently chiral derivatives of resorcin[4]arenes are known in the literature. They involve derivatives that are covalently linked by modifications of hydroxyl groups [12,13,14] or by simultaneous modifications of the “ortho” position of the resorcin[4]arene and hydroxyl groups (via the Mannich reaction [15], cycloaddition reaction of o-quinomethide derivative of resorcin[4]arene with dienophiles [16,17], bridging with boron [18] or formation coumarin–type rings [19]. It has also been shown that inherent chirality can be induced in the non-covalent way by a system of hydrogen bonds and the barrier of racemization is relatively high (halflives the range of seconds to minutes) [20,21]. Importantly, such conformational chirality is a dynamic parameter that can be switchable [22].

**Scheme 1:**
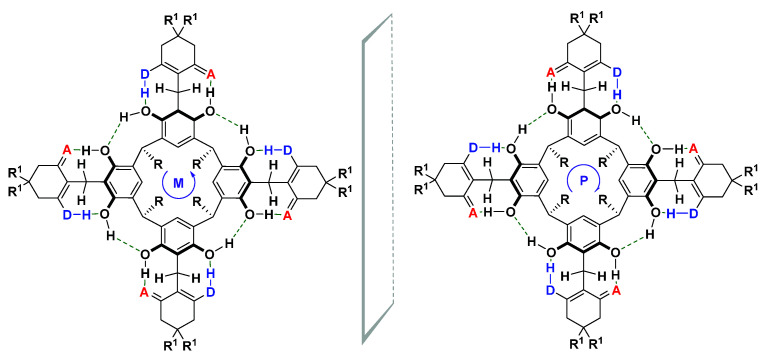
*M* and *P* enantiomers of inherently chiral enaminone derivatives of resorcin[4]arenes.

β-Enaminones have been widely used as key precursors in organic synthesis [23] as well as synthons for different important heterocycles [24], antibacterial [25], anticonvulsant, anti-inflammatory [26] and antitumor agents [27]. The enaminone group has a dual character: it can act as a hydrogen bond donor (DH) and an acceptor (A). Therefore, it can be used for the synthesis of conformationally inherently chiral derivatives even in the absence of any additional stereogenic centers via attachment to the “*ortho*” position of the resorcin[4]arene (*P* or *M* type, Scheme 1).

In this work we present, for the first time, the synthesis of enaminone resorcin[4]arenes. Their flexible structure, possibility to control conformational changes by a solvent and temperature along with unique chemical and biomedical properties of enaminone groups, are the appealing features for recognition and enzyme mimics.

## 2. Results and Discussion

**Scheme 2:**
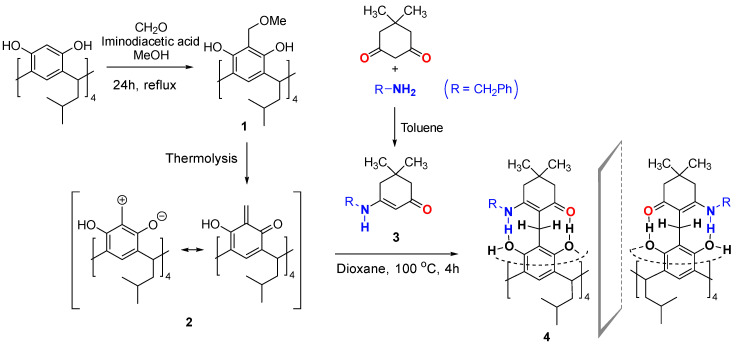
Synthesis of compound **4**.

For the synthesis of enaminone resorcin[4]arenes we have chosen the Michael reaction of enaminones with the *o*-quinomethide derivative of resorcin[4]arene **2** (Scheme 2). Quinomethides **2** that are known to be reactive intermediates that can participate in various reactions (e.g., Diels-Alder, Michael reaction) can be in-situ thermally generated from **1**. In order to obtain **4**, first enaminone **3** was synthesized by heating of benzylamine with dimedone for two hours in toluene with an azeotropic trap. Then, reaction of methoxy derivative of **1** with enaminone **3** in dioxane was performed and the product of the Michael reaction (**4**) was obtained in 85% yield.

**Figure 1:**
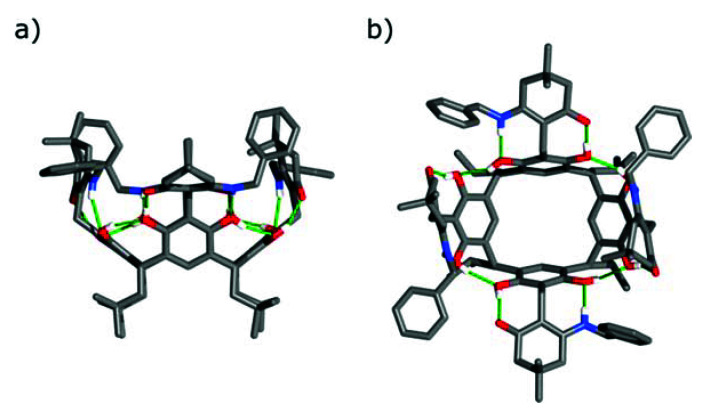
Crystallographic structure of **4**: (**a**) side view; (**b**) top view.

The formation of product **4** is not obvious in the light of previously reported reactions of other *o*-quinomethides with enaminones [28,29]. The previous examples indicate that, due to dipolar character of the dienophile and the *o*-quinomethide derivative (the resonance structures), a cycloaddition reaction should dominate, and the product should be cyclic. However, the reaction of **2** with **3** does not proceed by cycloaddition, but via the Michael reaction. The formation of **4** may be favoured by stabilization by a system of intramolecular hydrogen bonds, which was indeed observed in the X-ray structure of **4** (Figure 1). Single crystals of **4** suitable for crystallographic studies were obtained by crystallization from methanol. In the crystal, the molecule of **4** exhibits C_2_ symmetry, with two opposite-laying enaminone units directed inside the cavity (“*in*”), and the other two units outside the cavity (“*out*”). The in/out position is defined by the torsion angles around C_ar_-CH_2_ bond. Interestingly, all enaminone units that participate in a seam of 12 intramolecular hydrogen bonds exhibit the same “directionality”, i.e., the benzylamine parts are always turned in the same direction. Different in/out positions do not affect either the formation or the directionality of this seam. One can deduce that that turning one enaminone unit in the opposite direction, the would reduce the maximum number of hydrogen bonds by two and provide unfavourable DH···DH and A···A interactions. Thus, even though the conformation in terms of *in*/*out* position varies, the directionality and thus inherent chirality remains the same for all enaminone units.

**Figure 2:**
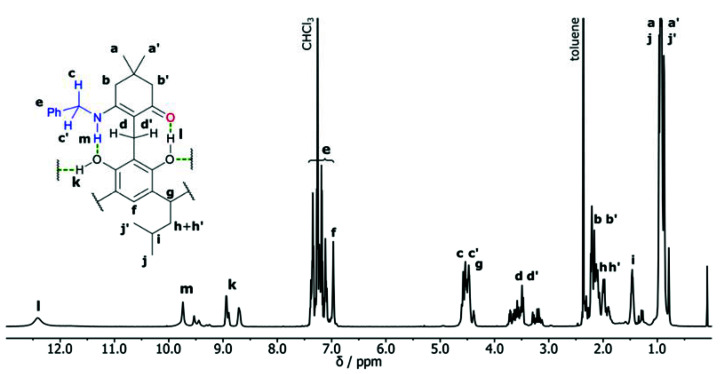
^1^H NMR spectrum of **4** (CDCl_3_, 298 K, 600 MHz).

Structural and conformational analysis of compound **4** was performed with NMR method in CDCl_3_ and DMSO-d_6_ solvents. They are typical solvents for NMR analysis and they have substantially different polarity and proton-donor-acceptor properties. Therefore, it was expected that they will allow us to study product **4** in two different states: the form with intramolecular hydrogen bonds (in chloroform) and without hydrogen bonds (in DMSO). The ^1^H NMR spectrum of **4** is complicated (Figure 2) suggesting that at 298 K enaminone resorcin[4]arenes is in the form of a mixture of several conformers. This is particularly visible in the region of signals of diastereotopic methylene protons CH_2_ (d,d′) and signals of protons involved in the formation of hydrogen bonds. Diastereotopic methylene protons (d,d′) are observed as multiplets spread over a relatively wide range of chemical shift. This may be caused by their different *in*/*out* position, that are characterized by substantially different magnetic environments (CH_2_ “*in*” shifted towards the lower magnetic field, while CH_2_ “*out*” shifted towards higher magnetic field).

**Figure 3:**
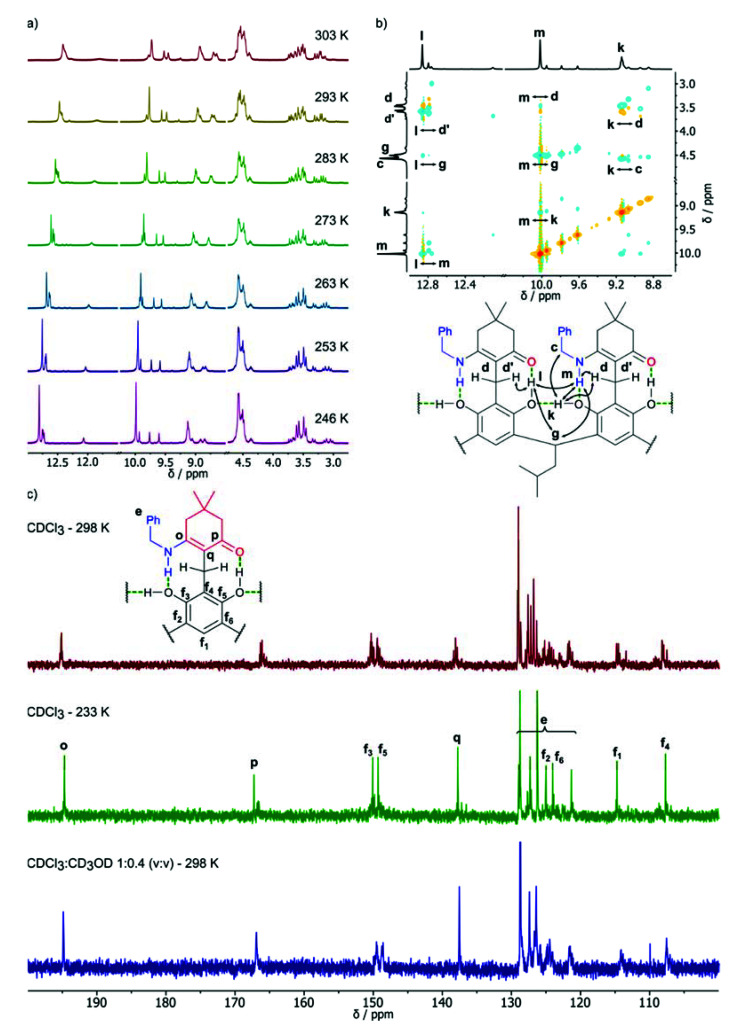
VT NMR spectra of **4**: (**a**) ^1^H NMR (400 MHz, CDCl_3_) temperature range 246–313 K; (**b**) partial ROESY spectrum (600 MHz, CDCl_3_, 233 K) (**c**) ^13^C NMR spectra at different temperatures and solvents (all at 600 MHz).

ROESY spectrum indicates that all diastereotopic methylene protons are involved in a chemical exchange., confirming that this complex pattern in the spectrum reflects conformational variations. Protons of the hydroxyl groups OH (l,k) and protons of the benzylamine groups PhCH_2_NH (m) that participate in the formation of intramolecular hydrogen bonds are found in the ^1^H NMR spectrum in the range of 8.5–12.5 ppm. ROESY spectrum at lower temperature (233 K, Figure 3b) allowed us to identify the proximity of the hydroxyl group OH (k) to the amino group PhCH_2_NH(m), the methylene group (d) and the benzyl group (c). The other hydroxyl group, OH (l), is located close to the methylene group (d′), methine CH (g) and the amino group PhCH_2_NH(m). The amino group PhCH_2_NH (m) is in the proximity to one of the diastereotopic protons of the methylene group (d) and the methine proton CH (g). These intramolecular contacts are in agreement with the postulated structure of **4**.

VT NMR spectra support the hypothesis that at room temperature compound **4** exists as a mixture of conformers (Figure 3a). Upon lowering the temperature in the range of 246-313 K the shape of the signal changes and one of the sets of signals becomes dominant. This is in agreement with the Boltzman distribution and with a general rule that upon lowering of the temperature the lower energy conformers become more abundant. The set of signals that are amplified at low temperature corresponds to a conformer that having *C*_4_ symmetry (with all groups positioned either *in* or *out*). For example, in the ^1^H NMR spectrum, the signals of diastereotopic methylene protons CH_2_ (d,d′) become a doublet of doublets with a spin-spin coupling constant *J* = 14.80 Hz. Substantial simplification upon temperature lowering is also observed in the ^13^C NMR spectrum (Figure 3c). At 298 K the ^13^C NMR spectrum of **4** contains multiplied signals, for example, for carbonyl groups C = O (p), enamine groups N-C=C (o), and the aromatic carbons (f_3_, f_5_). Lowering the temperature (233 K, CHCl_3_) leads to an appearance on a one dominating set of signals, which is in agreement with *C*_4_ symmetry. The ^13^C NMR spectrum of compound **4** in a mixture of CDCl_3_/MeOH (1: 0.4, v/v) at 298 K shows an increase in the population of one of the conformers. Importantly, non-equivalency of f_3_ and f_5_ signals in ^13^C NMR spectrum is the characteristic feature of inherently chiral C_4_-symmetric structures. Here, the difference in chemical shifts of aromatic carbons (f_3_, f_5_) at 233 K is 0.78 ppm. This value is comparable with the previously reported values for cyclochiral tetramethoxy-resorcin[4]arenes [30,31]. All these NMR results are in agreement with the temperature-induced changes in the conformer distribution and predominant presence of the C_4_-symmetric conformer at low temperatures.

**Figure 4:**
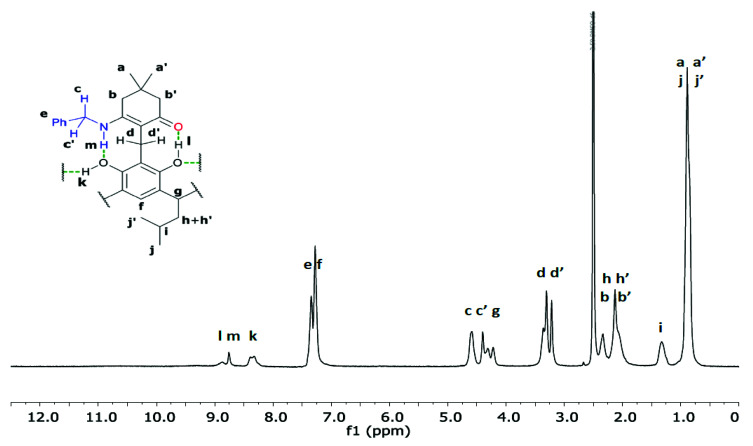
^1^H NMR spectrum of **4** in DMSO-d_6_ at 303 K.

The spectrum of **4** in DMSO features broad signals (Figure 4). The differentiation of signals of diastereotopic protons of the methylene group CH_2_ (d,d′) is not observed. Importantly, virtually no changes are observed in the temperature range of 303–343 K (Figure S1), indicating that in DMSO. compound **4** is highly conformationally labile.

**Figure 5:**
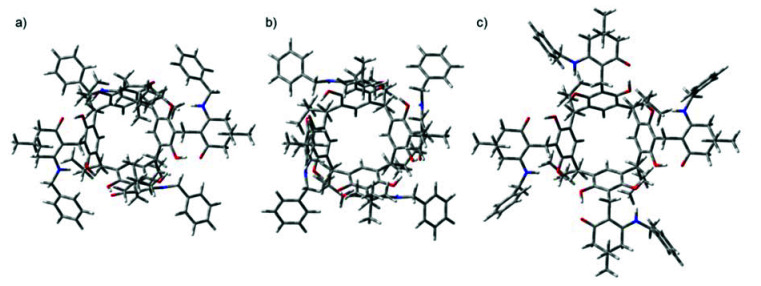
Conformations of **4** optimized by DFT/B3LYP: (**a**) *in-out* (*C*_2_), (**b**) *all-in* (*C*_4_), (**c**) *all-out* (*C*_4_).

To compare the relative stability of conformers, theoretical calculations were performed within the density functional theory (DFT) approach using the Gaussian 09 program suite [32] and DFTB/GFN2-xTB [33]. Three initial conformers were constructed: (1) *in-out* (the same as the solid-state structure), (2) *all-in* and (3) *all-out* obtained by modification of torsion angles of the solid-state structure. The structures were optimized using different methods in chloroform and DMSO (Figure 5 and Table 1).

Theoretical calculations using two different methods show different results. The DFT/B3LYP optimization predicts the *all-in* conformation to be the most stable in chloroform and DMSO. The DFTB/GFN2-xTB method predicts *in-out* and *all-out* conformations to be distributed at 1:3 ratio in chloroform, while the *all-out* conformation to be exclusively present in DMSO. Comparing these results with the experimental NMR spectra, it seems that DFTB/GFN2 calculations give the results that are consistent with the experimental findings. In the ROESY spectrum, there are no signals indicating close proximity of the hydroxyl group OH (l) of the enaminone unit with the benzyl group PhCH_2_ (c), that should be inevitably present in the case of *all-in* conformation. Therefore, of the ROESY spectrum suggests that the dominant conformation at 233 K in CHCl_3_ is *all*-*out*, in agreement with DFTB/GFN2 calculations. For the *all*-*in* conformer, we should observe such an interaction, because quantum-mechanical calculations indicate their proximity, respectively: 3.10 Å (DFTB/GFN2) and 3.28 Å (DFT/B3LYP). Moreover, the calculations show a smaller distance between the proton of the amino group PhCH_2_NH (m) and the methine proton CH (g) for the *all*-*out* conformer than for the *all*-*in* conformer, respectively: for the all_-_*out* conformer (3.94 Å—DFT/B3LYP; 3.74 Å—DFTB/GFN2) and *all*-*in* conformer (4.21 Å—DFT/B3LYP; 4.16 Å—DFTB/GFN2).

**Figure 6:**
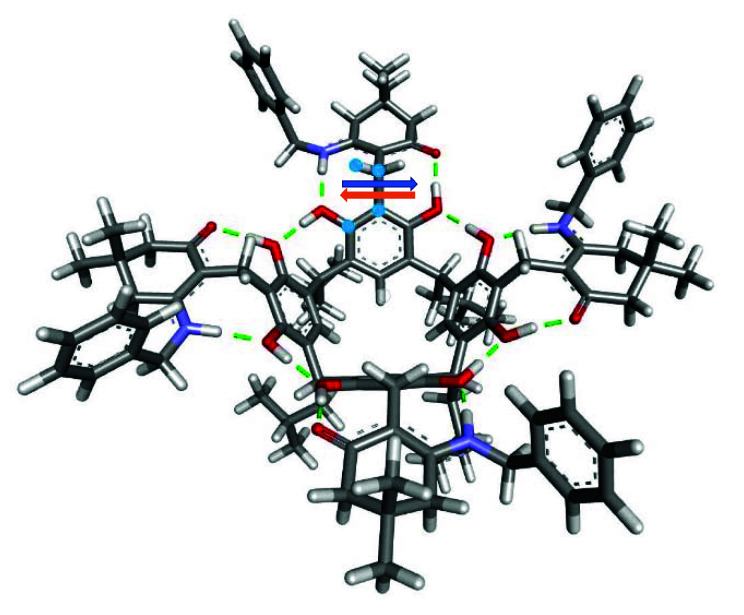
The structure of *all*-*out* conformer of derivative 4 with the direction of dihedral angle (atoms marked in blue) marked with an arrow: blue arrow—increasing the value of the dihedral angle; orange arrow—decreasing the value of the dihedral angle.

**Figure 7:**
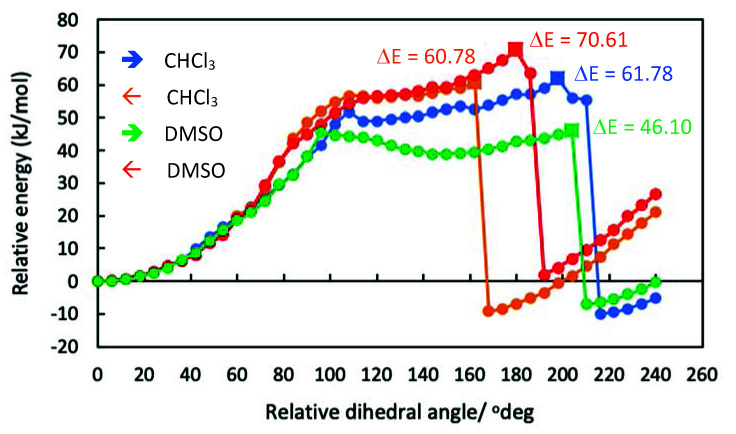
The relative total energies (ΔE) for the conformer *all*-*out* during dihedral angle changes (blue—the dihedral angle increases, CHCl_3_; orange—the dihedral angle decreases, CHCl_3_; green—the dihedral angle increases, DMSO; red—the dihedral angle decreases, DMSO). The maxima on each curve are marked with squares together with their values.

To estimate the activation energy of rotation of the enaminone unit with respect to the macrocyclic base, the changes of energy were probed while modifying the dihedral angle (atoms marked in blue, 0 ÷ 240° by 6° deg in two directions (blue and orange arrows) (Figure 6) in the *all-out* conformer. The calculations were performed in CHCl_3_ and DMSO using a GFN2-xTB semiempirical DFTB method. Geometry optimization was performed after each change of the dihedral angle and the results are presented in Figure 7.

The change of the relative total energy of the *all-out* conformer depends on the direction and the magnitude of changes of the dihedral angle. Particularly large differences are found in DMSO. The relative total energies of the transition structures reach higher values upon rotation in one direction, red curve, E_max_ = 70.61 kJ/mol than upon rotation in the opposite direction (green curve, ΔE_max_ = 46.10 kJ/mol). This may reflect the fact that upon rotation in different directions in different groups are located close to the cavity: either aminobenzyl group that can better adapt to the cavity (changing the position of the enaminone unit from *out* to *in* or the rigid dimedone part of the that possibly increases the energy of the transition state. It should be emphasized that the transitional structures related to the change of the position of the enamino substituent are realized at different dihedral angles. For a lower energy transition structure, this angle is 224.6 °deg; for the transition structure with higher energy, it is −159.4 °deg. Taking into account the proton-acceptor properties of DMSO, we can expect the weakening of intramolecular hydrogen bonds in the conformers *all*-*out* in DMSO. These interactions significantly reduce the activation energy of the *out*-*in* position, switching of enaminone units in derivative 4. These data are consistent with experimental observations showing the high conformational lability of derivative 4 in DMSO. Are compatible as well with the theoretical calculations by DFTB/GNF2, indicating that the most stable conformer of derivative 4 in DMSO is the *all*-*out* conformer.

**Figure 8:**
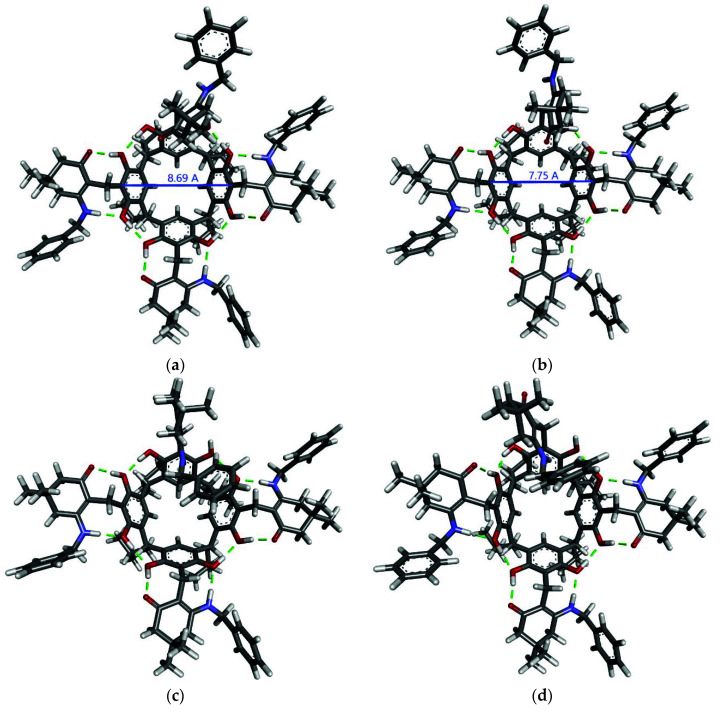
The transition structures showing changes in the position *out-in* of the enaminone unit of the *all*-*out* conformer. (**a**) ΔE = 70.61 kJ/mol. (**b**) ΔE = 60.78 kJ/mol. (**c**) ΔE = 46.10 kJ/mol. (**d**) ΔE = 61.78 kJ/mol.

In CHCl_3_, the situation is different. Regardless of the direction of changes in the magnitude of the dihedral angle, we observe very similar relative maximum energies of the transition structures. In the case of an increase in the value of the dihedral angle (blue curve), the relative maximum energy of the transition state is ΔE_max_ = 61.78 kJ/mol; if the change is in the opposite direction, ΔE_max_ = 60.78 kJ/mol. On the other hand, the dihedral angles at which these maxima occur change. In the first case, the angle is −147.7 °deg, and in the second case, it is 218.6 °deg. There is a clear increase in the activation energy of changing the position of the enaminone substituent in relation to DMSO ((ΔΔE_a_ = 14.68 kJ/mol). This is probably related to the stiffness of the *all*-*out* conformer in CHCl_3_ by enhancing intramolecular hydrogen bonds. This is indicated by the distances between the opposite resorcinol rings in the *all*-*out* transition structures, calculated by DFTB/GFN2 in DMSO and CHCl_3_. They are respectively: 8.69 Å in DMSO and 7.75 Å in CHCl_3_ and are marked in Figure 8. This causes an increase in the energy of intermolecular interactions during the rotation of the enaminone unit from *out* to *in*.

On all curves, there are characteristic “jumps” in the relative total energy that are associated with the recovery of the hydrogen bonds by rotating the enaminone unit. In Figure 7, the characteristic points that represent the maximums energy of the *all*-*out* conformer of compound 4 with respect to changes in the dihedral angle are marked with squares for the distinction. These transition structures showing changes in the position of the enaminone unit of the *all*-*out* conformer from the *out* to the *in* position are shown in Figure 8.

## 3. Materials and Methods

The NMR spectra were achieved using a Avance 400 and 600 MHz ultra-shield spectrometer (Bruker, Karlsruhe, Germany). The mass spectra were recorded by electrospray ionisation (ESI) coupled with a TOF analyser (Bruker, Karlsruhe, Germany). The reaction was completed using a Monowave 50 reactor (Anton Paar, Graz, Austria). Reagents and solvents were obtained from Sigma-Aldrich, Fluka, and Merck and were used without purification. Methoxy derivative **1** was synthesized according to the literature [34].

The geometry of compound **4** was optimized with the B3LYP functional, employing the 6-31G (d, p) basis set using the Gaussian 09 program suite. Solvent effects were considered within the SCRF theory using the polarized continuum model (PCM) approach to model the interaction with the solvent. Semiempirical DFTB/GFN2-xTB method was also applied for the calculations, using the S. Grimme software [35]. Solvent effects were considered within generalized born (GB) with solvent accessible surface area (SASA) termed GBSA. The calculations of the optimized conformer structures were completed using an Econv/Eh of 1 × 10^−8^, a Gconv/Eh·α^−1^ of 5 × 10^−^^5^, and an accuracy of 0.01. The changes in the relative total energy (kJ/mol) of the conformer *all*-*out* of derivative 4 with respect to the relative dihedral angle changes were calculated using an Econv/Eh of 1 × 10^−^^7^, a Gconv/Eh·α^−1^ of 2 × 10^−4^, and an accuracy of 0.05.

Diffraction data for **4** were collected at 130(1) K on Rigaku four-circle SuperNova diffractometer with Atlas detector and graphite-monochromated Cu Kα radiation (λ = 1.54184 Å). The data were corrected for Lorentz-polarization as well as for absorption effects. Using Olex2 [36], the structure was solved with the SHELXT [37] structure solution program using Intrinsic Phasing and refined with the SHELXL [38] refinement package using Least Squares minimisation. All non-hydrogen atoms were refined anisotropically, hydrogen atoms were placed in idealized positions and refined as a ‘riding model’ with isotropic displacement parameters set at 1.2 (1.5 for methyl groups) times Ueq of appropriate carrier atoms.

Compound (**4**): The methoxy derivative of resorcin[4]arene (1) (0.112 mmol, 100 mg) and 3- (benzylamino)-5,5-dimethylcyclohex-2-en-1-one (2) (0.448 mmol, 103 mg, 4 equivalents) were placed in the reaction vessel and 6 mL of dioxane was added. The mixture was heated at 100 °C for 4 h. After cooling to room temperature, the dioxane was evaporated from the reaction mixture and the remaining solid was crystallized from methanol. Compound **4** was obtained as a white solid (160 mg, 85% yield), m.p. > 300 °C. ^1^H NMR (400 MHz, CDCl_3_, T = 233 K) δ = 12.80 (s, 4H, OH (l)), 9.95 (s, 4H, NH (m)), 9.08 (s, 4H, OH (n)), 7.40–6.90 (m, 24H, ArH), 4.51 (m, 8H, CH_2_ (c,c′)), 4.44 (t, 4H, CH (h), 3.51 (d, *J* = 14.80 Hz, 4H, CH_2_ (d′), 3.40 (d, *J* = 14.80 Hz, CH_2_ (d)), 2.25–1.80 (m, 16H (b,b′)), 1.88 (m, 8H, CH_2_ (I,I′)), 1.38 (m, 8H, CH (j)), 0.99–0.69 (m, 48H, CH_3_ (a,a′), CH_3_ (k,k′); ^13^C NMR (100 MHz, CDCl_3_, T = 233 K) δ = 195.69, 167.26, 150.08, 149.30, 138.03, 137.75, 129.09, 128.76, 128.29, 127.30, 126.27, 125.31, 124.98, 121.31, 114.72, 107.69, 48.42, 46.51, 42.31, 38.83, 31.70, 31.57, 30.34, 26.16, 25.82, 23.38, 22.71, 21.69, 19.31 ppm; HRMS ESI *m*/*z* for C_108_H_132_N_4_O_12_ [M+H]^+^ calcd 1677.9915, found 1677.9934.

Crystal Data for **4**: C_232_H_323_N_8_O_40_; M_r_ = 3863.96, triclinic, space group P1 (no. 1), a = 16.5701(3) Å, b = 17.4959(4) Å, c = 20.7166(4) Å, α = 83.190(2)°, β = 78.017(2)°, γ = 76.256(2)°, V = 5691.7(2) Å^3^, Z = 1, T = 130(1) K, μ(Cu Kα) = 0.608 mm^−1^, d_x_ = 1.128 g/cm^3^, 80,685 reflections measured (4.372° ≤ 2Θ ≤ 151.73°), 32,985 unique (Rint = 0.056, Rsigma = 0.043) which were used in all calculations. Final R[I >2σ(I)] = 8.03%, wR2[I > 2σ(I)] = 20.36%, R[all refl.] = 8.71%, wR2[all refl.] = 20.36%, S = 0.907, Δρmax/Δρmin = 0.61/−0.37 e·Å^−3^.

CCDC 1,969,740 contains the supplementary crystallographic data for this paper. The data can be obtained free of charge from The Cambridge Crystallographic Data Centre via www.ccdc.cam.ac.uk/structures.

## 4. Conclusions

We presented an efficient synthesis of new enaminone resorcin[4]arene (**4**). A crystal structure of **4** shows that in the solid-state it adopts the *in-out* conformation and it sealed with 12 undirectional hydrogen bonds. In the solution, the structure is highly dynamic and at room temperature in chloroform **4** is present as a mixture of conformers with peripheral groups positioned either “*in*” and “*out*”. Theoretical calculations of the relative stability of conformers (DFTB/GFN2 method) indicate that *in-out* and *all-out* conformers dominate at different ratios depending on the temperature in chloroform. In DMSO labile *all*-*out* conformer is dominant. The activation energy of the change of the position of the enaminone unit from the *out* to the *in* position in the *all*-*out* conformer in DMSO calculated with this method is ΔE_a_ = 46.10 kJ/mol. In CHCl_3_ it is higher and amounts to ΔE_a_ = 60.78 kJ/mol. Conformational dynamics of the enaminone derivatives of resorcin[4]arene, depending on the solvent and temperature, may be interesting from the point of view of the role of conformational alignment in molecular recognition.

## Figures and Tables

**Table 1:** Relative energies of conformers of **4** calculated by DFT/B3LYP and DFTB/GFN2-xTB and their theoretical populations at 298 K.

	Chloroform		DMSO	
conformation (symmetry)	DFT/B3LYP ∆E/kJ·mol^−1^ (population %)	DFTB/GFN2-xTB ∆E/kJ·mol^−1^ (population%)	DFT/B3LYP ∆E/kJ·mol^−1^ (population %)	DFTB/GFN2-xTB ∆E/kJ·mol^−1^ (population%)
*all-in* (C_4_)	0 (85%)	18.86 (<1%)	0 (83%)	16.08 (<1%)
*in-out (C_2_)*	5.55 (9%)	0.68 (43%)	4.71 (13%)	18.47 (<1%)
*all-out* (C_4_)	6.51 (6%)	0 (57%)	7.17 (4%)	0 (100%)

## Supplementary Materials

The following are available online at https://www.mdpi.com/1422-0067/21/20/7494/s1.
