# Supplementary material for: Enaminone Substituted Resorcin[4]arene—Sealing of an Upper-Rim with a Directional System of Hydrogen-Bonds
Source: Int J Mol Sci. 2020 Oct 11;21(20):7494. doi: 10.3390/ijms21207494 (PMC7589316; doi:10.3390/ijms21207494)
Supplement: Supplementary file 1 [file ijms-21-07494-s001.pdf]

Enaminone substituted resorcin[4]arene – a scaffold with hydrogen-bond based motif for upper-rim sealing

Anna Szafraniec,[a] Marcin Grajda,[b] Hanna Jędrzejewska,[b] Agnieszka Szumna,[b] Waldemar Iwanek [c*]

ELECTRONIC SUPLEMENTARY INFORMATION

Table of contents:

1. Figure S1. Variable temperature ^{1}H NMR spectra of compound **4** in DMSO-d_6 (temperature range 303-343 K).
2. Figure 2. ^{1}H and ^{13}C NMR spectrum of compound **2** in $CDCl_3$ at 298 K
3. Figure 3. ^{1}H and ^{13}C NMR spectrum of compound **4** in $CDCl_3$ at 298 K
4. Figure 4. ^{1}H and ^{13}C NMR spectrum of compound **4** in $CDCl_3$ at 233 K
5. Figure 5 Variable temperature of ^{1}H NMR spectra of compound **4** (in $CDCl_3$, temperature range 246-313 K).
6. Figure 6. ^{1}H,^{1}H COSY spectrum of compound **4** in $CDCl_3$ at 233 K
7. Figure 7. ROESY spectrum of compound **4** in $CDCl_3$ at 233 K
8. Figure 8. MS spectrum of compound **4**
9. DFT/B3LYP calculations
10. DFTB/GFN2-xTB calculations

Figure S1. Variable temperature ^{1}H NMR spectra of compound **4** (DMSO-d_6, temperature range 303-343 K).

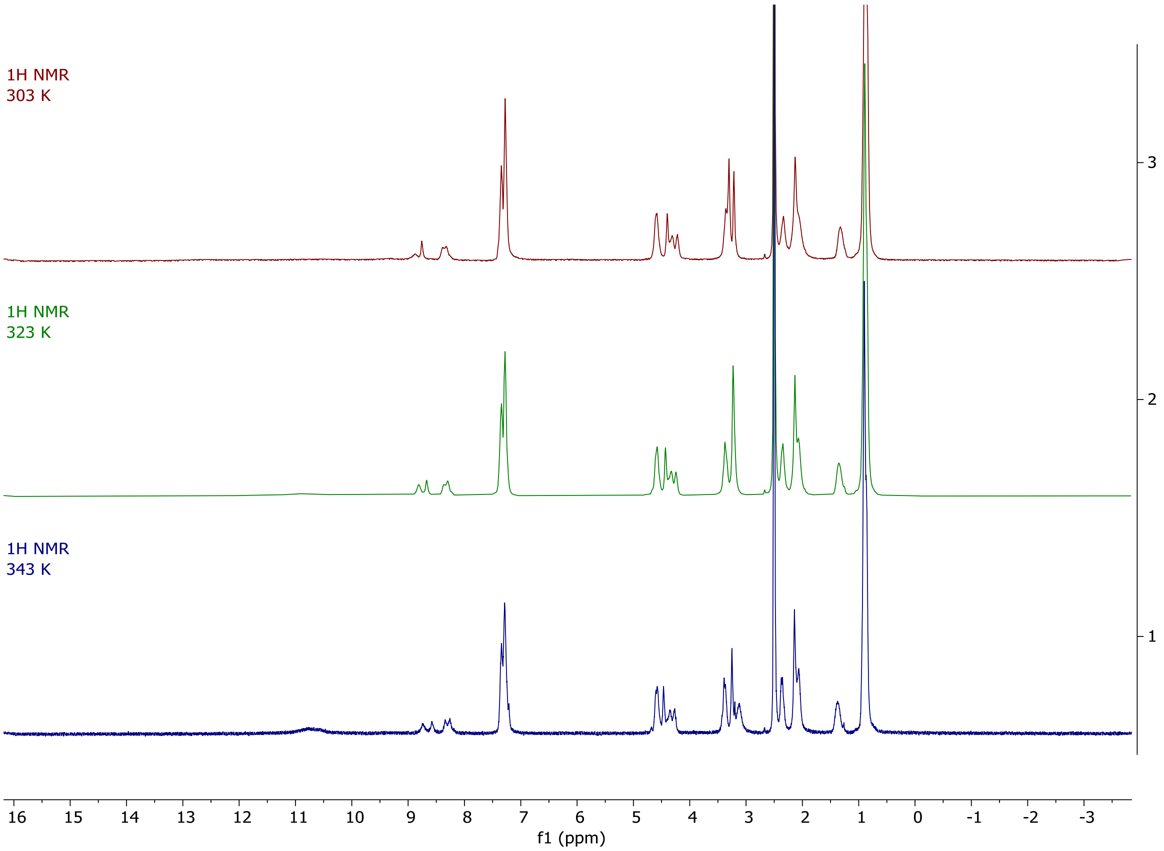

Figure 2. ^{1}H and ^{13}C NMR spectrum of compound **2** in $CDCl_3$ at 298 K

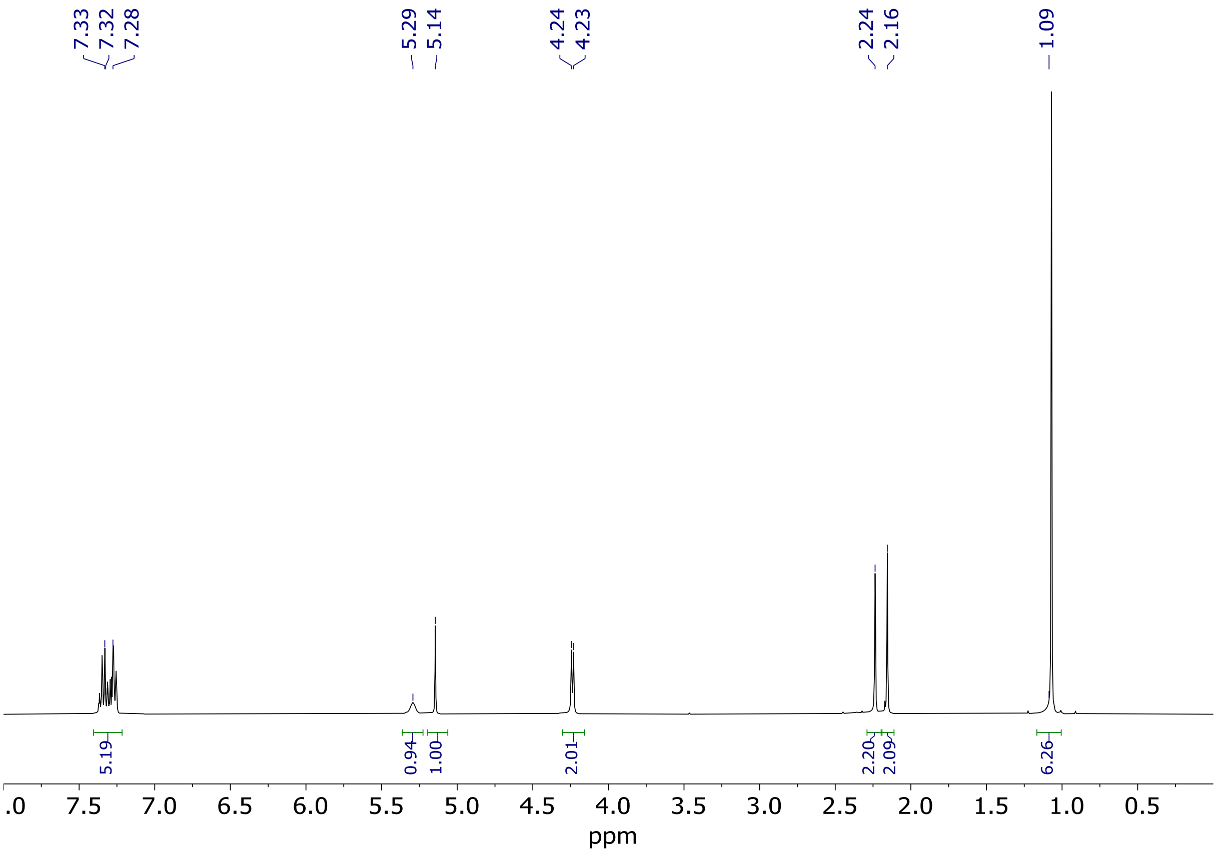

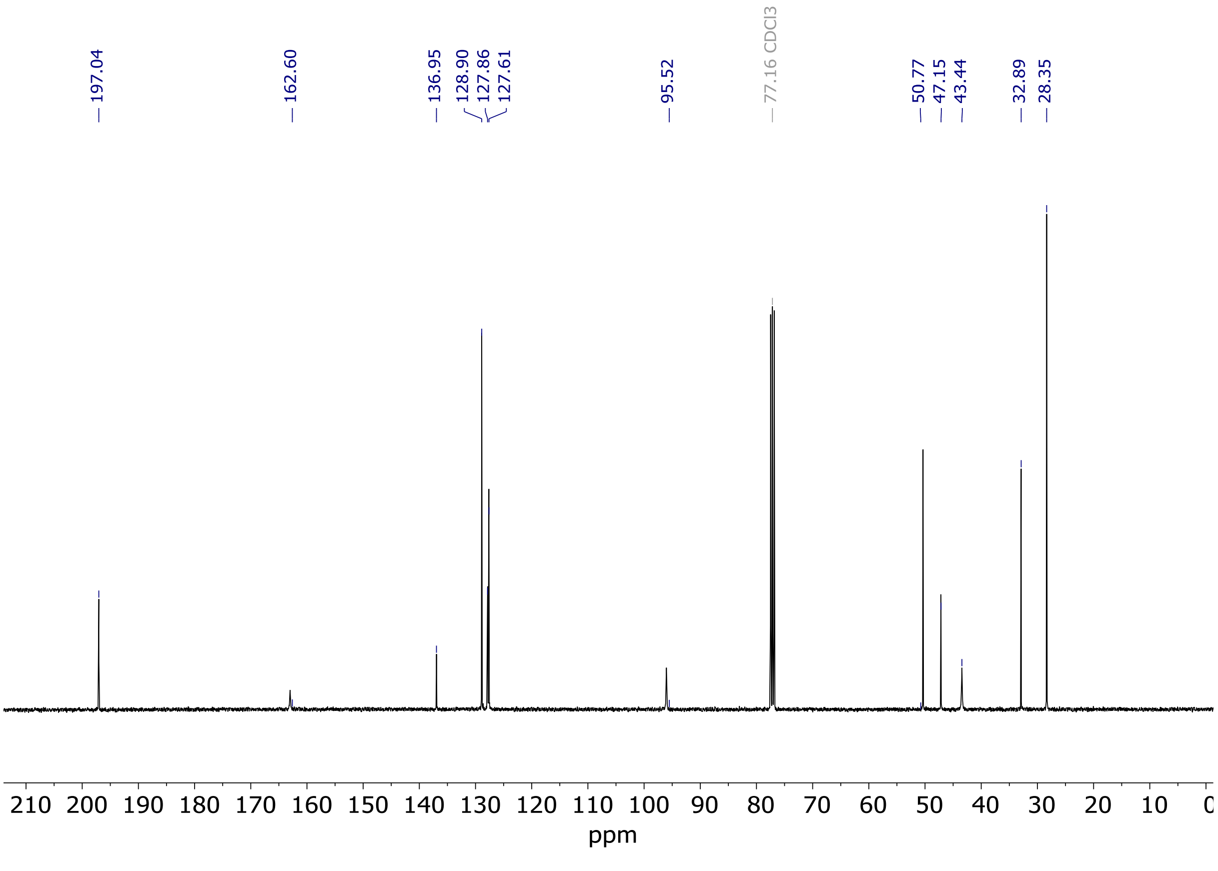

Figure 3. ^{1}H and ^{13}C NMR spectrum of compound **4** in $CDCl_3$ at 298 K

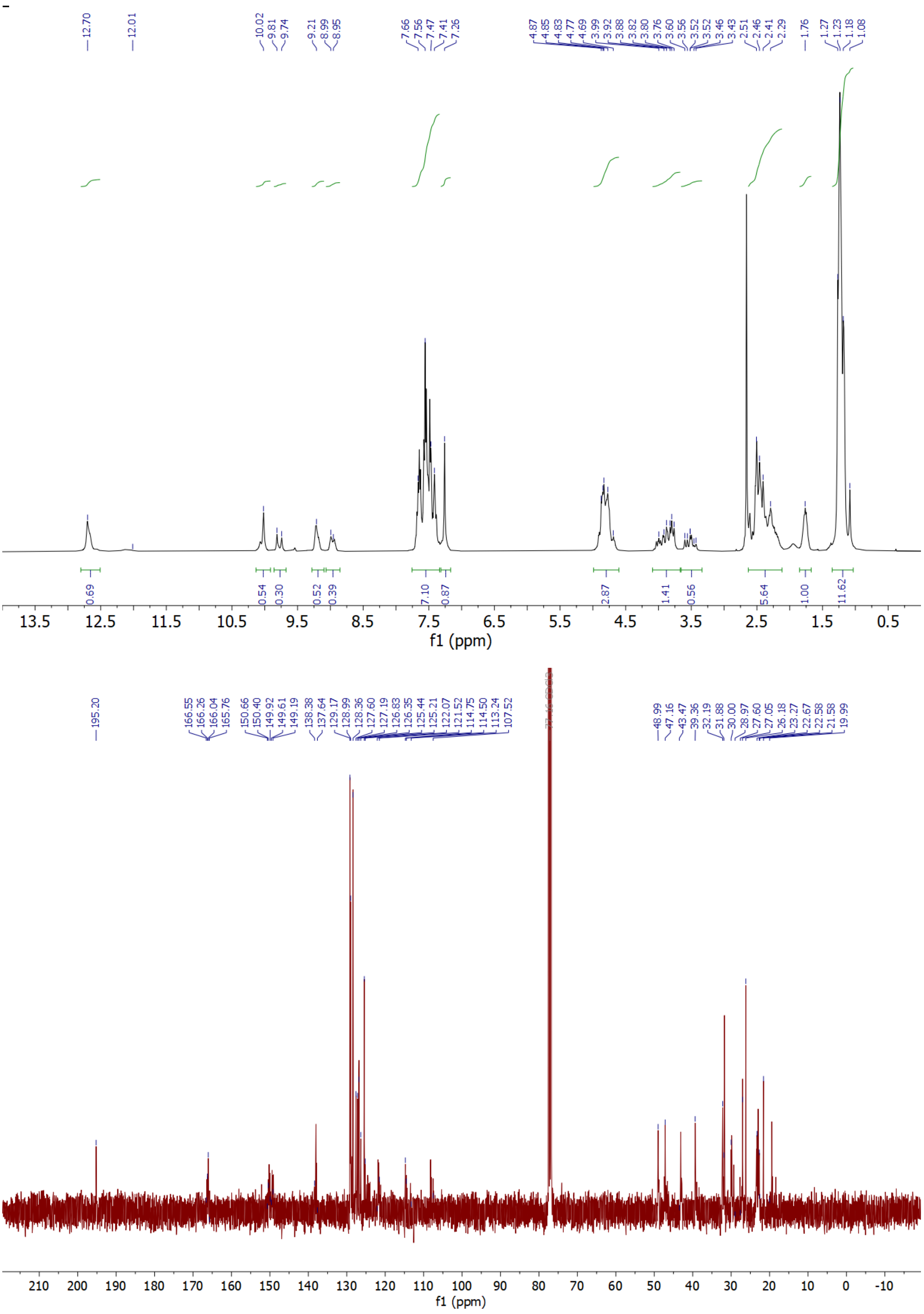

Figure 4. ^{1}H and ^{13}C NMR spectrum of compound **4** in $CDCl_3$ at 233 K

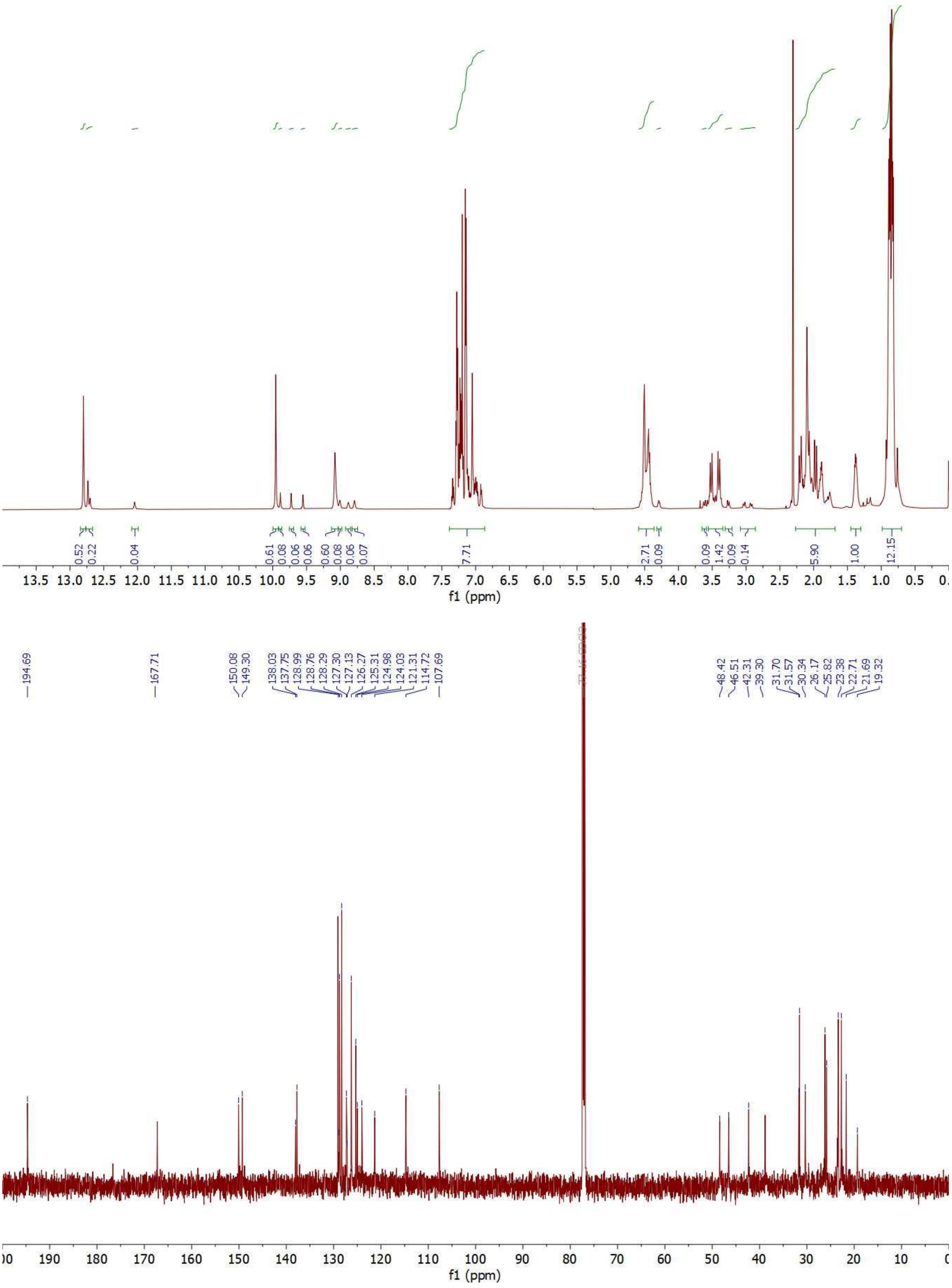

Figure 5. Variable temperature ^{1}H NMR spectra of compound **4** (in $CDCl_3$, temperature range 246-313 K).

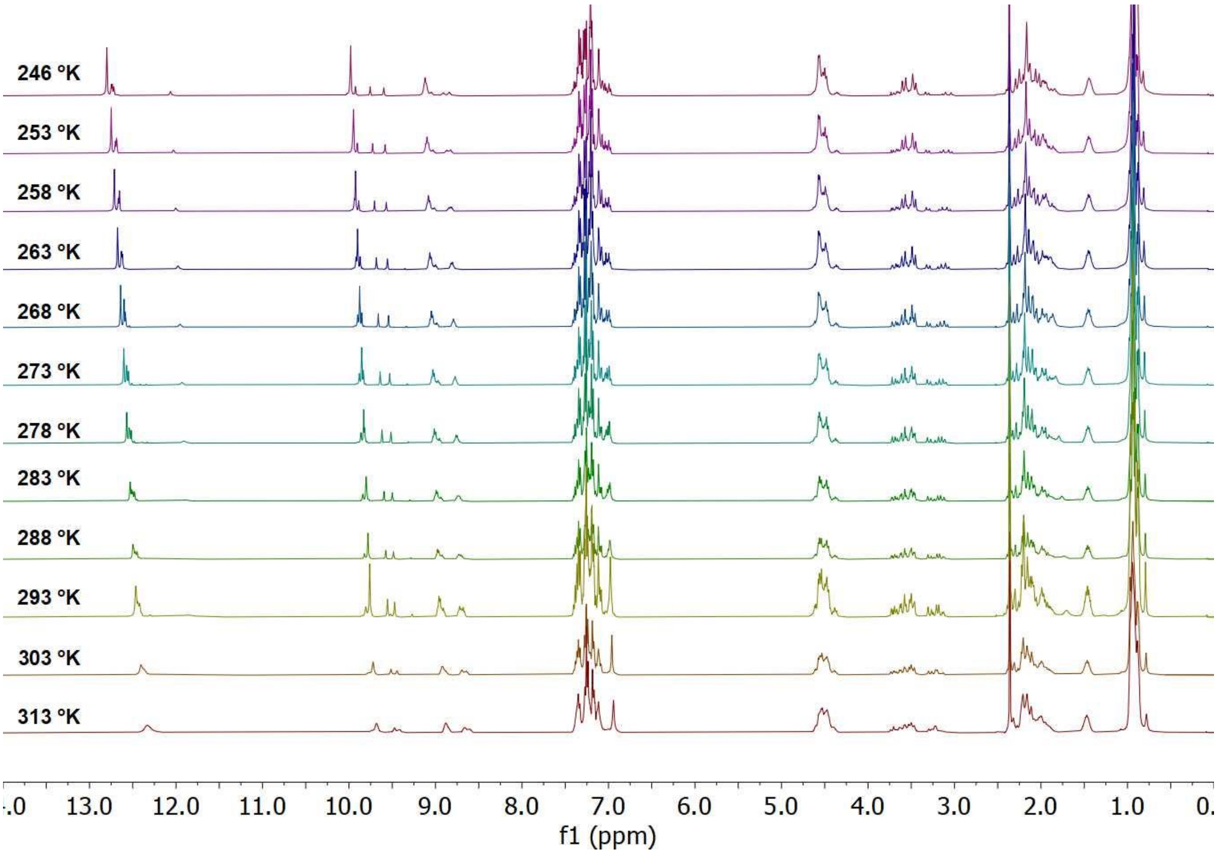

Figure 6. $^1H,^1H$ COSY spectrum of compound **4** in $CDCl_3$ at 233 K

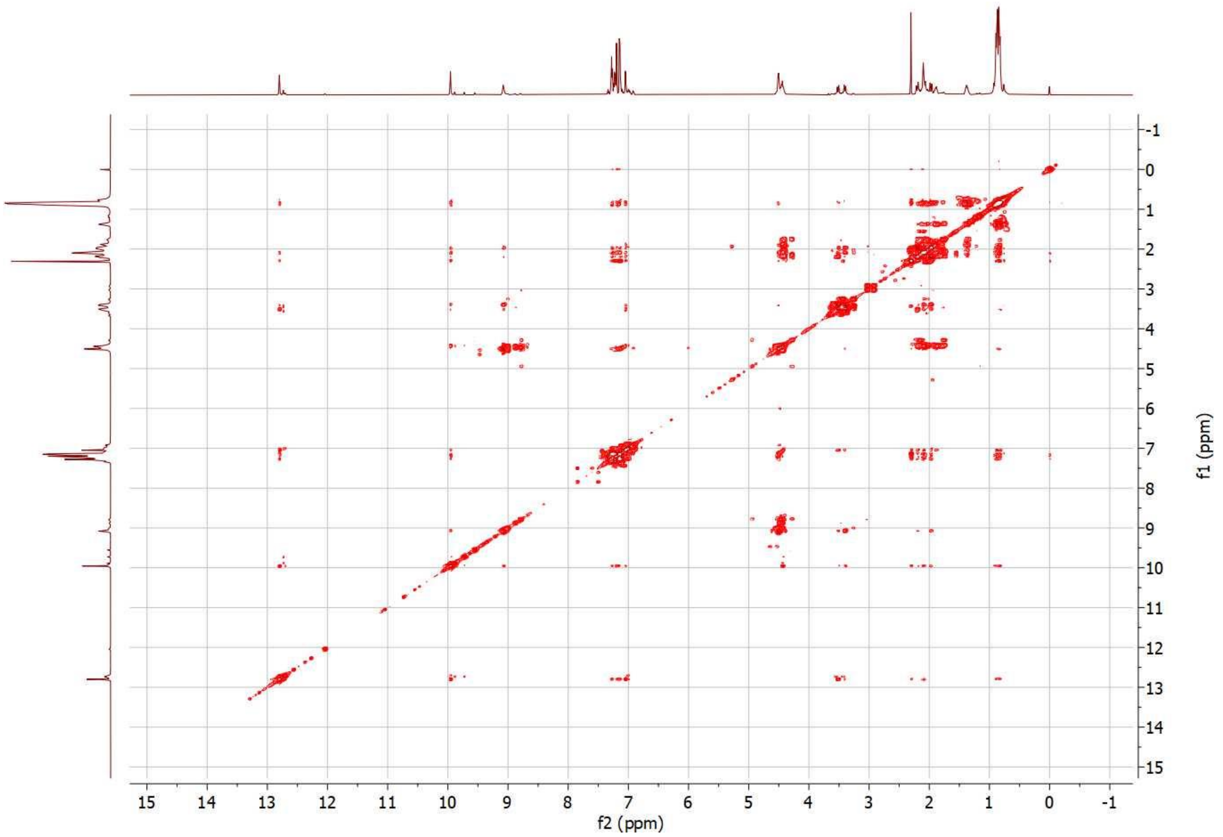

Figure 7. ROESY spectrum of compound **4** in $CDCl_3$ at 233 K

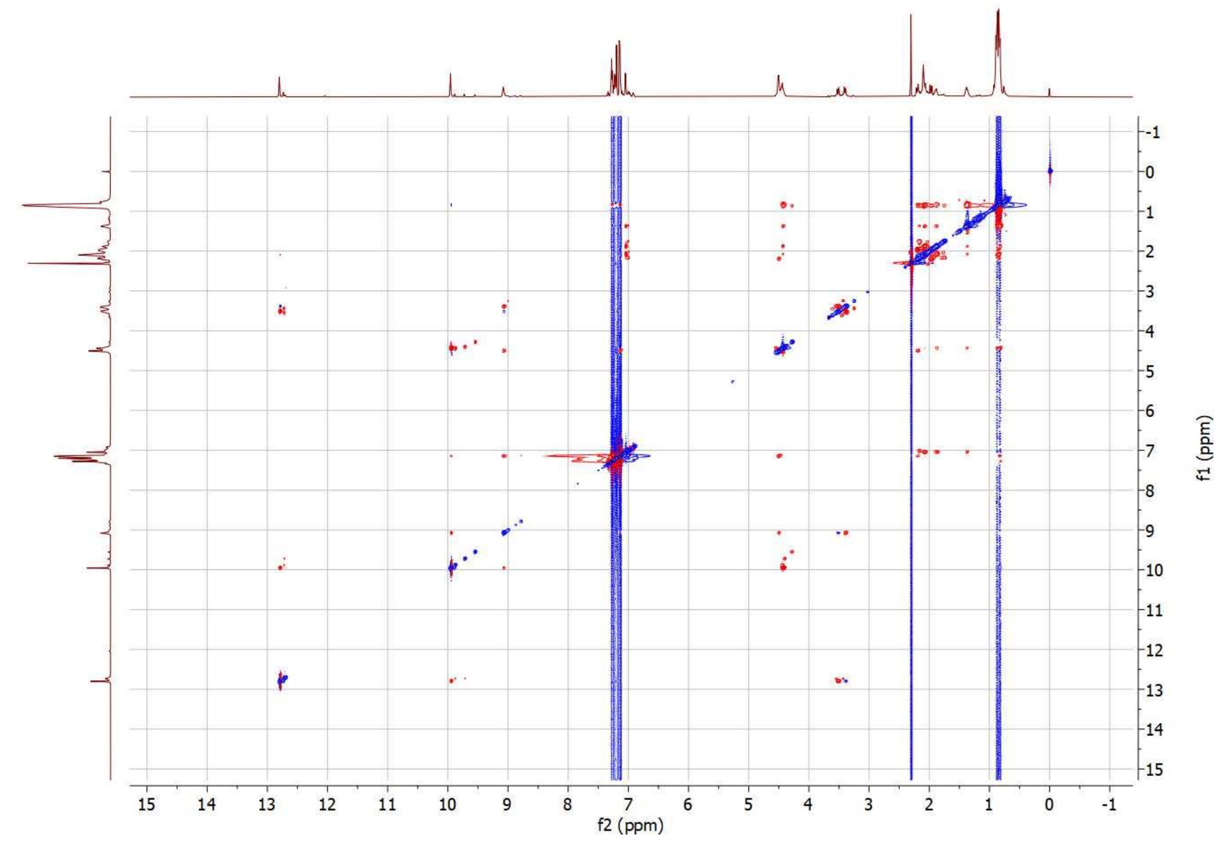

Figure 8. MS spectrum of compound **4**

Elements Used:

C: 0-150 H: 0-150 N: 0-4 O: 0-12

Mass	Calc. Mass	mDa	PPM	DBE	Formula	i-FIT	i-FIT Norm	Fit Conf %	C	H	N	O
1677.9934	1677.9920	1.4	0.8	44.5	C108 H133 N4 O12	125.3	0.113	89.32	108	133	4	12
	1677.9960	-2.6	-1.5	48.5	C113 H133 N2 O10	127.5	2.271	10.32	113	133	2	10
	1677.9942	-0.8	-0.5	61.5	C125 H129 O3	130.8	5.629	0.36	125	129		3

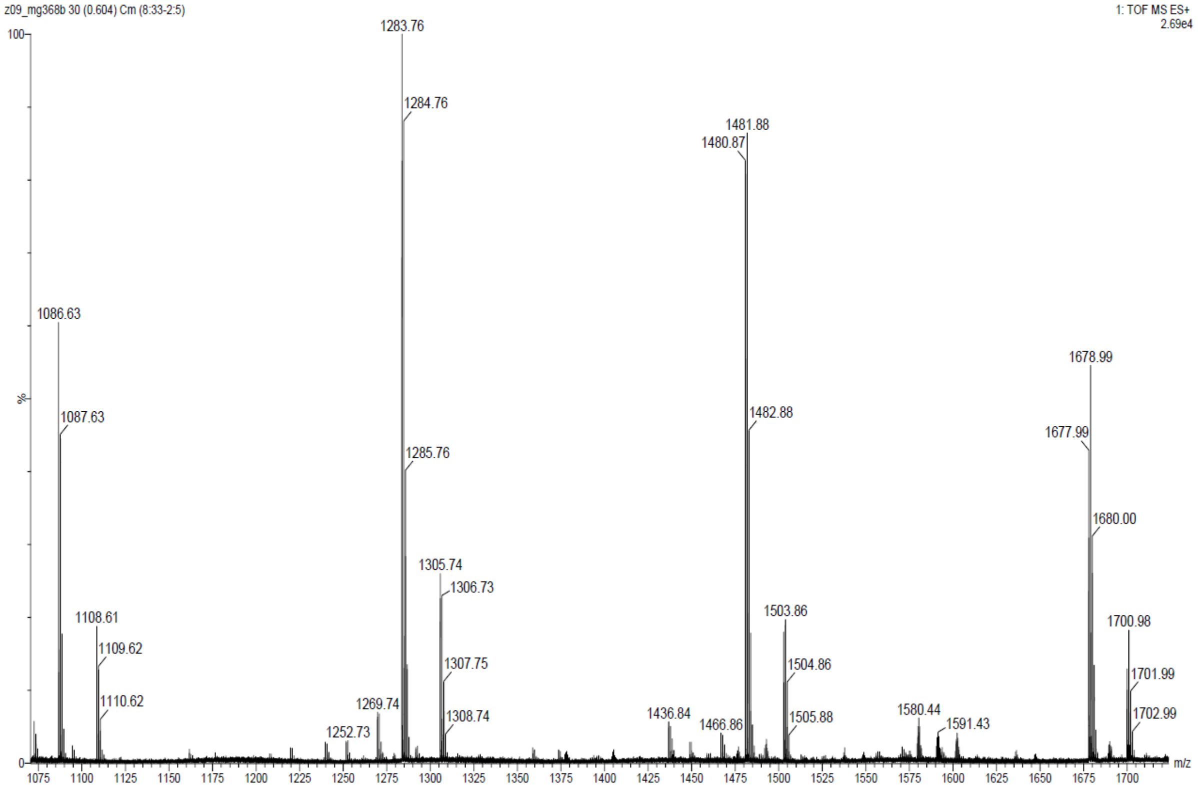

8. DFT/B3LYP calculations

Atomic coordinates for all calculated geometries:

C_2 in chloroform
Energy total = -5317.11813806 a.u.

Symbol	X	Y	Z
C	-1.3491330	-2.9464780	2.1570030
C	-1.3449210	-3.7308710	0.9889420
C	-0.1530780	-4.1716720	0.3871470
C	1.0656170	-3.8185420	0.9912920
C	1.1096740	-3.0314730	2.1569990
C	-0.1050600	-2.6004990	2.6934500
O	-2.5125620	-4.1244850	0.3794950
O	2.2610350	-4.1940890	0.4369670
H	-0.0778170	-1.9834510	3.5853680
C	2.4592140	-2.6577740	2.7826770
C	2.9931880	-1.3484160	2.1831600
C	3.8851810	-1.3594790	1.0944840
C	4.3873430	-0.1749010	0.5279340
C	3.9631440	1.0522480	1.0674770
C	3.0810500	1.1094590	2.1618740
C	2.6046110	-0.0982320	2.6737750
O	4.3500490	-2.5485840	0.5697840
H	1.9103970	-0.0611120	3.5072510
C	2.6724660	2.4615760	2.7621390
C	1.3491330	2.9464780	2.1570030
C	1.3449210	3.7308710	0.9889420
C	0.1530780	4.1716720	0.3871470
C	-1.0656170	3.8185420	0.9912920
C	-1.1096740	3.0314730	2.1569990
C	0.1050600	2.6004990	2.6934500
O	2.5125620	4.1244850	0.3794950
O	-2.2610350	4.1940890	0.4369670
H	0.0778170	1.9834510	3.5853680
C	-2.4592140	2.6577740	2.7826770
C	-2.9931880	1.3484160	2.1831600
C	-3.8851810	1.3594790	1.0944840
C	-4.3873430	0.1749010	0.5279340
C	-3.9631440	-1.0522480	1.0674770
C	-3.0810500	-1.1094590	2.1618740
C	-2.6046110	0.0982320	2.6737750
O	-4.3500490	2.5485840	0.5697840
O	-4.4261580	-2.2440850	0.5632060
H	-1.9103970	0.0611120	3.5072510
C	-2.6724660	-2.4615760	2.7621390
C	2.4218100	-2.6924710	4.3343640
C	2.7220470	2.4473870	4.3139200
H	3.4418630	3.1766750	2.4564150
C	-2.4218100	2.6924710	4.3343640
H	-3.1568420	3.4491350	2.4924210
C	-2.7220470	-2.4473870	4.3139200
H	-3.4418630	-3.1766750	2.4564150
H	1.6868360	-3.4514930	4.6265070
H	2.0525760	-1.7449580	4.7461550
C	3.7533610	-3.0550470	5.0265610
H	3.5059640	1.7394920	4.6066600
H	1.7918330	2.0524590	4.7410300
C	3.0506640	3.7989660	4.9838770
H	-1.6868360	3.4514930	4.6265070
H	-2.0525760	1.7449580	4.7461550
C	-3.7533610	3.0550470	5.0265610
H	-3.5059640	-1.7394920	4.6066600
H	-1.7918330	-2.0524590	4.7410300
C	-3.0506640	-3.7989660	4.9838770
H	3.1568420	-3.4491350	2.4924210
C	-5.4076330	0.2254670	-0.6035870
H	3.6207510	-3.2175020	0.5746350
H	3.2396340	3.4624040	0.4924120
H	-2.2004110	4.9865160	-0.1928600

H	-3.6207510	3.2175020	0.5746350
H	-4.7523430	-2.1680430	-0.3933580
H	-3.2396340	-3.4624040	0.4924120
O	4.4261580	2.2440850	0.5632060
C	-0.1962300	-4.8955600	-0.9524180
H	2.2004110	-4.9865160	-0.1928600
C	5.4076330	-0.2254670	-0.6035870
H	4.7523430	2.1680430	-0.3933580
C	-0.0721450	-6.4154880	-0.9827260
C	-1.1825410	-7.2657880	-0.9483680
C	1.2319040	-6.9726230	-1.1152210
O	2.2778490	-6.2619870	-1.0580950
C	4.8909130	-0.1086690	-2.0356470
C	4.5810250	-1.2283560	-2.8156420
C	4.8121090	1.1977890	-2.6016310
O	5.0380310	2.2417100	-1.9275620
C	0.1962300	4.8955600	-0.9524180
C	0.0721450	6.4154880	-0.9827260
C	-1.2319040	6.9726230	-1.1152210
O	-2.2778490	6.2619870	-1.0580950
C	1.1825410	7.2657880	-0.9483680
C	-4.8909130	0.1086690	-2.0356470
C	-4.8121090	-1.1977890	-2.6016310
O	-5.0380310	-2.2417100	-1.9275620
C	-4.5810250	1.2283560	-2.8156420
H	-5.9891020	1.1456140	-0.5018280
H	-6.1183940	-0.5940050	-0.4555300
H	0.6206090	-4.5041880	-1.5682670
H	-1.1250500	-4.6071540	-1.4512590
H	5.9891020	-1.1456140	-0.5018280
H	6.1183940	0.5940050	-0.4555300
H	-0.6206090	4.5041880	-1.5682670
H	1.1250500	4.6071540	-1.4512590
C	4.8823800	-2.0416970	4.7901160
C	3.5200580	-3.2535170	6.5325040
C	-3.2711040	-3.5941370	6.4908640
C	-2.0015220	-4.8936100	4.7419350
C	-3.5200580	3.2535170	6.5325040
C	-4.8823800	2.0416970	4.7901160
C	3.2711040	3.5941370	6.4908640
C	2.0015220	4.8936100	4.7419350
H	4.0801170	-4.0207610	4.6114070
H	4.0015600	4.1485500	4.5536400
H	-4.0801170	4.0207610	4.6114070
H	-4.0015600	-4.1485500	4.5536400
H	5.1290800	-1.9410710	3.7305800
H	4.6025420	-1.0480320	5.1605200
H	5.7907200	-2.3506750	5.3199190
H	3.1907550	-2.3192460	7.0041550
H	2.7523670	-4.0113440	6.7242140
H	4.4393560	-3.5713760	7.0363590
H	-2.3522530	-3.2430450	6.9768990
H	-4.0543630	-2.8533300	6.6856120
H	-3.5652480	-4.5301200	6.9782370
H	-1.8773600	-5.1157160	3.6793030
H	-1.0219760	-4.5930840	5.1332240
H	-2.2921320	-5.8206740	5.2494050
H	-3.1907550	2.3192460	7.0041550
H	-2.7523670	4.0113440	6.7242140
H	-4.4393560	3.5713760	7.0363590
H	-5.1290800	1.9410710	3.7305800
H	-4.6025420	1.0480320	5.1605200
H	-5.7907200	2.3506750	5.3199190
H	2.3522530	3.2430450	6.9768990
H	4.0543630	2.8533300	6.6856120
H	3.5652480	4.5301200	6.9782370
H	1.8773600	5.1157160	3.6793030
H	1.0219760	4.5930840	5.1332240
H	2.2921320	5.8206740	5.2494050
C	-1.4234050	8.4663540	-1.2917160
H	-1.6124260	8.8885950	-0.2936120
H	-2.3411810	8.6190040	-1.8690590
C	-0.2261450	9.1810480	-1.9370260
C	1.0407600	8.7577350	-1.1672040
H	1.9227290	9.1377220	-1.6916250
H	1.0484480	9.2409370	-0.1786470

C	3.5997520	0.2772580	-4.6557670
C	4.2109450	-1.0894550	-4.2786510
H	3.5068600	-1.8767850	-4.5626030
H	5.1194040	-1.2557950	-4.8755260
C	4.5063650	1.3755570	-4.0768810
H	5.4670850	1.3790640	-4.6128490
H	4.0673210	2.3689340	-4.2131960
C	-4.2109450	1.0894550	-4.2786510
H	-3.5068600	1.8767850	-4.5626030
H	-5.1194040	1.2557950	-4.8755260
C	-3.5997520	-0.2772580	-4.6557670
C	-4.5063650	-1.3755570	-4.0768810
H	-5.4670850	-1.3790640	-4.6128490
H	-4.0673210	-2.3689340	-4.2131960
C	1.4234050	-8.4663540	-1.2917160
H	1.6124260	-8.8885950	-0.2936120
H	2.3411810	-8.6190040	-1.8690590
C	0.2261450	-9.1810480	-1.9370260
C	-1.0407600	-8.7577350	-1.1672040
H	-1.9227290	-9.1377220	-1.6916250
H	-1.0484480	-9.2409370	-0.1786470
C	2.1716960	0.4028970	-4.0878350
H	1.7409960	1.3745060	-4.3520100
H	1.5212030	-0.3758030	-4.5007420
H	2.1543810	0.3133810	-2.9982250
C	3.5415220	0.3966630	-6.1878250
H	2.9168270	-0.3930630	-6.6202300
H	3.1126630	1.3601580	-6.4834080
H	4.5392430	0.3208590	-6.6342860
C	0.1050600	-8.7987080	-3.4264480
H	1.0077060	-9.0943790	-3.9715480
H	-0.7467680	-9.3096740	-3.8885420
H	-0.0327680	-7.7224060	-3.5623150
C	0.4001550	-10.7050880	-1.8259310
H	-0.4558350	-11.2316100	-2.2628120
H	1.2995890	-11.0313800	-2.3589130
H	0.4942180	-11.0219400	-0.7814430
C	-0.1050600	8.7987080	-3.4264480
H	-1.0077060	9.0943790	-3.9715480
H	0.7467680	9.3096740	-3.8885420
H	0.0327680	7.7224060	-3.5623150
C	-0.4001550	10.7050880	-1.8259310
H	0.4558350	11.2316100	-2.2628120
H	-1.2995890	11.0313800	-2.3589130
H	-0.4942180	11.0219400	-0.7814430
C	-2.1716960	-0.4028970	-4.0878350
H	-1.7409960	-1.3745060	-4.3520100
H	-1.5212030	0.3758030	-4.5007420
H	-2.1543810	-0.3133810	-2.9982250
C	-3.5415220	-0.3966630	-6.1878250
H	-2.9168270	0.3930630	-6.6202300
H	-3.1126630	-1.3601580	-6.4834080
H	-4.5392430	-0.3208590	-6.6342860
N	2.4197270	6.8051240	-0.6892730
N	4.6361910	-2.4635260	-2.2868210
N	-4.6361910	2.4635260	-2.2868210
N	-2.4197270	-6.8051240	-0.6892730
H	2.5217920	5.8397810	-0.3658560
H	4.7460950	-2.5206920	-1.2722820
H	-2.5217920	-5.8397810	-0.3658560
H	-4.7460950	2.5206920	-1.2722820
C	-4.4726690	3.7620310	-2.9409880
H	-3.5849240	4.2586970	-2.5333790
H	-4.3002130	3.6116000	-4.0073640
C	3.6372140	7.6255040	-0.6536640
H	3.8061000	8.0633590	-1.6467710
H	3.5111780	8.4611210	0.0440010
C	4.4726690	-3.7620310	-2.9409880
H	3.5849240	-4.2586970	-2.5333790
H	4.3002130	-3.6116000	-4.0073640
C	-3.6372140	-7.6255040	-0.6536640
H	-3.8061000	-8.0633590	-1.6467710
H	-3.5111780	-8.4611210	0.0440010
C	-5.6856510	4.6578790	-2.7392990
C	-5.5470880	5.9052740	-2.1200340
C	-6.9525090	4.2531880	-3.1831180

C	-6.6585970	6.7372580	-1.9548680
H	-4.5694730	6.2230000	-1.7652140
C	-8.0604890	5.0822930	-3.0164610
H	-7.0722460	3.2823940	-3.6572060
C	-7.9155670	6.3296070	-2.4014860
H	-6.5401190	7.7026120	-1.4708660
H	-9.0363790	4.7573240	-3.3658080
H	-8.7787440	6.9756970	-2.2702080
C	4.8564070	6.8225940	-0.2508990
C	5.2194320	5.6561480	-0.9383540
C	5.6696780	7.2706730	0.7963970
C	6.3683750	4.9507210	-0.5806800
H	4.5999930	5.2817300	-1.7483710
C	6.8277160	6.5737860	1.1475850
H	5.3953270	8.1708850	1.3403310
C	7.1784450	5.4115090	0.4600480
H	6.6135490	4.0379580	-1.1129530
H	7.4481010	6.9354700	1.9624210
H	8.0740180	4.8632270	0.7373080
C	5.6856510	-4.6578790	-2.7392990
C	5.5470880	-5.9052740	-2.1200340
C	6.9525090	-4.2531880	-3.1831180
C	6.6585970	-6.7372580	-1.9548680
H	4.5694730	-6.2230000	-1.7652140
C	8.0604890	-5.0822930	-3.0164610
H	7.0722460	-3.2823940	-3.6572060
C	7.9155670	-6.3296070	-2.4014860
H	6.5401190	-7.7026120	-1.4708660
H	9.0363790	-4.7573240	-3.3658080
H	8.7787440	-6.9756970	-2.2702080
C	-4.8564070	-6.8225940	-0.2508990
C	-5.2194320	-5.6561480	-0.9383540
C	-5.6696780	-7.2706730	0.7963970
C	-6.3683750	-4.9507210	-0.5806800
H	-4.5999930	-5.2817300	-1.7483710
C	-6.8277160	-6.5737860	1.1475850
H	-5.3953270	-8.1708850	1.3403310
C	-7.1784450	-5.4115090	0.4600480
H	-6.6135490	-4.0379580	-1.1129530
H	-7.4481010	-6.9354700	1.9624210
H	-8.0740180	-4.8632270	0.7373080

C_4 crown-*in* in chloroform
Energy total = -5317.12025046 a.u.

Symbol	X	Y	Z
C	-3.1904160	0.6589660	2.5593730
C	-3.9027730	1.1564500	1.4515960
C	-3.6405550	2.4307770	0.9150080
C	-2.5736440	3.1705140	1.4559150
C	-1.8195910	2.6986970	2.5434690
C	-2.1533100	1.4442800	3.0617880
O	-4.9042180	0.3713690	0.9274550
O	-2.3349290	4.4169470	0.9169050
H	-1.5867440	1.0740740	3.9095060
C	-0.7047140	3.5509730	3.1594640
C	0.6589660	3.1904160	2.5593730
C	1.1564500	3.9027730	1.4515960
C	2.4307770	3.6405550	0.9150080
C	3.1705140	2.5736440	1.4559150
C	2.6986970	1.8195910	2.5434690
C	1.4442800	2.1533100	3.0617880
O	0.3713690	4.9042180	0.9274550
H	1.0740740	1.5867440	3.9095060
C	3.5509730	0.7047140	3.1594640
C	3.1904160	-0.6589660	2.5593730
C	3.9027730	-1.1564500	1.4515960
C	3.6405550	-2.4307770	0.9150080
C	2.5736440	-3.1705140	1.4559150
C	1.8195910	-2.6986970	2.5434690
C	2.1533100	-1.4442800	3.0617880
O	4.9042180	-0.3713690	0.9274550
O	2.3349290	-4.4169470	0.9169050
H	1.5867440	-1.0740740	3.9095060
C	0.7047140	-3.5509730	3.1594640

C	-0.6589660	-3.1904160	2.5593730
C	-1.1564500	-3.9027730	1.4515960
C	-2.4307770	-3.6405550	0.9150080
C	-3.1705140	-2.5736440	1.4559150
C	-2.6986970	-1.8195910	2.5434690
C	-1.4442800	-2.1533100	3.0617880
O	-0.3713690	-4.9042180	0.9274550
O	-4.4169470	-2.3349290	0.9169050
H	-1.0740740	-1.5867440	3.9095060
C	-3.5509730	-0.7047140	3.1594640
C	-0.7569650	3.5427410	4.7121040
C	3.5427410	0.7569650	4.7121040
H	4.5864780	0.9097300	2.8735870
C	0.7569650	-3.5427410	4.7121040
H	0.9097300	-4.5864780	2.8735870
C	-3.5427410	-0.7569650	4.7121040
H	-4.5864780	-0.9097300	2.8735870
H	-1.8083380	3.4279210	5.0000740
H	-0.2374540	2.6689830	5.1250880
C	-0.2285540	4.8172850	5.4057050
H	3.4279210	1.8083380	5.0000740
H	2.6689830	0.2374540	5.1250880
C	4.8172850	0.2285540	5.4057050
H	1.8083380	-3.4279210	5.0000740
H	0.2374540	-2.6689830	5.1250880
C	0.2285540	-4.8172850	5.4057050
H	-3.4279210	-1.8083380	5.0000740
H	-2.6689830	-0.2374540	5.1250880
C	-4.8172850	-0.2285540	5.4057050
H	-0.9097300	4.5864780	2.8735870
C	-3.0460950	-4.5625830	-0.1339210
H	0.5830230	5.0480130	-0.0517280
H	5.0480130	-0.5830230	-0.0517280
H	1.3730540	-4.6326780	0.9730320
H	-0.5830230	-5.0480130	-0.0517280
H	-4.6326780	-1.3730540	0.9730320
H	-5.0480130	0.5830230	-0.0517280
O	4.4169470	2.3349290	0.9169050
C	-4.5625830	3.0460950	-0.1339210
H	-1.3730540	4.6326780	0.9730320
C	3.0460950	4.5625830	-0.1339210
H	4.6326780	1.3730540	0.9730320
C	-4.2363990	2.8717840	-1.6135060
C	-3.6238490	3.8773380	-2.3718280
C	-4.6590670	1.6705930	-2.2465820
O	-5.1903280	0.7155070	-1.6088910
C	2.8717840	4.2363990	-1.6135060
C	3.8773380	3.6238490	-2.3718280
C	1.6705930	4.6590670	-2.2465820
O	0.7155070	5.1903280	-1.6088910
C	4.5625830	-3.0460950	-0.1339210
C	4.2363990	-2.8717840	-1.6135060
C	4.6590670	-1.6705930	-2.2465820
O	5.1903280	-0.7155070	-1.6088910
C	3.6238490	-3.8773380	-2.3718280
C	-2.8717840	-4.2363990	-1.6135060
C	-1.6705930	-4.6590670	-2.2465820
O	-0.7155070	-5.1903280	-1.6088910
C	-3.8773380	-3.6238490	-2.3718280
H	-2.6217020	-5.5600500	0.0218880
H	-4.1130490	-4.6583730	0.0864900
H	-5.5600500	2.6217020	0.0218880
H	-4.6583730	4.1130490	0.0864900
H	2.6217020	5.5600500	0.0218880
H	4.1130490	4.6583730	0.0864900
H	5.5600500	-2.6217020	0.0218880
H	4.6583730	-4.1130490	0.0864900
C	1.2706670	5.0744470	5.1965020
C	-0.5559270	4.7664570	6.9066130
C	-4.7664570	-0.5559270	6.9066130
C	-5.0744470	1.2706670	5.1965020
C	0.5559270	-4.7664570	6.9066130
C	-1.2706670	-5.0744470	5.1965020
C	4.7664570	0.5559270	6.9066130
C	5.0744470	-1.2706670	5.1965020
H	-0.7769240	5.6691200	4.9754290

H	5.6691200	0.7769240	4.9754290
H	0.7769240	-5.6691200	4.9754290
H	-5.6691200	-0.7769240	4.9754290
H	1.5249530	5.1945230	4.1409210
H	1.8694970	4.2434120	5.5883760
H	1.5794160	5.9842970	5.7239360
H	-0.0382320	3.9299260	7.3924790
H	-1.6302090	4.6385440	7.0794780
H	-0.2409190	5.6866480	7.4106190
H	-3.9299260	-0.0382320	7.3924790
H	-4.6385440	-1.6302090	7.0794780
H	-5.6866480	-0.2409190	7.4106190
H	-5.1945230	1.5249530	4.1409210
H	-4.2434120	1.8694970	5.5883760
H	-5.9842970	1.5794160	5.7239360
H	0.0382320	-3.9299260	7.3924790
H	1.6302090	-4.6385440	7.0794780
H	0.2409190	-5.6866480	7.4106190
H	-1.5249530	-5.1945230	4.1409210
H	-1.8694970	-4.2434120	5.5883760
H	-1.5794160	-5.9842970	5.7239360
H	3.9299260	0.0382320	7.3924790
H	4.6385440	1.6302090	7.0794780
H	5.6866480	0.2409190	7.4106190
H	5.1945230	-1.5249530	4.1409210
H	4.2434120	-1.8694970	5.5883760
H	5.9842970	-1.5794160	5.7239360
C	4.4539920	-1.4727240	-3.7370080
H	5.2308580	-0.7878660	-4.0923870
H	3.4934930	-0.9527660	-3.8688850
C	1.4727240	4.4539920	-3.7370080
H	0.7878660	5.2308580	-4.0923870
H	0.9527660	3.4934930	-3.8688850
C	-4.4539920	1.4727240	-3.7370080
H	-5.2308580	0.7878660	-4.0923870
H	-3.4934930	0.9527660	-3.8688850
C	-1.4727240	-4.4539920	-3.7370080
H	-0.7878660	-5.2308580	-4.0923870
H	-0.9527660	-3.4934930	-3.8688850
C	-2.7789170	-4.4512080	-4.5472530
C	-3.7492150	-3.4628010	-3.8708800
H	-3.4189590	-2.4306860	-4.0611220
H	-4.7334320	-3.5509120	-4.3401860
C	-4.4512080	2.7789170	-4.5472530
C	-3.4628010	3.7492150	-3.8708800
H	-2.4306860	3.4189590	-4.0611220
H	-3.5509120	4.7334320	-4.3401860
C	2.7789170	4.4512080	-4.5472530
C	3.7492150	3.4628010	-3.8708800
H	3.4189590	2.4306860	-4.0611220
H	4.7334320	3.5509120	-4.3401860
C	4.4512080	-2.7789170	-4.5472530
C	3.4628010	-3.7492150	-3.8708800
H	2.4306860	-3.4189590	-4.0611220
H	3.5509120	-4.7334320	-4.3401860
C	5.8654600	-3.3928670	-4.5857980
H	5.8675850	-4.3229070	-5.1648550
H	6.5701320	-2.7020560	-5.0608260
H	6.2401860	-3.6186180	-3.5834440
C	3.9860310	-2.5038800	-5.9870180
H	2.9845830	-2.0597460	-6.0049780
H	4.6704900	-1.8122280	-6.4899020
H	3.9558170	-3.4283020	-6.5745390
C	-3.3928670	-5.8654600	-4.5857980
H	-4.3229070	-5.8675850	-5.1648550
H	-2.7020560	-6.5701320	-5.0608260
H	-3.6186180	-6.2401860	-3.5834440
C	-2.5038800	-3.9860310	-5.9870180
H	-2.0597460	-2.9845830	-6.0049780
H	-1.8122280	-4.6704900	-6.4899020
H	-3.4283020	-3.9558170	-6.5745390
C	-5.8654600	3.3928670	-4.5857980
H	-5.8675850	4.3229070	-5.1648550
H	-6.5701320	2.7020560	-5.0608260
H	-6.2401860	3.6186180	-3.5834440
C	-3.9860310	2.5038800	-5.9870180

H	-2.9845830	2.0597460	-6.0049780
H	-4.6704900	1.8122280	-6.4899020
H	-3.9558170	3.4283020	-6.5745390
C	3.3928670	5.8654600	-4.5857980
H	4.3229070	5.8675850	-5.1648550
H	2.7020560	6.5701320	-5.0608260
H	3.6186180	6.2401860	-3.5834440
C	2.5038800	3.9860310	-5.9870180
H	2.0597460	2.9845830	-6.0049780
H	1.8122280	4.6704900	-6.4899020
H	3.4283020	3.9558170	-6.5745390
N	-3.1382420	4.9816550	-1.7789860
N	-4.9816550	-3.1382420	-1.7789860
N	3.1382420	-4.9816550	-1.7789860
N	4.9816550	3.1382420	-1.7789860
H	4.9221210	3.0045330	-0.7677160
H	-3.0045330	4.9221210	-0.7677160
H	-4.9221210	-3.0045330	-0.7677160
H	3.0045330	-4.9221210	-0.7677160
C	2.4307770	-6.1047690	-2.4072250
H	1.3775310	-5.8424450	-2.5565330
H	2.8771080	-6.3105120	-3.3832150
C	6.1047690	2.4307770	-2.4072250
H	5.8424450	1.3775310	-2.5565330
H	6.3105120	2.8771080	-3.3832150
C	-2.4307770	6.1047690	-2.4072250
H	-1.3775310	5.8424450	-2.5565330
H	-2.8771080	6.3105120	-3.3832150
C	-6.1047690	-2.4307770	-2.4072250
H	-5.8424450	-1.3775310	-2.5565330
H	-6.3105120	-2.8771080	-3.3832150
C	2.5290610	-7.3448770	-1.5399080
C	1.3889980	-7.8670580	-0.9187250
C	3.7620030	-7.9856330	-1.3515420
C	1.4785340	-9.0148220	-0.1262890
H	0.4343780	-7.3679400	-1.0571400
C	3.8526060	-9.1291140	-0.5594510
H	4.6530620	-7.5837380	-1.8272100
C	2.7085800	-9.6474830	0.0547800
H	0.5870190	-9.4103900	0.3516490
H	4.8129730	-9.6177780	-0.4225120
H	2.7785330	-10.5388020	0.6714740
C	7.3448770	2.5290610	-1.5399080
C	7.8670580	1.3889980	-0.9187250
C	7.9856330	3.7620030	-1.3515420
C	9.0148220	1.4785340	-0.1262890
H	7.3679400	0.4343780	-1.0571400
C	9.1291140	3.8526060	-0.5594510
H	7.5837380	4.6530620	-1.8272100
C	9.6474830	2.7085800	0.0547800
H	9.4103900	0.5870190	0.3516490
H	9.6177780	4.8129730	-0.4225120
H	10.5388020	2.7785330	0.6714740
C	-2.5290610	7.3448770	-1.5399080
C	-1.3889980	7.8670580	-0.9187250
C	-3.7620030	7.9856330	-1.3515420
C	-1.4785340	9.0148220	-0.1262890
H	-0.4343780	7.3679400	-1.0571400
C	-3.8526060	9.1291140	-0.5594510
H	-4.6530620	7.5837380	-1.8272100
C	-2.7085800	9.6474830	0.0547800
H	-0.5870190	9.4103900	0.3516490
H	-4.8129730	9.6177780	-0.4225120
H	-2.7785330	10.5388020	0.6714740
C	-7.3448770	-2.5290610	-1.5399080
C	-7.8670580	-1.3889980	-0.9187250
C	-7.9856330	-3.7620030	-1.3515420
C	-9.0148220	-1.4785340	-0.1262890
H	-7.3679400	-0.4343780	-1.0571400
C	-9.1291140	-3.8526060	-0.5594510
H	-7.5837380	-4.6530620	-1.8272100
C	-9.6474830	-2.7085800	0.0547800
H	-9.4103900	-0.5870190	0.3516490
H	-9.6177780	-4.8129730	-0.4225120
H	-10.5388020	-2.7785330	0.6714740

C_4 crown-*out* in chloroform
Energy total = -5317.11776943 a.u.

Symbol	X	Y	Z
C	2.9352710	-1.4179070	1.7840410
C	3.7806720	-1.4406180	0.6585130
C	4.3073730	-0.2637120	0.0972210
C	3.9461890	0.9666730	0.6733420
C	3.0864660	1.0368290	1.7840430
C	2.5944700	-0.1634840	2.2992010
O	4.1532500	-2.6261570	0.0637710
O	4.3826880	2.1495030	0.1351320
H	1.9310410	-0.1181490	3.1568270
C	2.7217020	2.3973210	2.3883550
C	1.4179070	2.9352710	1.7840410
C	1.4406180	3.7806720	0.6585130
C	0.2637120	4.3073730	0.0972210
C	-0.9666730	3.9461890	0.6733420
C	-1.0368290	3.0864660	1.7840430
C	0.1634840	2.5944700	2.2992010
O	2.6261570	4.1532500	0.0637710
H	0.1181490	1.9310410	3.1568270
C	-2.3973210	2.7217020	2.3883550
C	-2.9352710	1.4179070	1.7840410
C	-3.7806720	1.4406180	0.6585130
C	-4.3073730	0.2637120	0.0972210
C	-3.9461890	-0.9666730	0.6733420
C	-3.0864660	-1.0368290	1.7840430
C	-2.5944700	0.1634840	2.2992010
O	-4.1532500	2.6261570	0.0637710
O	-4.3826880	-2.1495030	0.1351320
H	-1.9310410	0.1181490	3.1568270
C	-2.7217020	-2.3973210	2.3883550
C	-1.4179070	-2.9352710	1.7840410
C	-1.4406180	-3.7806720	0.6585130
C	-0.2637120	-4.3073730	0.0972210
C	0.9666730	-3.9461890	0.6733420
C	1.0368290	-3.0864660	1.7840430
C	-0.1634840	-2.5944700	2.2992010
O	-2.6261570	-4.1532500	0.0637710
O	2.1495030	-4.3826880	0.1351320
H	-0.1181490	-1.9310410	3.1568270
C	2.3973210	-2.7217020	2.3883550
C	2.7616330	2.3785190	3.9405860
C	-2.3785190	2.7616330	3.9405860
H	-3.0836260	3.5169270	2.0824970
C	-2.7616330	-2.3785190	3.9405860
H	-3.5169270	-3.0836260	2.0824970
C	2.3785190	-2.7616330	3.9405860
H	3.0836260	-3.5169270	2.0824970
H	3.5169460	1.6416090	4.2373800
H	1.8141900	2.0190560	4.3610960
C	3.1359960	3.7156710	4.6155000
H	-1.6416090	3.5169460	4.2373800
H	-2.0190560	1.8141900	4.3610960
C	-3.7156710	3.1359960	4.6155000
H	-3.5169460	-1.6416090	4.2373800
H	-1.8141900	-2.0190560	4.3610960
C	-3.1359960	-3.7156710	4.6155000
H	1.6416090	-3.5169460	4.2373800
H	2.0190560	-1.8141900	4.3610960
C	3.7156710	-3.1359960	4.6155000
H	3.5169270	3.0836260	2.0824970
C	-0.3160020	-5.1834580	-1.1469810
H	3.2980180	3.4261310	0.0842600
H	-3.4261310	3.2980180	0.0842600
H	-5.2716140	-2.0761980	-0.3543160
H	-3.2980180	-3.4261310	0.0842600
H	2.0761980	-5.2716140	-0.3543160
H	3.4261310	-3.2980180	0.0842600
O	-2.1495030	4.3826880	0.1351320
C	5.1834580	-0.3160020	-1.1469810
H	5.2716140	2.0761980	-0.3543160
C	0.3160020	5.1834580	-1.1469810
H	-2.0761980	5.2716140	-0.3543160

C	6.6972570	-0.2816520	-0.9507770
C	7.4661790	-1.4386950	-0.8232880
C	7.3393350	0.9937920	-0.9229410
O	6.6972570	2.0794810	-0.9274220
C	0.2816520	6.6972570	-0.9507770
C	1.4386950	7.4661790	-0.8232880
C	-0.9937920	7.3393350	-0.9229410
O	-2.0794810	6.6972570	-0.9274220
C	-5.1834580	0.3160020	-1.1469810
C	-6.6972570	0.2816520	-0.9507770
C	-7.3393350	-0.9937920	-0.9229410
O	-6.6972570	-2.0794810	-0.9274220
C	-7.4661790	1.4386950	-0.8232880
C	-0.2816520	-6.6972570	-0.9507770
C	0.9937920	-7.3393350	-0.9229410
O	2.0794810	-6.6972570	-0.9274220
C	-1.4386950	-7.4661790	-0.8232880
H	0.5403930	-4.9261310	-1.7786720
H	-1.2103090	-4.9132520	-1.7150090
H	4.9261310	0.5403930	-1.7786720
H	4.9132520	-1.2103090	-1.7150090
H	-0.5403930	4.9261310	-1.7786720
H	1.2103090	4.9132520	-1.7150090
H	-4.9261310	-0.5403930	-1.7786720
H	-4.9132520	1.2103090	-1.7150090
C	2.1253990	4.8464680	4.3756020
C	3.3465750	3.4985740	6.1222010
C	3.4985740	-3.3465750	6.1222010
C	4.8464680	-2.1253990	4.3756020
C	-3.3465750	-3.4985740	6.1222010
C	-2.1253990	-4.8464680	4.3756020
C	-3.4985740	3.3465750	6.1222010
C	-4.8464680	2.1253990	4.3756020
H	4.0995240	4.0333500	4.1882810
H	-4.0333500	4.0995240	4.1882810
H	-4.0995240	-4.0333500	4.1882810
H	4.0333500	-4.0995240	4.1882810
H	2.0096510	5.0757940	3.3135700
H	1.1357410	4.5777480	4.7645310
H	2.4462560	5.7614790	4.8867050
H	2.4156670	3.1758940	6.6049330
H	4.1046430	2.7316680	6.3157680
H	3.6701110	4.4226090	6.6137030
H	3.1758940	-2.4156670	6.6049330
H	2.7316680	-4.1046430	6.3157680
H	4.4226090	-3.6701110	6.6137030
H	5.0757940	-2.0096510	3.3135700
H	4.5777480	-1.1357410	4.7645310
H	5.7614790	-2.4462560	4.8867050
H	-2.4156670	-3.1758940	6.6049330
H	-4.1046430	-2.7316680	6.3157680
H	-3.6701110	-4.4226090	6.6137030
H	-2.0096510	-5.0757940	3.3135700
H	-1.1357410	-4.5777480	4.7645310
H	-2.4462560	-5.7614790	4.8867050
H	-3.1758940	2.4156670	6.6049330
H	-2.7316680	4.1046430	6.3157680
H	-4.4226090	3.6701110	6.6137030
H	-5.0757940	2.0096510	3.3135700
H	-4.5777480	1.1357410	4.7645310
H	-5.7614790	2.4462560	4.8867050
C	-8.8519720	-1.0910720	-0.8425030
H	-9.1077980	-1.2212600	0.2196420
H	-9.1515140	-2.0161120	-1.3462980
C	-9.5982490	0.1290960	-1.4051510
C	-8.9778320	1.3881340	-0.7663490
H	-9.3912660	2.2805990	-1.2453020
H	-9.2735660	1.4427530	0.2921830
C	0.1290960	9.5982490	-1.4051510
C	1.3881340	8.9778320	-0.7663490
H	2.2805990	9.3912660	-1.2453020
H	1.4427530	9.2735660	0.2921830
C	-1.0910720	8.8519720	-0.8425030
H	-1.2212600	9.1077980	0.2196420
H	-2.0161120	9.1515140	-1.3462980
C	-1.3881340	-8.9778320	-0.7663490

H	-2.2805990	-9.3912660	-1.2453020
H	-1.4427530	-9.2735660	0.2921830
C	-0.1290960	-9.5982490	-1.4051510
C	1.0910720	-8.8519720	-0.8425030
H	1.2212600	-9.1077980	0.2196420
H	2.0161120	-9.1515140	-1.3462980
C	8.8519720	1.0910720	-0.8425030
H	9.1077980	1.2212600	0.2196420
H	9.1515140	2.0161120	-1.3462980
C	9.5982490	-0.1290960	-1.4051510
C	8.9778320	-1.3881340	-0.7663490
H	9.3912660	-2.2805990	-1.2453020
H	9.2735660	-1.4427530	0.2921830
C	0.1831730	9.4713580	-2.9411390
H	-0.7128420	9.9107480	-3.3923740
H	1.0547710	9.9998170	-3.3421570
H	0.2441160	8.4282000	-3.2630920
C	0.0531140	11.0876610	-1.0296610
H	0.9262160	11.6328370	-1.4054290
H	-0.8405040	11.5504840	-1.4618990
H	0.0106380	11.2242250	0.0566350
C	9.4713580	-0.1831730	-2.9411390
H	9.9107480	0.7128420	-3.3923740
H	9.9998170	-1.0547710	-3.3421570
H	8.4282000	-0.2441160	-3.2630920
C	11.0876610	-0.0531140	-1.0296610
H	11.6328370	-0.9262160	-1.4054290
H	11.5504840	0.8405040	-1.4618990
H	11.2242250	-0.0106380	0.0566350
C	-9.4713580	0.1831730	-2.9411390
H	-9.9107480	-0.7128420	-3.3923740
H	-9.9998170	1.0547710	-3.3421570
H	-8.4282000	0.2441160	-3.2630920
C	-11.0876610	0.0531140	-1.0296610
H	-11.6328370	0.9262160	-1.4054290
H	-11.5504840	-0.8405040	-1.4618990
H	-11.2242250	0.0106380	0.0566350
C	-0.1831730	-9.4713580	-2.9411390
H	0.7128420	-9.9107480	-3.3923740
H	-1.0547710	-9.9998170	-3.3421570
H	-0.2441160	-8.4282000	-3.2630920
C	-0.0531140	-11.0876610	-1.0296610
H	-0.9262160	-11.6328370	-1.4054290
H	0.8405040	-11.5504840	-1.4618990
H	-0.0106380	-11.2242250	0.0566350
N	-6.8878370	2.6512760	-0.7100220
N	2.6512760	6.8878370	-0.7100220
N	-2.6512760	-6.8878370	-0.7100220
N	6.8878370	-2.6512760	-0.7100220
H	-5.8768870	2.6825700	-0.5717030
H	2.6825700	5.8768870	-0.5717030
H	5.8768870	-2.6825700	-0.5717030
H	-2.6825700	-5.8768870	-0.5717030
C	-3.9288270	-7.5744230	-0.5470700
H	-3.8423770	-8.3820000	0.1874740
H	-4.6072210	-6.8358860	-0.1062080
C	-7.5744230	3.9288270	-0.5470700
H	-8.3820000	3.8423770	0.1874740
H	-6.8358860	4.6072210	-0.1062080
C	3.9288270	7.5744230	-0.5470700
H	3.8423770	8.3820000	0.1874740
H	4.6072210	6.8358860	-0.1062080
C	7.5744230	-3.9288270	-0.5470700
H	8.3820000	-3.8423770	0.1874740
H	6.8358860	-4.6072210	-0.1062080
C	-4.5464630	-8.1173620	-1.8300780
C	-5.3793330	-9.2420200	-1.7722560
C	-4.3427940	-7.4894400	-3.0643890
C	-6.0030380	-9.7266620	-2.9230870
H	-5.5410140	-9.7419150	-0.8200360
C	-4.9626150	-7.9749870	-4.2169660
H	-3.6930910	-6.6213600	-3.1220390
C	-5.7958150	-9.0937150	-4.1501990
H	-6.6438860	-10.6014840	-2.8615530
H	-4.7943230	-7.4778080	-5.1678940
H	-6.2764800	-9.4713190	-5.0478050

C	-8.1173620	4.5464630	-1.8300780
C	-9.2420200	5.3793330	-1.7722560
C	-7.4894400	4.3427940	-3.0643890
C	-9.7266620	6.0030380	-2.9230870
H	-9.7419150	5.5410140	-0.8200360
C	-7.9749870	4.9626150	-4.2169660
H	-6.6213600	3.6930910	-3.1220390
C	-9.0937150	5.7958150	-4.1501990
H	-10.6014840	6.6438860	-2.8615530
H	-7.4778080	4.7943230	-5.1678940
H	-9.4713190	6.2764800	-5.0478050
C	4.5464630	8.1173620	-1.8300780
C	5.3793330	9.2420200	-1.7722560
C	4.3427940	7.4894400	-3.0643890
C	6.0030380	9.7266620	-2.9230870
H	5.5410140	9.7419150	-0.8200360
C	4.9626150	7.9749870	-4.2169660
H	3.6930910	6.6213600	-3.1220390
C	5.7958150	9.0937150	-4.1501990
H	6.6438860	10.6014840	-2.8615530
H	4.7943230	7.4778080	-5.1678940
H	6.2764800	9.4713190	-5.0478050
C	8.1173620	-4.5464630	-1.8300780
C	9.2420200	-5.3793330	-1.7722560
C	7.4894400	-4.3427940	-3.0643890
C	9.7266620	-6.0030380	-2.9230870
H	9.7419150	-5.5410140	-0.8200360
C	7.9749870	-4.9626150	-4.2169660
H	6.6213600	-3.6930910	-3.1220390
C	9.0937150	-5.7958150	-4.1501990
H	10.6014840	-6.6438860	-2.8615530
H	7.4778080	-4.7943230	-5.1678940
H	9.4713190	-6.2764800	-5.0478050

C_2 in DMSO

Energy total = -5317.13125737 a.u.

Symbol	X	Y	Z
C	1.4239220	2.9068970	2.1341880
C	1.4362780	3.6866290	0.9623980
C	0.2542870	4.1602900	0.3654790
C	-0.9713690	3.8425870	0.9768720
C	-1.0317090	3.0589210	2.1449050
C	0.1732930	2.5968680	2.6782980
O	2.6121530	4.0422840	0.3456260
O	-2.1569170	4.2502830	0.4270500
H	0.1337230	1.9868180	3.5745310
C	-2.3884880	2.7214600	2.7759800
C	-2.9580980	1.4284580	2.1731210
C	-3.8501290	1.4673380	1.0842980
C	-4.3804090	0.2980980	0.5111740
C	-3.9868410	-0.9423550	1.0448650
C	-3.1091660	-1.0268660	2.1415040
C	-2.6027620	0.1664850	2.6599610
O	-4.2870300	2.6701850	0.5677300
H	-1.9132120	0.1084810	3.4961190
C	-2.7365070	-2.3913540	2.7376330
C	-1.4239220	-2.9068970	2.1341880
C	-1.4362780	-3.6866290	0.9623980
C	-0.2542870	-4.1602900	0.3654790
C	0.9713690	-3.8425870	0.9768720
C	1.0317090	-3.0589210	2.1449050
C	-0.1732930	-2.5968680	2.6782980
O	-2.6121530	-4.0422840	0.3456260
O	2.1569170	-4.2502830	0.4270500
H	-0.1337230	-1.9868180	3.5745310
C	2.3884880	-2.7214600	2.7759800
C	2.9580980	-1.4284580	2.1731210
C	3.8501290	-1.4673380	1.0842980
C	4.3804090	-0.2980980	0.5111740
C	3.9868410	0.9423550	1.0448650
C	3.1091660	1.0268660	2.1415040
C	2.6027620	-0.1664850	2.6599610
O	4.2870300	-2.6701850	0.5677300
O	4.4791330	2.1175920	0.5299250

H	1.9132120	-0.1084810	3.4961190
C	2.7365070	2.3913540	2.7376330
C	-2.3447310	2.7510870	4.3275560
C	-2.7915830	-2.3827300	4.2894540
H	-3.5222070	-3.0857430	2.4254360
C	2.3447310	-2.7510870	4.3275560
H	3.0655730	-3.5314150	2.4883450
C	2.7915830	2.3827300	4.2894540
H	3.5222070	3.0857430	2.4254360
H	-1.5893220	3.4898810	4.6192190
H	-1.9985280	1.7937220	4.7360950
C	-3.6638430	3.1478120	5.0249680
H	-3.5608180	-1.6589780	4.5825110
H	-1.8549910	-2.0096760	4.7216730
C	-3.1538380	-3.7292390	4.9524910
H	1.5893220	-3.4898810	4.6192190
H	1.9985280	-1.7937220	4.7360950
C	3.6638430	-3.1478120	5.0249680
H	3.5608180	1.6589780	4.5825110
H	1.8549910	2.0096760	4.7216730
C	3.1538380	3.7292390	4.9524910
H	-3.0655730	3.5314150	2.4883450
C	5.3994320	-0.3777920	-0.6196980
H	-3.5375980	3.3180110	0.5626090
H	-3.3202740	-3.3607520	0.4635900
H	2.0785030	-5.0476050	-0.2033990
H	3.5375980	-3.3180110	0.5626090
H	4.7741910	2.0270080	-0.4410310
H	3.3202740	3.3607520	0.4635900
O	-4.4791330	-2.1175920	0.5299250
C	0.3082970	4.8849350	-0.9736750
H	-2.0785030	5.0476050	-0.2033990
C	-5.3994320	0.3777920	-0.6196980
H	-4.7741910	-2.0270080	-0.4410310
C	0.2264430	6.4081840	-1.0010340
C	1.3624120	7.2275350	-0.9884870
C	-1.0608320	7.0028360	-1.1122570
O	-2.1279630	6.3220280	-1.0352720
C	-4.8866640	0.2758090	-2.0545960
C	-4.5971450	1.4051840	-2.8313760
C	-4.7993950	-1.0237280	-2.6309380
O	-5.0118440	-2.0769700	-1.9623820
C	-0.3082970	-4.8849350	-0.9736750
C	-0.2264430	-6.4081840	-1.0010340
C	1.0608320	-7.0028360	-1.1122570
O	2.1279630	-6.3220280	-1.0352720
C	-1.3624120	-7.2275350	-0.9884870
C	4.8866640	-0.2758090	-2.0545960
C	4.7993950	1.0237280	-2.6309380
O	5.0118440	2.0769700	-1.9623820
C	4.5971450	-1.4051840	-2.8313760
H	5.9702560	-1.3028620	-0.5044570
H	6.1198260	0.4351530	-0.4819450
H	-0.5241070	4.5173880	-1.5835130
H	1.2239610	4.5714200	-1.4815980
H	-5.9702560	1.3028620	-0.5044570
H	-6.1198260	-0.4351530	-0.4819450
H	0.5241070	-4.5173880	-1.5835130
H	-1.2239610	-4.5714200	-1.4815980
C	-4.8196430	2.1640270	4.7926210
C	-3.4196450	3.3384680	6.5301570
C	3.3780920	3.5241020	6.4588890
C	2.1279630	4.8464450	4.7136160
C	3.4196450	-3.3384680	6.5301570
C	4.8196430	-2.1640270	4.7926210
C	-3.3780920	-3.5241020	6.4588890
C	-2.1279630	-4.8464450	4.7136160
H	-3.9670710	4.1220330	4.6118290
H	-4.1099580	-4.0563960	4.5159960
H	3.9670710	-4.1220330	4.6118290
H	4.1099580	4.0563960	4.5159960
H	-5.0729020	2.0703550	3.7337250
H	-4.5638190	1.1637330	5.1626440
H	-5.7176890	2.4963440	5.3256230
H	-3.1120120	2.3955490	6.9990390
H	-2.6322510	4.0764380	6.7190060

H	-4.3287270	3.6790400	7.0375630
H	2.4545140	3.1939150	6.9503970
H	4.1464560	2.7672730	6.6513190
H	3.6947590	4.4552220	6.9412200
H	2.0036190	5.0700720	3.6511290
H	1.1444110	4.5680010	5.1111610
H	2.4418960	5.7675780	5.2176240
H	3.1120120	-2.3955490	6.9990390
H	2.6322510	-4.0764380	6.7190060
H	4.3287270	-3.6790400	7.0375630
H	5.0729020	-2.0703550	3.7337250
H	4.5638190	-1.1637330	5.1626440
H	5.7176890	-2.4963440	5.3256230
H	-2.4545140	-3.1939150	6.9503970
H	-4.1464560	-2.7672730	6.6513190
H	-3.6947590	-4.4552220	6.9412200
H	-2.0036190	-5.0700720	3.6511290
H	-1.1444110	-4.5680010	5.1111610
H	-2.4418960	-5.7675780	5.2176240
C	1.2126410	-8.5000020	-1.2903890
H	1.3713470	-8.9311650	-0.2908170
H	2.1355140	-8.6775710	-1.8524350
C	0.0070140	-9.1778790	-1.9598830
C	-1.2605360	-8.7216750	-1.2102480
H	-2.1441880	-9.0739480	-1.7504600
H	-1.2982840	-9.2063500	-0.2232900
C	-3.6231120	-0.0729630	-4.6941320
C	-4.2535140	1.2805500	-4.3012490
H	-3.5724380	2.0833100	-4.5964680
H	-5.1785360	1.4292760	-4.8764400
C	-4.5020250	-1.1903220	-4.1083940
H	-5.4677510	-1.2102520	-4.6346790
H	-4.0451670	-2.1745200	-4.2533150
C	4.2535140	-1.2805500	-4.3012490
H	3.5724380	-2.0833100	-4.5964680
H	5.1785360	-1.4292760	-4.8764400
C	3.6231120	0.0729630	-4.6941320
C	4.5020250	1.1903220	-4.1083940
H	5.4677510	1.2102520	-4.6346790
H	4.0451670	2.1745200	-4.2533150
C	-1.2126410	8.5000020	-1.2903890
H	-1.3713470	8.9311650	-0.2908170
H	-2.1355140	8.6775710	-1.8524350
C	-0.0070140	9.1778790	-1.9598830
C	1.2605360	8.7216750	-1.2102480
H	2.1441880	9.0739480	-1.7504600
H	1.2982840	9.2063500	-0.2232900
C	-2.1848010	-0.1758100	-4.1478320
H	-1.7388910	-1.1361860	-4.4272490
H	-1.5563000	0.6195660	-4.5626320
H	-2.1544770	-0.0954810	-3.0574080
C	-3.5851110	-0.1837350	-6.2273430
H	-2.9796500	0.6186700	-6.6633070
H	-3.1450770	-1.1386890	-6.5338620
H	-4.5905970	-0.1213180	-6.6577000
C	0.0767830	8.7874890	-3.4496890
H	-0.8264430	9.1078220	-3.9797290
H	0.9351510	9.2721350	-3.9273570
H	0.1815150	7.7072710	-3.5846900
C	-0.1361540	10.7064680	-1.8509200
H	0.7268840	11.2063480	-2.3044880
H	-1.0354200	11.0556600	-2.3694750
H	-0.2024830	11.0281770	-0.8058800
C	-0.0767830	-8.7874890	-3.4496890
H	0.8264430	-9.1078220	-3.9797290
H	-0.9351510	-9.2721350	-3.9273570
H	-0.1815150	-7.7072710	-3.5846900
C	0.1361540	-10.7064680	-1.8509200
H	-0.7268840	-11.2063480	-2.3044880
H	1.0354200	-11.0556600	-2.3694750
H	0.2024830	-11.0281770	-0.8058800
C	2.1848010	0.1758100	-4.1478320
H	1.7388910	1.1361860	-4.4272490
H	1.5563000	-0.6195660	-4.5626320
H	2.1544770	0.0954810	-3.0574080
C	3.5851110	0.1837350	-6.2273430

H	2.9796500	-0.6186700	-6.6633070
H	3.1450770	1.1386890	-6.5338620
H	4.5905970	0.1213180	-6.6577000
N	-2.5879190	-6.7313880	-0.7483770
N	-4.6511670	2.6343690	-2.2918520
N	4.6511670	-2.6343690	-2.2918520
N	2.5879190	6.7313880	-0.7483770
H	-2.6670720	-5.7623710	-0.4335130
H	-4.7369440	2.6833310	-1.2753730
H	2.6670720	5.7623710	-0.4335130
H	4.7369440	-2.6833310	-1.2753730
C	4.5114830	-3.9381590	-2.9442060
H	3.5749130	-4.4069540	-2.6209060
H	4.4527170	-3.7969370	-4.0236890
C	-3.8327330	-7.5129890	-0.7417900
H	-4.0070440	-7.9214660	-1.7459040
H	-3.7386890	-8.3660260	-0.0617110
C	-4.5114830	3.9381590	-2.9442060
H	-3.5749130	4.4069540	-2.6209060
H	-4.4527170	3.7969370	-4.0236890
C	3.8327330	7.5129890	-0.7417900
H	4.0070440	7.9214660	-1.7459040
H	3.7386890	8.3660260	-0.0617110
C	5.6770870	-4.8552310	-2.6090130
C	5.4592970	-6.0593910	-1.9295840
C	6.9835230	-4.5085910	-2.9831510
C	6.5323360	-6.9061560	-1.6336800
H	4.4498730	-6.3334040	-1.6320970
C	8.0530520	-5.3520670	-2.6860780
H	7.1623280	-3.5733110	-3.5073330
C	7.8293300	-6.5555980	-2.0096430
H	6.3524250	-7.8382780	-1.1055470
H	9.0599780	-5.0734760	-2.9832500
H	8.6622370	-7.2130560	-1.7780810
C	-5.0246660	-6.6752610	-0.3288210
C	-5.3663510	-5.5069690	-1.0249000
C	-5.8315010	-7.0861340	0.7388610
C	-6.4880030	-4.7637960	-0.6562170
H	-4.7516530	-5.1643250	-1.8525510
C	-6.9627750	-6.3512020	1.1013460
H	-5.5748910	-7.9880880	1.2881400
C	-7.2925130	-5.1876640	0.4049170
H	-6.7190290	-3.8516830	-1.1962240
H	-7.5796130	-6.6854930	1.9303330
H	-8.1682670	-4.6116310	0.6891550
C	-5.6770870	4.8552310	-2.6090130
C	-5.4592970	6.0593910	-1.9295840
C	-6.9835230	4.5085910	-2.9831510
C	-6.5323360	6.9061560	-1.6336800
H	-4.4498730	6.3334040	-1.6320970
C	-8.0530520	5.3520670	-2.6860780
H	-7.1623280	3.5733110	-3.5073330
C	-7.8293300	6.5555980	-2.0096430
H	-6.3524250	7.8382780	-1.1055470
H	-9.0599780	5.0734760	-2.9832500
H	-8.6622370	7.2130560	-1.7780810
C	5.0246660	6.6752610	-0.3288210
C	5.3663510	5.5069690	-1.0249000
C	5.8315010	7.0861340	0.7388610
C	6.4880030	4.7637960	-0.6562170
H	4.7516530	5.1643250	-1.8525510
C	6.9627750	6.3512020	1.1013460
H	5.5748910	7.9880880	1.2881400
C	7.2925130	5.1876640	0.4049170
H	6.7190290	3.8516830	-1.1962240
H	7.5796130	6.6854930	1.9303330
H	8.1682670	4.6116310	0.6891550

C_4 crown-*in* in DMSO
Energy total = -5317.13305223 a.u.

Symbol	X	Y	Z
C	-0.6603120	-3.1893160	2.5675030
C	-1.1554670	-3.8975210	1.4553820

C	-2.4285280	-3.6324330	0.9162370
C	-3.1700760	-2.5682520	1.4605400
C	-2.6999680	-1.8176190	2.5519110
C	-1.4463740	-2.1534260	3.0725260
O	-0.3713600	-4.8966900	0.9271150
O	-4.4158310	-2.3283650	0.9204340
H	-1.0788960	-1.5907430	3.9240610
C	-3.5523230	-0.7027470	3.1684330
C	-3.1893160	0.6603120	2.5675030
C	-3.8975210	1.1554670	1.4553820
C	-3.6324330	2.4285280	0.9162370
C	-2.5682520	3.1700760	1.4605400
C	-1.8176190	2.6999680	2.5519110
C	-2.1534260	1.4463740	3.0725260
O	-4.8966900	0.3713600	0.9271150
H	-1.5907430	1.0788960	3.9240610
C	-0.7027470	3.5523230	3.1684330
C	0.6603120	3.1893160	2.5675030
C	1.1554670	3.8975210	1.4553820
C	2.4285280	3.6324330	0.9162370
C	3.1700760	2.5682520	1.4605400
C	2.6999680	1.8176190	2.5519110
C	1.4463740	2.1534260	3.0725260
O	0.3713600	4.8966900	0.9271150
O	4.4158310	2.3283650	0.9204340
H	1.0788960	1.5907430	3.9240610
C	3.5523230	0.7027470	3.1684330
C	3.1893160	-0.6603120	2.5675030
C	3.8975210	-1.1554670	1.4553820
C	3.6324330	-2.4285280	0.9162370
C	2.5682520	-3.1700760	1.4605400
C	1.8176190	-2.6999680	2.5519110
C	2.1534260	-1.4463740	3.0725260
O	4.8966900	-0.3713600	0.9271150
O	2.3283650	-4.4158310	0.9204340
H	1.5907430	-1.0788960	3.9240610
C	0.7027470	-3.5523230	3.1684330
C	-3.5460420	-0.7548030	4.7211060
C	-0.7548030	3.5460420	4.7211060
H	-0.9068530	4.5873420	2.8799170
C	3.5460420	0.7548030	4.7211060
H	4.5873420	0.9068530	2.8799170
C	0.7548030	-3.5460420	4.7211060
H	0.9068530	-4.5873420	2.8799170
H	-3.4290100	-1.8056600	5.0099780
H	-2.6743810	-0.2334010	5.1355270
C	-4.8236450	-0.2300670	5.4119810
H	-1.8056600	3.4290100	5.0099780
H	-0.2334010	2.6743810	5.1355270
C	-0.2300670	4.8236450	5.4119810
H	3.4290100	1.8056600	5.0099780
H	2.6743810	0.2334010	5.1355270
C	4.8236450	0.2300670	5.4119810
H	1.8056600	-3.4290100	5.0099780
H	0.2334010	-2.6743810	5.1355270
C	0.2300670	-4.8236450	5.4119810
H	-4.5873420	-0.9068530	2.8799170
C	4.5460350	-3.0367690	-0.1439870
H	-5.0255960	0.5746830	-0.0613200
H	0.5746830	5.0255960	-0.0613200
H	4.6281150	1.3648580	0.9721160
H	5.0255960	-0.5746830	-0.0613200
H	1.3648580	-4.6281150	0.9721160
H	-0.5746830	-5.0255960	-0.0613200
O	-2.3283650	4.4158310	0.9204340
C	-3.0367690	-4.5460350	-0.1439870
H	-4.6281150	-1.3648580	0.9721160
C	-4.5460350	3.0367690	-0.1439870
H	-1.3648580	4.6281150	0.9721160
C	-2.8515780	-4.2055270	-1.6192130
C	-3.8584110	-3.6004530	-2.3836440
C	-1.6419260	-4.6113840	-2.2450240
O	-0.6863210	-5.1382190	-1.6004950
C	-4.2055270	2.8515780	-1.6192130
C	-3.6004530	3.8584110	-2.3836440
C	-4.6113840	1.6419260	-2.2450240

O	-5.1382190	0.6863210	-1.6004950
C	3.0367690	4.5460350	-0.1439870
C	2.8515780	4.2055270	-1.6192130
C	1.6419260	4.6113840	-2.2450240
O	0.6863210	5.1382190	-1.6004950
C	3.8584110	3.6004530	-2.3836440
C	4.2055270	-2.8515780	-1.6192130
C	4.6113840	-1.6419260	-2.2450240
O	5.1382190	-0.6863210	-1.6004950
C	3.6004530	-3.8584110	-2.3836440
H	5.5443400	-2.6122280	0.0063000
H	4.6455210	-4.1049330	0.0680860
H	-2.6122280	-5.5443400	0.0063000
H	-4.1049330	-4.6455210	0.0680860
H	-5.5443400	2.6122280	0.0063000
H	-4.6455210	4.1049330	0.0680860
H	2.6122280	5.5443400	0.0063000
H	4.1049330	4.6455210	0.0680860
C	-5.0854900	1.2681970	5.2010860
C	-4.7741520	-0.5554020	6.9133040
C	0.5554020	-4.7741520	6.9133040
C	-1.2681970	-5.0854900	5.2010860
C	4.7741520	0.5554020	6.9133040
C	5.0854900	-1.2681970	5.2010860
C	-0.5554020	4.7741520	6.9133040
C	1.2681970	5.0854900	5.2010860
H	-5.6731740	-0.7814880	4.9808870
H	-0.7814880	5.6731740	4.9808870
H	5.6731740	0.7814880	4.9808870
H	0.7814880	-5.6731740	4.9808870
H	-5.2054510	1.5209000	4.1448290
H	-4.2569720	1.8697650	5.5941720
H	-5.9971880	1.5742480	5.7266410
H	-3.9398570	-0.0345320	7.3993750
H	-4.6431860	-1.6290760	7.0873510
H	-5.6963320	-0.2427530	7.4150180
H	0.0345320	-3.9398570	7.3993750
H	1.6290760	-4.6431860	7.0873510
H	0.2427530	-5.6963320	7.4150180
H	-1.5209000	-5.2054510	4.1448290
H	-1.8697650	-4.2569720	5.5941720
H	-1.5742480	-5.9971880	5.7266410
H	3.9398570	0.0345320	7.3993750
H	4.6431860	1.6290760	7.0873510
H	5.6963320	0.2427530	7.4150180
H	5.2054510	-1.5209000	4.1448290
H	4.2569720	-1.8697650	5.5941720
H	5.9971880	-1.5742480	5.7266410
H	-0.0345320	3.9398570	7.3993750
H	-1.6290760	4.6431860	7.0873510
H	-0.2427530	5.6963320	7.4150180
H	1.5209000	5.2054510	4.1448290
H	1.8697650	4.2569720	5.5941720
H	1.5742480	5.9971880	5.7266410
C	1.4329940	4.3938270	-3.7310090
H	0.7334390	5.1571750	-4.0866830
H	0.9266280	3.4247930	-3.8522680
C	-4.3938270	1.4329940	-3.7310090
H	-5.1571750	0.7334390	-4.0866830
H	-3.4247930	0.9266280	-3.8522680
C	-1.4329940	-4.3938270	-3.7310090
H	-0.7334390	-5.1571750	-4.0866830
H	-0.9266280	-3.4247930	-3.8522680
C	4.3938270	-1.4329940	-3.7310090
H	5.1571750	-0.7334390	-4.0866830
H	3.4247930	-0.9266280	-3.8522680
C	4.4047540	-2.7324200	-4.5527890
C	3.4333920	-3.7227400	-3.8805390
H	2.3959210	-3.4065660	-4.0644560
H	3.5338180	-4.7026590	-4.3558850
C	-2.7324200	-4.4047540	-4.5527890
C	-3.7227400	-3.4333920	-3.8805390
H	-3.4065660	-2.3959210	-4.0644560
H	-4.7026590	-3.5338180	-4.3558850
C	-4.4047540	2.7324200	-4.5527890
C	-3.4333920	3.7227400	-3.8805390

H	-2.3959210	3.4065660	-4.0644560
H	-3.5338180	4.7026590	-4.3558850
C	2.7324200	4.4047540	-4.5527890
C	3.7227400	3.4333920	-3.8805390
H	3.4065660	2.3959210	-4.0644560
H	4.7026590	3.5338180	-4.3558850
C	3.3260830	5.8272620	-4.6053400
H	4.2503640	5.8379730	-5.1931510
H	2.6205570	6.5189000	-5.0779450
H	3.5571180	6.2114860	-3.6075780
C	2.4500240	3.9271510	-5.9869890
H	2.0192130	2.9199860	-5.9940030
H	1.7445810	4.5996840	-6.4866240
H	3.3699280	3.9056000	-6.5817200
C	5.8272620	-3.3260830	-4.6053400
H	5.8379730	-4.2503640	-5.1931510
H	6.5189000	-2.6205570	-5.0779450
H	6.2114860	-3.5571180	-3.6075780
C	3.9271510	-2.4500240	-5.9869890
H	2.9199860	-2.0192130	-5.9940030
H	4.5996840	-1.7445810	-6.4866240
H	3.9056000	-3.3699280	-6.5817200
C	-3.3260830	-5.8272620	-4.6053400
H	-4.2503640	-5.8379730	-5.1931510
H	-2.6205570	-6.5189000	-5.0779450
H	-3.5571180	-6.2114860	-3.6075780
C	-2.4500240	-3.9271510	-5.9869890
H	-2.0192130	-2.9199860	-5.9940030
H	-1.7445810	-4.5996840	-6.4866240
H	-3.3699280	-3.9056000	-6.5817200
C	-5.8272620	3.3260830	-4.6053400
H	-5.8379730	4.2503640	-5.1931510
H	-6.5189000	2.6205570	-5.0779450
H	-6.2114860	3.5571180	-3.6075780
C	-3.9271510	2.4500240	-5.9869890
H	-2.9199860	2.0192130	-5.9940030
H	-4.5996840	1.7445810	-6.4866240
H	-3.9056000	3.3699280	-6.5817200
N	-4.9699330	-3.1266950	-1.7960500
N	3.1266950	-4.9699330	-1.7960500
N	4.9699330	3.1266950	-1.7960500
N	-3.1266950	4.9699330	-1.7960500
H	-2.9984740	4.9204670	-0.7846820
H	-4.9204670	-2.9984740	-0.7846820
H	2.9984740	-4.9204670	-0.7846820
H	4.9204670	2.9984740	-0.7846820
C	6.1037370	2.4428260	-2.4323850
H	5.8510150	1.3914930	-2.6071030
H	6.3123510	2.9125650	-3.3963240
C	-2.4428260	6.1037370	-2.4323850
H	-1.3914930	5.8510150	-2.6071030
H	-2.9125650	6.3123510	-3.3963240
C	-6.1037370	-2.4428260	-2.4323850
H	-5.8510150	-1.3914930	-2.6071030
H	-6.3123510	-2.9125650	-3.3963240
C	2.4428260	-6.1037370	-2.4323850
H	1.3914930	-5.8510150	-2.6071030
H	2.9125650	-6.3123510	-3.3963240
C	7.3343880	2.5332950	-1.5512410
C	7.8463960	1.3891280	-0.9282800
C	7.9759580	3.7639470	-1.3493020
C	8.9846670	1.4718510	-0.1210070
H	7.3471880	0.4361910	-1.0779860
C	9.1097030	3.8481170	-0.5418980
H	7.5841950	4.6575760	-1.8283750
C	9.6179530	2.6999680	0.0737590
H	9.3737570	0.5769200	0.3559560
H	9.5996590	4.8063860	-0.3957310
H	10.5023380	2.7648030	0.7008150
C	-2.5332950	7.3343880	-1.5512410
C	-1.3891280	7.8463960	-0.9282800
C	-3.7639470	7.9759580	-1.3493020
C	-1.4718510	8.9846670	-0.1210070
H	-0.4361910	7.3471880	-1.0779860
C	-3.8481170	9.1097030	-0.5418980
H	-4.6575760	7.5841950	-1.8283750

C	-2.6999680	9.6179530	0.0737590
H	-0.5769200	9.3737570	0.3559560
H	-4.8063860	9.5996590	-0.3957310
H	-2.7648030	10.5023380	0.7008150
C	-7.3343880	-2.5332950	-1.5512410
C	-7.8463960	-1.3891280	-0.9282800
C	-7.9759580	-3.7639470	-1.3493020
C	-8.9846670	-1.4718510	-0.1210070
H	-7.3471880	-0.4361910	-1.0779860
C	-9.1097030	-3.8481170	-0.5418980
H	-7.5841950	-4.6575760	-1.8283750
C	-9.6179530	-2.6999680	0.0737590
H	-9.3737570	-0.5769200	0.3559560
H	-9.5996590	-4.8063860	-0.3957310
H	-10.5023380	-2.7648030	0.7008150
C	2.5332950	-7.3343880	-1.5512410
C	1.3891280	-7.8463960	-0.9282800
C	3.7639470	-7.9759580	-1.3493020
C	1.4718510	-8.9846670	-0.1210070
H	0.4361910	-7.3471880	-1.0779860
C	3.8481170	-9.1097030	-0.5418980
H	4.6575760	-7.5841950	-1.8283750
C	2.6999680	-9.6179530	0.0737590
H	0.5769200	-9.3737570	0.3559560
H	4.8063860	-9.5996590	-0.3957310
H	2.7648030	-10.5023380	0.7008150

C_4 crown-*out* in DMSO
Energy total = -5317.13032292 a.u.

Symbol	X	Y	Z
C	1.4287190	2.9294330	1.7741820
C	1.4545000	3.7753670	0.6484730
C	0.2792420	4.3081400	0.0884680
C	-0.9528750	3.9510760	0.6644860
C	-1.0261200	3.0906440	1.7750170
C	0.1728250	2.5935610	2.2901040
O	2.6396830	4.1437850	0.0521940
O	-2.1332880	4.3928190	0.1263610
H	0.1252070	1.9328410	3.1496980
C	-2.3877590	2.7305940	2.3800210
C	-2.9294330	1.4287190	1.7741820
C	-3.7753670	1.4545000	0.6484730
C	-4.3081400	0.2792420	0.0884680
C	-3.9510760	-0.9528750	0.6644860
C	-3.0906440	-1.0261200	1.7750170
C	-2.5935610	0.1728250	2.2901040
O	-4.1437850	2.6396830	0.0521940
H	-1.9328410	0.1252070	3.1496980
C	-2.7305940	-2.3877590	2.3800210
C	-1.4287190	-2.9294330	1.7741820
C	-1.4545000	-3.7753670	0.6484730
C	-0.2792420	-4.3081400	0.0884680
C	0.9528750	-3.9510760	0.6644860
C	1.0261200	-3.0906440	1.7750170
C	-0.1728250	-2.5935610	2.2901040
O	-2.6396830	-4.1437850	0.0521940
O	2.1332880	-4.3928190	0.1263610
H	-0.1252070	-1.9328410	3.1496980
C	2.3877590	-2.7305940	2.3800210
C	2.9294330	-1.4287190	1.7741820
C	3.7753670	-1.4545000	0.6484730
C	4.3081400	-0.2792420	0.0884680
C	3.9510760	0.9528750	0.6644860
C	3.0906440	1.0261200	1.7750170
C	2.5935610	-0.1728250	2.2901040
O	4.1437850	-2.6396830	0.0521940
O	4.3928190	2.1332880	0.1263610
H	1.9328410	-0.1252070	3.1496980
C	2.7305940	2.3877590	2.3800210
C	-2.3693870	2.7690670	3.9325280
C	-2.7690670	-2.3693870	3.9325280
H	-3.5280040	-3.0716020	2.0744960
C	2.3693870	-2.7690670	3.9325280

H	3.0716020	-3.5280040	2.0744960
C	2.7690670	2.3693870	3.9325280
H	3.5280040	3.0716020	2.0744960
H	-1.6261250	3.5172910	4.2312270
H	-2.0189500	1.8183150	4.3525430
C	-3.7038540	3.1562380	4.6056660
H	-3.5172910	-1.6261250	4.2312270
H	-1.8183150	-2.0189500	4.3525430
C	-3.1562380	-3.7038540	4.6056660
H	1.6261250	-3.5172910	4.2312270
H	2.0189500	-1.8183150	4.3525430
C	3.7038540	-3.1562380	4.6056660
H	3.5172910	1.6261250	4.2312270
H	1.8183150	2.0189500	4.3525430
C	3.1562380	3.7038540	4.6056660
H	-3.0716020	3.5280040	2.0744960
C	5.1900820	-0.3347170	-1.1516650
H	-3.4201230	3.3143110	0.0836880
H	-3.3143110	-3.4201230	0.0836880
H	2.0567290	-5.2838960	-0.3646920
H	3.4201230	-3.3143110	0.0836880
H	5.2838960	2.0567290	-0.3646920
H	3.3143110	3.4201230	0.0836880
O	-4.3928190	-2.1332880	0.1263610
C	0.3347170	5.1900820	-1.1516650
H	-2.0567290	5.2838960	-0.3646920
C	-5.1900820	0.3347170	-1.1516650
H	-5.2838960	-2.0567290	-0.3646920
C	0.2971710	6.7030190	-0.9480840
C	1.4545000	7.4740060	-0.8134830
C	-0.9767740	7.3433480	-0.9245140
O	-2.0638010	6.6981780	-0.9365210
C	-6.7030190	0.2971710	-0.9480840
C	-7.4740060	1.4545000	-0.8134830
C	-7.3433480	-0.9767740	-0.9245140
O	-6.6981780	-2.0638010	-0.9365210
C	-0.3347170	-5.1900820	-1.1516650
C	-0.2971710	-6.7030190	-0.9480840
C	0.9767740	-7.3433480	-0.9245140
O	2.0638010	-6.6981780	-0.9365210
C	-1.4545000	-7.4740060	-0.8134830
C	6.7030190	-0.2971710	-0.9480840
C	7.3433480	0.9767740	-0.9245140
O	6.6981780	2.0638010	-0.9365210
C	7.4740060	-1.4545000	-0.8134830
H	4.9347380	0.5195370	-1.7872180
H	4.9242200	-1.2308690	-1.7187350
H	-0.5195370	4.9347380	-1.7872180
H	1.2308690	4.9242200	-1.7187350
H	-4.9347380	-0.5195370	-1.7872180
H	-4.9242200	1.2308690	-1.7187350
H	0.5195370	-4.9347380	-1.7872180
H	-1.2308690	-4.9242200	-1.7187350
C	-4.8465520	2.1611970	4.3572230
C	-3.4878360	3.3555170	6.1140320
C	3.3555170	3.4878360	6.1140320
C	2.1611970	4.8465520	4.3572230
C	3.4878360	-3.3555170	6.1140320
C	4.8465520	-2.1611970	4.3572230
C	-3.3555170	-3.4878360	6.1140320
C	-2.1611970	-4.8465520	4.3572230
H	-4.0086690	4.1258440	4.1829530
H	-4.1258440	-4.0086690	4.1829530
H	4.0086690	-4.1258440	4.1829530
H	4.1258440	4.0086690	4.1829530
H	-5.0772530	2.0574630	3.2940060
H	-4.5903050	1.1657310	4.7396920
H	-5.7579860	2.4898780	4.8693570
H	-3.1778450	2.4179140	6.5919330
H	-2.7119990	4.1029000	6.3133140
H	-4.4091820	3.6879920	6.6044270
H	2.4179140	3.1778450	6.5919330
H	4.1029000	2.7119990	6.3133140
H	3.6879920	4.4091820	6.6044270
H	2.0574630	5.0772530	3.2940060
H	1.1657310	4.5903050	4.7396920

H	2.4898780	5.7579860	4.8693570
H	3.1778450	-2.4179140	6.5919330
H	2.7119990	-4.1029000	6.3133140
H	4.4091820	-3.6879920	6.6044270
H	5.0772530	-2.0574630	3.2940060
H	4.5903050	-1.1657310	4.7396920
H	5.7579860	-2.4898780	4.8693570
H	-2.4179140	-3.1778450	6.5919330
H	-4.1029000	-2.7119990	6.3133140
H	-3.6879920	-4.4091820	6.6044270
H	-2.0574630	-5.0772530	3.2940060
H	-1.1657310	-4.5903050	4.7396920
H	-2.4898780	-5.7579860	4.8693570
C	1.0777990	-8.8546120	-0.8410570
H	1.2136290	-9.1080960	0.2208780
H	2.0002300	-9.1546920	-1.3494890
C	-0.1437590	-9.6047340	-1.3958360
C	-1.4008420	-8.9849500	-0.7526970
H	-2.2947450	-9.4016290	-1.2256880
H	-1.4502470	-9.2767620	0.3068410
C	-9.6047340	0.1437590	-1.3958360
C	-8.9849500	1.4008420	-0.7526970
H	-9.4016290	2.2947450	-1.2256880
H	-9.2767620	1.4502470	0.3068410
C	-8.8546120	-1.0777990	-0.8410570
H	-9.1080960	-1.2136290	0.2208780
H	-9.1546920	-2.0002300	-1.3494890
C	8.9849500	-1.4008420	-0.7526970
H	9.4016290	-2.2947450	-1.2256880
H	9.2767620	-1.4502470	0.3068410
C	9.6047340	-0.1437590	-1.3958360
C	8.8546120	1.0777990	-0.8410570
H	9.1080960	1.2136290	0.2208780
H	9.1546920	2.0002300	-1.3494890
C	-1.0777990	8.8546120	-0.8410570
H	-1.2136290	9.1080960	0.2208780
H	-2.0002300	9.1546920	-1.3494890
C	0.1437590	9.6047340	-1.3958360
C	1.4008420	8.9849500	-0.7526970
H	2.2947450	9.4016290	-1.2256880
H	1.4502470	9.2767620	0.3068410
C	-9.4829000	0.2051540	-2.9319730
H	-9.9219550	-0.6897050	-3.3857440
H	-10.0154470	1.0772000	-3.3264420
H	-8.4409940	0.2706200	-3.2576850
C	-11.0927230	0.0628170	-1.0158450
H	-11.6401150	0.9368980	-1.3856830
H	-11.5545070	-0.8297330	-1.4513420
H	-11.2250690	0.0152210	0.0705790
C	0.2051540	9.4829000	-2.9319730
H	-0.6897050	9.9219550	-3.3857440
H	1.0772000	10.0154470	-3.3264420
H	0.2706200	8.4409940	-3.2576850
C	0.0628170	11.0927230	-1.0158450
H	0.9368980	11.6401150	-1.3856830
H	-0.8297330	11.5545070	-1.4513420
H	0.0152210	11.2250690	0.0705790
C	-0.2051540	-9.4829000	-2.9319730
H	0.6897050	-9.9219550	-3.3857440
H	-1.0772000	-10.0154470	-3.3264420
H	-0.2706200	-8.4409940	-3.2576850
C	-0.0628170	-11.0927230	-1.0158450
H	-0.9368980	-11.6401150	-1.3856830
H	0.8297330	-11.5545070	-1.4513420
H	-0.0152210	-11.2250690	0.0705790
C	9.4829000	-0.2051540	-2.9319730
H	9.9219550	0.6897050	-3.3857440
H	10.0154470	-1.0772000	-3.3264420
H	8.4409940	-0.2706200	-3.2576850
C	11.0927230	-0.0628170	-1.0158450
H	11.6401150	-0.9368980	-1.3856830
H	11.5545070	0.8297330	-1.4513420
H	11.2250690	-0.0152210	0.0705790
N	-2.6654290	-6.8972670	-0.6966110
N	-6.8972670	2.6654290	-0.6966110
N	6.8972670	-2.6654290	-0.6966110

N	2.6654290	6.8972670	-0.6966110
H	-2.6996620	-5.8851120	-0.5738610
H	-5.8851120	2.6996620	-0.5738610
H	2.6996620	5.8851120	-0.5738610
H	5.8851120	-2.6996620	-0.5738610
C	7.5844920	-3.9435900	-0.5327500
H	8.3977900	-3.8523260	0.1938260
H	6.8497890	-4.6189900	-0.0814310
C	-3.9435900	-7.5844920	-0.5327500
H	-3.8523260	-8.3977900	0.1938260
H	-4.6189900	-6.8497890	-0.0814310
C	-7.5844920	3.9435900	-0.5327500
H	-8.3977900	3.8523260	0.1938260
H	-6.8497890	4.6189900	-0.0814310
C	3.9435900	7.5844920	-0.5327500
H	3.8523260	8.3977900	0.1938260
H	4.6189900	6.8497890	-0.0814310
C	8.1144440	-4.5669400	-1.8184490
C	9.2293540	-5.4135290	-1.7640160
C	7.4851610	-4.3553050	-3.0509790
C	9.7028340	-6.0425330	-2.9168320
H	9.7305570	-5.5808420	-0.8137010
C	7.9599220	-4.9803810	-4.2056860
H	6.6249900	-3.6949420	-3.1066820
C	9.0687220	-5.8273560	-4.1424040
H	10.5702720	-6.6934950	-2.8581340
H	7.4624760	-4.8052290	-5.1552460
H	9.4381660	-6.3119310	-5.0412600
C	-4.5669400	-8.1144440	-1.8184490
C	-5.4135290	-9.2293540	-1.7640160
C	-4.3553050	-7.4851610	-3.0509790
C	-6.0425330	-9.7028340	-2.9168320
H	-5.5808420	-9.7305570	-0.8137010
C	-4.9803810	-7.9599220	-4.2056860
H	-3.6949420	-6.6249900	-3.1066820
C	-5.8273560	-9.0687220	-4.1424040
H	-6.6934950	-10.5702720	-2.8581340
H	-4.8052290	-7.4624760	-5.1552460
H	-6.3119310	-9.4381660	-5.0412600
C	-8.1144440	4.5669400	-1.8184490
C	-9.2293540	5.4135290	-1.7640160
C	-7.4851610	4.3553050	-3.0509790
C	-9.7028340	6.0425330	-2.9168320
H	-9.7305570	5.5808420	-0.8137010
C	-7.9599220	4.9803810	-4.2056860
H	-6.6249900	3.6949420	-3.1066820
C	-9.0687220	5.8273560	-4.1424040
H	-10.5702720	6.6934950	-2.8581340
H	-7.4624760	4.8052290	-5.1552460
H	-9.4381660	6.3119310	-5.0412600
C	4.5669400	8.1144440	-1.8184490
C	5.4135290	9.2293540	-1.7640160
C	4.3553050	7.4851610	-3.0509790
C	6.0425330	9.7028340	-2.9168320
H	5.5808420	9.7305570	-0.8137010
C	4.9803810	7.9599220	-4.2056860
H	3.6949420	6.6249900	-3.1066820
C	5.8273560	9.0687220	-4.1424040
H	6.6934950	10.5702720	-2.8581340
H	4.8052290	7.4624760	-5.1552460
H	6.3119310	9.4381660	-5.0412600

9. DFTB/GFN2-xTB calculations

Atomic coordinates for all calculated geometries:

C_2 in chloroform
Energy total = -359.525518333002 a.u.

Symbol	X	Y	Z
C	-1.39233440939539	-2.79621267988148	1.77205756126630
C	-1.40190189195070	-3.52889551534007	0.58368343005384
C	-0.21937296725526	-4.02270070520330	0.02338732597574
C	0.99253233702365	-3.74786181458977	0.65360461090755
C	1.03071321938180	-2.97206973876341	1.81743878952324
C	-0.16069290059559	-2.49958479042019	2.33564072207264
O	-2.56203105836668	-3.82488248436475	-0.08986763343585
O	2.18722096083988	-4.19890220573781	0.16356331882558
H	-0.12416798642093	-1.90087447998348	3.22786434666835
C	2.36790564628323	-2.69028918251522	2.47740101834871
C	2.95847231487840	-1.42391396336866	1.88486751921884
C	3.95870845969139	-1.47969028086596	0.91154639453439
C	4.54329937431824	-0.31948151523501	0.39619918089733
C	4.10742942530686	0.91764660478429	0.86926813485983
C	3.10234684135193	0.99704677609653	1.83881361430949
C	2.53096579253205	-0.17314838343817	2.30146321191118
O	4.43790822104035	-2.68213291050532	0.44173404728675
H	1.73729248133570	-0.10239127629611	3.02458637627207
C	2.70133338832501	2.35134813303880	2.39480107388988
C	1.39233427611225	2.79621264133396	1.77205757417079
C	1.40190168925219	3.52889560679196	0.58368354277525
C	0.21937267674166	4.02270092535784	0.02338768665334
C	-0.99253256661789	3.74786194949933	0.65360504049472
C	-1.03071340536906	2.97206972889442	1.81743910360685
C	0.16069281186938	2.49958476745346	2.33564088672304
O	2.56203073541237	3.82488249458854	-0.08986772907555
O	-2.18722127047680	4.19890251390836	0.16356386236737
H	0.12416793162961	1.90087418751633	3.22786433077673
C	-2.36790578121014	2.69028906775005	2.47740135945692
C	-2.95847226134812	1.42391378824536	1.88486781266582
C	-3.95870850737067	1.47969010613426	0.91154683051936
C	-4.54329936790956	0.31948136093621	0.39619950428079
C	-4.10742945627565	-0.91764677027824	0.86926847129545
C	-3.10234686803693	-0.99704696614642	1.83881394127558
C	-2.53096569990273	0.17314818459985	2.30146344206574
O	-4.43790852181031	2.68213280969804	0.44173485247692
O	-4.63568212934124	-2.09988745192320	0.42850225956685
H	-1.73729244497942	0.10239101693000	3.02458664378717
C	-2.70133339404904	-2.35134830312041	2.39480140188024
C	2.27017371003650	-2.67303042567003	4.01218239266834
C	2.73018352686613	2.34117861652455	3.93297194327296
H	3.48070136085489	3.06196988824904	2.09585690899360
C	-2.27017343173099	2.67303024725303	4.01218275274255
H	-3.02426833518725	3.53207438512860	2.23140945314609
C	-2.73018340348209	-2.34117866644768	3.93297228517364
H	-3.48070136790840	-3.06196998321160	2.09585708833282
H	1.53160672297305	-3.42577520576233	4.29717184293992
H	1.92406354290985	-1.70561937031887	4.37210609337446
C	3.58832795570937	-3.01193834205859	4.71954252446629
H	3.55855810120539	1.69236920932648	4.22798261415148
H	1.81775153328603	1.91731636149313	4.34782015557100
C	2.96726935334124	3.72453297913033	4.55052821212095
H	-1.53160645219971	3.42577505201783	4.29717222402730
H	-1.92406327757494	1.70561914427948	4.37210640554491
C	-3.58832768436343	3.01193818544145	4.71954287585243
H	-3.55855802713151	-1.69236927072550	4.22798298617216
H	-1.81775129366906	-1.91731643964742	4.34782040276518
C	-2.96726923647346	-3.72453323954466	4.55052815405165
H	3.02426818659731	-3.53207450024024	2.23140916738334
C	-5.67702836868773	0.42026698015024	-0.60195594639338
H	3.71677651816646	-3.34135307531433	0.47854386037162
H	3.30275721085819	3.25200732898925	0.19610443060520
H	-2.08071544048170	4.82001415423376	-0.60458890065190

H	-3.71677691903564	3.34135300009799	0.47854476807812
H	-5.20949083392764	-1.98965579261473	-0.37877441345153
H	-3.30275745407893	-3.25200721609152	0.19610454305633
O	4.63568217443433	2.09988730295773	0.42850198492041
C	-0.27458472097015	-4.80645936576091	-1.26969333402123
H	2.08071533089023	-4.82001392092087	-0.60458959728373
C	5.67702839483563	-0.42026697630775	-0.60195626760134
H	5.20949076785907	1.98965564123315	-0.37877480497769
C	-0.06565509163845	-6.30315520394108	-1.12029265731343
C	-1.07752024329908	-7.14960740937204	-0.69673547061553
C	1.21424444444705	-6.80709516467623	-1.44472404067519
O	2.17790942748484	-6.04496620374678	-1.71215064446643
C	5.28521392513466	-0.18978934550275	-2.05252916533415
C	4.82119961790336	-1.21412305466312	-2.86293056228056
C	5.50489738805968	1.11017861339365	-2.56988568442668
O	5.91529327860804	2.05585333635404	-1.85506082865953
C	0.27458436565872	4.80645970353414	-1.26969287933565
C	0.06565478000086	6.30315553478935	-1.12029233180280
C	-1.21424420562373	6.80709578015595	-1.44472490312075
O	-2.17790976846254	6.04496691602630	-1.71215056437392
C	1.07752011575561	7.14960765384038	-0.69673500144155
C	-5.28521394623491	0.18978946834987	-2.05252898239217
C	-5.50489803653723	-1.11017872572962	-2.56988546015475
O	-5.91529329368431	-2.05585330990168	-1.85506088797002
C	-4.82119929482079	1.21412281160891	-2.86293022336659
H	-6.15008706773209	1.39774506464719	-0.49158367078538
H	-6.43270977731243	-0.33110397729507	-0.34907376494867
H	0.50406056207297	-4.43469374187605	-1.94409831771396
H	-1.23689504400652	-4.61240660672219	-1.74693287750697
H	6.15008711537841	-1.39774503928615	-0.49158388159269
H	6.43270977446909	0.33110400962609	-0.34907367847517
H	-0.50406088641497	4.43469408367459	-1.94409790657766
H	1.23689463310848	4.61240713385702	-1.74693250376392
C	4.63027591975153	-1.90789404203923	4.55793596586612
C	3.32179906008556	-3.26004869099519	6.20518119261464
C	-3.31815901426737	-3.57444540222325	6.03145272963834
C	-1.75850608315949	-4.64338008954200	4.39460988757769
C	-3.32179877721194	3.26004837988107	6.20518156874355
C	-4.63027577116597	1.90789396099395	4.55793650717399
C	3.31815935020902	3.57444526622292	6.03145273973609
C	1.75850631466330	4.64337992285382	4.39460976494073
H	3.99018835034795	-3.93474169411272	4.28407456806609
H	3.81964434424413	4.18434582432830	4.03755077547611
H	-3.99018813584706	3.93474155404824	4.28407498478017
H	-3.81964427473576	-4.18434612036066	4.03755076706192
H	4.89087753422326	-1.76370943190352	3.51321657609387
H	4.24654050068630	-0.96630373864255	4.94473251670083
H	5.53638283531621	-2.16130721319388	5.10479763817811
H	2.91523325311023	-2.36463582366480	6.67108344138553
H	2.61088277665315	-4.07257951358264	6.33974742479776
H	4.24360540835197	-3.52377187603561	6.71890846950380
H	-2.49908392901299	-3.10086332962205	6.56932062309191
H	-4.21004899897429	-2.96395488722487	6.15653002362975
H	-3.50387460617686	-4.54777204223515	6.48029665851157
H	-1.51828478499160	-4.79717784911904	3.34641220696112
H	-0.88898656136999	-4.20978129066630	4.88374207987977
H	-1.96014326810368	-5.61044230996149	4.85055645715598
H	-2.91523293419220	2.36463552449013	6.67108383506199
H	-2.61088250080105	4.07257920789105	6.33974773685006
H	-4.24360507930174	3.52377160189743	6.71890891220992
H	-4.89087744287229	1.76370936063445	3.51321715085795
H	-4.24654039458104	0.96630362240700	4.94473297043779
H	-5.53638267779556	2.16130707773197	5.10479821547303
H	2.49908423034814	3.10086318193786	6.56932053203883
H	4.21004927567199	2.96395465129918	6.15652999860513
H	3.50387495444897	4.54777189589851	6.48029673707046
H	1.51828496880904	4.79717776988098	3.34641209903545
H	0.88898682383479	4.20978113371491	4.88374196513544
H	1.96014353208186	5.61044207991233	4.85055642684553
C	-1.45552512508211	8.29607365770913	-1.42534538514051
H	-1.88392634426849	8.53334251307246	-0.44534984905699
H	-2.21793280714879	8.52733399052858	-2.17275152858492
C	-0.18741443800224	9.11668357400816	-1.64910221755108
C	0.86530953285534	8.63182487664077	-0.64491404732735
H	1.80734671195940	9.14513778560074	-0.83747191202156
H	0.54447943604226	8.89585648159371	0.36797716824748

C	4.17355822731949	0.48782655929446	-4.62259453407483
C	4.52017802851140	-0.97337168823281	-4.31379272964434
H	3.68383318578774	-1.60257540234081	-4.62091533515031
H	5.39570539083003	-1.26230857994625	-4.90337229536913
C	5.28462934300910	1.35974059929567	-4.04220598152778
H	6.22938819717127	1.14554110658168	-4.55242626579859
H	5.07303082350662	2.42227988201150	-4.18012180423094
C	-4.52017784903493	0.97337157077878	-4.31379253540711
H	-3.68383304144917	1.60257542261035	-4.62091516400329
H	-5.39570533560851	1.26230844320230	-4.90337202924954
C	-4.17355840842501	-0.48782675681962	-4.62259455844611
C	-5.28462946258537	-1.35974067467300	-4.04220583593919
H	-6.22938835933077	-1.14554140542781	-4.55242598422198
H	-5.07303079557339	-2.42227986018356	-4.18012166648324
C	1.45552515990749	-8.29607316297399	-1.42534573579581
H	1.88392674465883	-8.53334204601890	-0.44535041950809
H	2.21793261340903	-8.52733345192800	-2.17275218886394
C	0.18741463704550	-9.11668334375281	-1.64910223543800
C	-0.86530935136922	-8.63182453667512	-0.64491414015886
H	-1.80734639792701	-9.14513767103568	-0.83747187144244
H	-0.54447903726428	-8.89585598737238	0.36797704811726
C	2.82603683163593	0.85537339708699	-3.99653435619283
H	2.58680584483229	1.89650060582900	-4.20021883769937
H	2.03283191219397	0.23455485326189	-4.40701561823533
H	2.84531443583941	0.71664075833617	-2.91779962875971
C	4.09525312837977	0.68241321102672	-6.13875590559902
H	3.32356138474776	0.04499136692446	-6.56436414641286
H	3.85340172104350	1.71608867947723	-6.37373265542016
H	5.04421886544018	0.43792079248842	-6.61086318967879
C	-0.32265995562166	-8.92895047133823	-3.08009439868141
H	0.42363146222957	-9.27046453860601	-3.79376627614162
H	-1.23337745431248	-9.50237523730425	-3.23786865359562
H	-0.53252467340509	-7.88130026763539	-3.28192584710569
C	0.47750666662164	-10.59983411742466	-1.40844016530278
H	-0.42203645173002	-11.19282411466466	-1.55690426668677
H	1.23732800894148	-10.95266725724798	-2.10170439493382
H	0.83684334811284	-10.76569647130790	-0.39517965415006
C	0.32266007711916	8.92895078440595	-3.08009444001177
H	-0.42363133368514	9.27046467126482	-3.79376643038495
H	1.23337762917457	9.50237550204124	-3.23786869736789
H	0.53252480054530	7.88130055758070	-3.28192562708296
C	-0.47750623700679	10.59983442881626	-1.40844033193070
H	0.42203690132094	11.19282435483935	-1.55690454764446
H	-1.23732752339884	10.95266753236843	-2.10170460435000
H	-0.83684295418494	10.76569705897731	-0.39517989641771
C	-2.82603688135617	-0.85537296864920	-3.99653428346869
H	-2.58680607356600	-1.89650009136606	-4.20021893844859
H	-2.03283189838872	-0.23455450066044	-4.40701558576688
H	-2.84531423438589	-0.71664064505148	-2.91779951028186
C	-4.09525344213912	-0.68241311783712	-6.13875604049615
H	-3.32356171587813	-0.04499131148075	-6.56436439383193
H	-3.85340205089421	-1.71608856471834	-6.37373264803448
H	-5.04421921681178	-0.43792079465238	-6.61086328755809
N	2.25530486642950	6.67514495287406	-0.30284579407732
N	4.61524298012003	-2.43354695371899	-2.36929264093570
N	-4.61524208839337	2.43354688936023	-2.36929220496779
N	-2.25530516979906	-6.67514476232959	-0.30284613604654
H	2.40610922330405	5.67119339137905	-0.32174659482891
H	4.75164443493519	-2.55721288604447	-1.36847331365160
H	-2.40610968422474	-5.67119324854069	-0.32174675450317
H	-4.75164390089610	2.55721221399435	-1.36847297223955
C	-4.43144133055808	3.67954114395321	-3.07689261288547
H	-3.55103361177977	4.19097286445772	-2.67662979766639
H	-4.27899082156342	3.49723792876010	-4.14107023671897
C	3.36825779127876	7.44849479904857	0.21110721766950
H	3.78755185167597	8.07319952262177	-0.58916266571969
H	3.02743760087573	8.10336028071190	1.01922769747241
C	4.43144169358286	-3.67954121018682	-3.07689326512216
H	3.55103308203957	-4.19097215491198	-2.67663125016385
H	4.27899201235205	-3.49723750832545	-4.14107093527547
C	-3.36825785999826	-7.44849479812673	0.21110682765481
H	-3.78755190170970	-8.07319926315374	-0.58916322783470
H	-3.02743767766051	-8.10336046680314	1.01922715231920
C	-5.63888997493745	4.57067719923309	-2.89178765822538
C	-5.49528882079806	5.84792134997578	-2.36834773350613
C	-6.90533353760734	4.11581643405242	-3.23760664523236

C	-6.60192110231830	6.66386924282749	-2.20519797111559
H	-4.51132985809927	6.19537751944367	-2.09044200581103
C	-8.01154965136297	4.92917008705168	-3.06920948999180
H	-7.02643182678743	3.11795333839942	-3.63416050433163
C	-7.86132127301504	6.20664840608959	-2.55508009211700
H	-6.48117820160757	7.65753633239857	-1.79893606665547
H	-8.99236803138445	4.56642035735591	-3.34039672629278
H	-8.72487121357334	6.84249986728010	-2.42525915229516
C	4.43512815346149	6.51538767973723	0.72058042897472
C	5.09656958402521	5.67368547177322	-0.16661885797756
C	4.76011307645437	6.46604320016994	2.06846496430705
C	6.06287705330464	4.79371370507804	0.28565531676609
H	4.85157148509669	5.70125668756478	-1.21821230785133
C	5.73237115179275	5.58999353619531	2.52204969585369
H	4.25221240522650	7.11324493091890	2.76837025333593
C	6.38118504028498	4.75000039648968	1.63322098205901
H	6.55605174965753	4.13615955637591	-0.41353523044798
H	5.98268111947471	5.56189228003889	3.57273424794181
H	7.13133988831068	4.05911739034567	1.98842347235758
C	5.63888982466107	-4.57067746104464	-2.89178710908865
C	5.49528818408522	-5.84792139505755	-2.36834614435786
C	6.90533347726989	-4.11581765390540	-3.23760634595056
C	6.60192051920465	-6.66386890281441	-2.20519567515230
H	4.51132923527677	-6.19537674498480	-2.09043991422335
C	8.01154960505048	-4.92917131106117	-3.06920902944714
H	7.02643222487729	-3.11795497708607	-3.63416166303168
C	7.86132067002773	-6.20664921145483	-2.55507802523615
H	6.48117723240734	-7.65753557378748	-1.79893276053230
H	8.99236808145402	-4.56642202246938	-3.34039615499158
H	8.72487063168276	-6.84250043887201	-2.42525655919438
C	-4.43512796602340	-6.51538759030858	0.72058006077264
C	-5.09656976793288	-5.67368548094342	-0.16661896873297
C	-4.76011286425844	-6.46604296203634	2.06846455542046
C	-6.06287740473558	-4.79371368089658	0.28565537248798
H	-4.85157166131010	-5.70125666478810	-1.21821239130491
C	-5.73237117237385	-5.58999333762164	2.52204939842656
H	-4.25221197479714	-7.11324468992512	2.76836972361286
C	-6.38118526922907	-4.75000006843932	1.63322101270352
H	-6.55605217554415	-4.13615943694575	-0.41353495461040
H	-5.98268097350064	-5.56189184787575	3.57273396866093
H	-7.13134017146701	-4.05911716120381	1.98842355864088

C_4 crown-*in* in chloroform
Energy total = -359.518468104208 a.u.

Symbol	X	Y	Z
C	-3.13717909186441	0.61859480966126	2.45534581965759
C	-3.87180893825116	1.10475280528953	1.36791624234939
C	-3.62403109187933	2.37224912987553	0.83836041428077
C	-2.58793061568811	3.13572783134978	1.38341292462695
C	-1.81221054468751	2.65172291300436	2.43760962666376
C	-2.09778230308198	1.39205404107327	2.93822205633465
O	-4.86959314382787	0.31144307229268	0.85871252967214
O	-2.39043329538987	4.39379397353192	0.87015776586654
H	-1.49691508556819	1.01708101055255	3.74774217793408
C	-0.72252024553056	3.50701528703715	3.05930587700024
C	0.61852753384136	3.13715285963280	2.45534779514384
C	1.10459921606415	3.87169998673706	1.36783071812481
C	2.37203882634278	3.62385381384917	0.83816380756597
C	3.13555250962454	2.58777619744748	1.38320475925422
C	2.65163411616666	1.81214516627064	2.43751280781505
C	1.39201997024870	2.09777239523493	2.93821907674104
O	0.31127898815260	4.86948570192031	0.85863921993620
H	1.01711429589741	1.49696946190118	3.74781806091602
C	3.50698340632008	0.72250739628841	3.05922327739240
C	3.13717909208745	-0.61859480971562	2.45534581984125
C	3.87180893869409	-1.10475280539994	1.36791624262743
C	3.62403109225573	-2.37224912976331	0.83836041426744
C	2.58793061588568	-3.13572783129946	1.38341292427775
C	1.81221054489347	-2.65172291302683	2.43760962650533
C	2.09778230320196	-1.39205404116599	2.93822205622211
O	4.86959314449153	-0.31144307237855	0.85871253034754
O	2.39043329558123	-4.39379397324004	0.87015776511119

H	1.49691508559526	-1.01708101067140	3.74774217781270
C	0.72252024573651	-3.50701528711544	3.05930587677560
C	-0.61852753364648	-3.13715285969473	2.45534779494998
C	-1.10459921587946	-3.87169998685675	1.36783071795479
C	-2.37203882611324	-3.62385381381615	0.83816380722383
C	-3.13555250934691	-2.58777619736726	1.38320475898954
C	-2.65163411602304	-1.81214516629421	2.43751280766659
C	-1.39201997009818	-2.09777239532929	2.93821907664484
O	-0.31127898802497	-4.86948570221894	0.85863922000820
O	-4.39355529848280	-2.39022100568947	0.86983775154798
H	-1.01711429572178	-1.49696946202687	3.74781806088708
C	-3.50698340617041	-0.72250739636646	3.05922327715954
C	-0.77446584365326	3.45040980719757	4.59598684082654
C	3.45041080586456	0.77450706574576	4.59590490087212
H	4.55190156740899	0.91857390504541	2.79596771158361
C	0.77446584381564	-3.45040980726222	4.59598684065747
H	0.91854518945174	-4.55195071615191	2.79608694672647
C	-3.45041080584791	-0.77450706581397	4.59590490068583
H	-4.55190156724866	-0.91857390514290	2.79596771119430
H	-1.82380700810285	3.34342836906161	4.88123967500417
H	-0.23489289851709	2.58523084691228	4.97719771960476
C	-0.22351425524873	4.70921136049428	5.27637569871142
H	3.34336232582324	1.82385067157806	4.88112262545231
H	2.58527153141382	0.23489111652881	4.97714458660517
C	4.70925150256932	0.22366064714057	5.27630423667503
H	1.82380700823342	-3.34342836909723	4.88123967488105
H	0.23489289870068	-2.58523084695742	4.97719771941392
C	0.22351425531609	-4.70921136048016	5.27637569861277
H	-3.34336232579563	-1.82385067163060	4.88112262529540
H	-2.58527153144302	-0.23489111663510	4.97714458650704
C	-4.70925150252536	-0.22366064721889	5.27630423649902
H	-0.91854518924577	4.55195071607063	2.79608694692359
C	-2.95250846900189	-4.51880010265651	-0.23903659705699
H	0.49490638655279	4.96760346873946	-0.12426548067721
H	4.96783487684747	-0.49520033335323	-0.12415250440533
H	1.44748638257015	-4.64785723365488	0.96851473275541
H	-0.49490638607683	-4.96760346899434	-0.12426548073069
H	-4.64769739182409	-1.44731292118061	0.96835658523971
H	-4.96783487576558	0.49520033319531	-0.12415250539457
O	4.39355529867653	2.39022100577597	0.86983775174092
C	-4.51906661941958	2.95282013921535	-0.23871137949998
H	-1.44748638232036	4.64785723371661	0.96851473353951
C	2.95250846897452	4.51880010280380	-0.23903659679409
H	4.64769739225227	1.44731292134060	0.96835658566748
C	-4.03456273904125	2.77134179800135	-1.66877315947164
C	-3.40504660052689	3.79282485217080	-2.37053530064017
C	-4.31357345097075	1.53833065168060	-2.29355958047986
O	-4.91708176080435	0.59576521243330	-1.72110825624291
C	2.77087263687071	4.03414811290669	-1.66902590756473
C	3.79228672519451	3.40458368914066	-2.37084330072142
C	1.53778186017087	4.31307146276236	-2.29369786597403
O	0.59528202792756	4.91665044887129	-1.72121398791532
C	4.51906661993482	-2.95282013947344	-0.23871137912684
C	4.03456273981451	-2.77134179780305	-1.66877315952455
C	4.31357345119909	-1.53833065288797	-2.29355958046462
O	4.91708176122686	-0.59576521243729	-1.72110825607115
C	3.40504660106847	-3.79282485401725	-2.37053529965323
C	-2.77087263715885	-4.03414811269870	-1.66902590777324
C	-1.53778185977003	-4.31307146254497	-2.29369786660431
O	-0.59528202788734	-4.91665044840004	-1.72121398758400
C	-3.79228672586699	-3.40458368884777	-2.37084329984937
H	-2.47312903926376	-5.50022093970205	-0.16176862273349
H	-4.01221738025818	-4.66461211803516	-0.02525141404105
H	-5.50047918303195	2.47342922736926	-0.16139966955228
H	-4.66485878470251	4.01250808786846	-0.02480689432718
H	2.47312903935713	5.50022093981851	-0.16176862239354
H	4.01221738021357	4.66461211819808	-0.02525141379458
H	5.50047918356693	-2.47342922748447	-0.16139966940237
H	4.66485878510512	-4.01250808789398	-0.02480689358301
C	1.27757715701173	4.87322560648080	5.05319598283451
C	-0.52498638453628	4.65545791477108	6.77466607276788
C	-4.65551944141286	-0.52525807619524	6.77457071919048
C	-4.87331094511942	1.27744323273868	5.05324228302862
C	0.52498638459939	-4.65545791474520	6.77466607268279
C	-1.27757715695772	-4.87322560644868	5.05319598277212
C	4.65551944144192	0.52525807615644	6.77457071932784

C	4.87331094516138	-1.27744323278561	5.05324228312288
H	-0.73480342801254	5.58268351959275	4.85378553695451
H	5.58269432221520	0.73494781749425	4.85365119950696
H	0.73480342808342	-5.58268351959600	4.85378553690314
H	-5.58269432217987	-0.73494781756123	4.85365119935331
H	1.50674751520219	4.97617060356842	3.99604305277486
H	1.81356913946397	4.00638957900461	5.43356011718981
H	1.64146815603341	5.75811073783608	5.57145432781775
H	-0.03448418111920	3.79670740930718	7.22873033807530
H	-1.59567497333997	4.57396346474266	6.95001313739694
H	-0.16476709744979	5.55532680443494	7.26811553109672
H	-3.79679384953062	-0.03476812707778	7.22869542238802
H	-4.57399693875075	-1.59595953274521	6.94982847376805
H	-5.55540992450463	-0.16510782815756	7.26802989284939
H	-4.97624969448000	1.50669346671575	3.99610594110326
H	-4.00649745538769	1.81343365201482	5.43366099180297
H	-5.75821541118767	1.64126076382828	5.57151847840352
H	0.03448418116740	-3.79670740929757	7.22873033798292
H	1.59567497339460	-4.57396346471964	6.95001313735016
H	0.16476709749033	-5.55532680439542	7.26811553100926
H	-1.50674751515029	-4.97617060350112	3.99604305269860
H	-1.81356913937715	-4.00638957895687	5.43356011715793
H	-1.64146815595584	-5.75811073782357	5.57145432775925
H	3.79679384957439	0.03476812705364	7.22869542254803
H	4.57399693878427	1.59595953270387	6.94982847387451
H	5.55540992454135	0.16510782814828	7.26802989298585
H	4.97624969453013	-1.50669346675881	3.99610594120522
H	4.00649745546258	-1.81343365210650	5.43366099188311
H	5.75821541123569	-1.64126076389071	5.57151847847217
C	3.84110409455662	-1.31698640006625	-3.71222314769778
H	4.44277947475370	-0.52050093482681	-4.15494877035526
H	2.80556387855437	-0.96547569817441	-3.65529779252328
C	1.31624772758967	3.84037192842811	-3.71225389516149
H	0.51968882537420	4.44196941377698	-4.15495250722897
H	0.96476312737007	2.80483319888138	-3.65512437204904
C	-3.84110409461122	1.31698639952525	-3.71222314780708
H	-4.44277947549085	0.52050093447579	-4.15494877014678
H	-2.80556387879345	0.96547569701481	-3.65529779325829
C	-1.31624772764132	-3.84037192852880	-3.71225389501673
H	-0.51968882539642	-4.44196941350057	-4.15495250771142
H	-0.96476312754387	-2.80483319873105	-3.65512437224592
C	-2.58914495066436	-3.90638674006929	-4.55262302127104
C	-3.66252847399842	-3.08775629846666	-3.82943546473972
H	-3.42745106223697	-2.02119914643129	-3.90610840461991
H	-4.62222155369172	-3.24234225955648	-4.32323046717635
C	-3.90721350537854	2.59000355604666	-4.55239906335088
C	-3.08842128575330	3.66324515022807	-3.82918635575011
H	-2.02188816132281	3.42811968362364	-3.90604205281852
H	-3.24302671754084	4.62301086408765	-4.32283516274465
C	2.58914494993447	3.90638674082230	-4.55262302184163
C	3.66252847404011	3.08775629867541	-3.82943546415701
H	3.42745106183027	2.02119914689614	-3.90610840530056
H	4.62222155321149	3.24234225992625	-4.32323046742693
C	3.90721350562915	-2.59000355672387	-4.55239906318188
C	3.08842128660581	-3.66324515114173	-3.82918635583329
H	2.02188816207987	-3.42811968507398	-3.90604205195653
H	3.24302671855126	-4.62301086519789	-4.32283516250123
C	5.35939651768349	-3.04913907257450	-4.70418210075918
H	5.41115885046122	-3.95427477440266	-5.30486517207147
H	5.95090759972807	-2.27916603772429	-5.19435635399083
H	5.80285540880847	-3.25212409482087	-3.73212991001913
C	3.30896726033473	-2.33451481587486	-5.93788062498065
H	2.27824407147309	-1.99600827949518	-5.85808497584651
H	3.88122038843671	-1.57304262719584	-6.46221474600191
H	3.32715232463578	-3.24496958135370	-6.53216590094220
C	-3.04819844344290	-5.35856227831257	-4.70470989329307
H	-3.95324476184411	-5.41026394330859	-5.30553237410226
H	-2.27812991327321	-5.94996007467820	-5.19487117916941
H	-3.25130543879785	-5.80219065704588	-3.73276012629105
C	-2.33347103211882	-3.30790565770030	-5.93796948830076
H	-1.99503832529020	-2.27717565537797	-5.85795619204729
H	-1.57188320575262	-3.88003023818856	-6.46227530667389
H	-3.24382957592599	-3.32604946966741	-6.53240182592180
C	-5.35939651723216	3.04913907255733	-4.70418210047368
H	-5.41115884980414	3.95427477450378	-5.30486517160775
H	-5.95090759963695	2.27916603798175	-5.19435635373568

H	-5.80285540813388	3.25212409480938	-3.73212990963105
C	-3.30896726040379	2.33451481531428	-5.93788062526144
H	-2.27824407155491	1.99600827881602	-5.85808497634710
H	-3.88122038870005	1.57304262682316	-6.46221474637192
H	-3.32715232467746	3.24496958095521	-6.53216590099688
C	3.04819844382039	5.35856227831227	-4.70470989317876
H	3.95324476205452	5.41026394348342	-5.30553237435358
H	2.27812991328056	5.94996007464996	-5.19487117915847
H	3.25130543915080	5.80219065734424	-3.73276012628338
C	2.33347103237159	3.30790565726439	-5.93796948799803
H	1.99503832550752	2.27717565487948	-5.85795619206233
H	1.57188320603526	3.88003023779039	-6.46227530690410
H	3.24382957633672	3.32604946936845	-6.53240182554259
N	-3.07492358255241	4.93004161448788	-1.76755260719243
N	-4.92960438736063	-3.07459561217173	-1.76797203970294
N	3.07492358517489	-4.93004161535724	-1.76755260843712
N	4.92960438663740	3.07459561259250	-1.76797204039100
H	4.91729253830972	3.06060090738394	-0.75175226100306
H	-3.06082289984730	4.91760291628251	-0.75133399512038
H	-4.91729253718793	-3.06060090653393	-0.75175226061292
H	3.06082290224431	-4.91760291755177	-0.75133399508139
C	2.37915237964750	-6.06493133045373	-2.33824983912718
H	1.32384265660119	-5.81267759697183	-2.49472992966725
H	2.83152588884534	-6.33673497282424	-3.29606739243171
C	6.06447951022284	2.37886586130990	-2.33874543480706
H	5.81227725234158	1.32353706673501	-2.49517734163970
H	6.33618245936770	2.83123286884623	-3.29659581538433
C	-2.37915237734766	6.06493133011250	-2.33824983802881
H	-1.32384265527398	5.81267759983722	-2.49472992354281
H	-2.83152588526781	6.33673496854399	-3.29606739460977
C	-6.06447950917072	-2.37886586134551	-2.33874543554640
H	-5.81227725294381	-1.32353706756298	-2.49517734117890
H	-6.33618245916269	-2.83123286856942	-3.29659581410019
C	2.48627580110881	-7.22208453186950	-1.38059556163785
C	1.38346797415600	-7.63535997293656	-0.64715953234830
C	3.70495143691710	-7.86394745739673	-1.19575455116302
C	1.49445578795369	-8.67879120683102	0.25570206898108
H	0.43914159317577	-7.13112001865830	-0.78449291621874
C	3.81666844720467	-8.90655358363739	-0.29337965462744
H	4.56871747501716	-7.54321510794207	-1.76019899816103
C	2.71085861972503	-9.31581014285423	0.43389923338685
H	0.63048871217992	-8.99100108976693	0.82381600666729
H	4.76736714217176	-9.40064642335743	-0.15578939144979
H	2.79791444805070	-10.12902974347026	1.13942675777856
C	7.22169867747653	2.48606185769076	-1.38117801048009
C	7.63508910134742	1.38327520399425	-0.64777595554564
C	7.86350157329257	3.70477396288575	-1.19637665646256
C	8.67856702090941	1.49432168492662	0.25502285297514
H	7.13089996923894	0.43891929143021	-0.78508600538730
C	8.90615735085113	3.81654679896630	-0.29406657183901
H	7.54268561143381	4.56852378098746	-1.76079887145555
C	9.31552005925178	2.71076220781472	0.43318932922786
H	8.99086078232476	0.63037229064354	0.82311845238611
H	9.40020349759248	4.76727437607646	-0.15650274726447
H	10.12877510834937	2.79786427149162	1.13867148027679
C	-2.48627582681495	7.22208453495842	-1.38059556736568
C	-1.38346796628833	7.63535998462681	-0.64715951808222
C	-3.70495142573673	7.86394744507540	-1.19575456101887
C	-1.49445577910643	8.67879119033661	0.25570205766325
H	-0.43914160193283	7.13112000788299	-0.78449292609697
C	-3.81666844115800	8.90655360125705	-0.29337963945183
H	-4.56871748492572	7.54321512291266	-1.76019899261016
C	-2.71085863597304	9.31581014521467	0.43389922942091
H	-0.63048871760811	8.99100109407341	0.82381601125630
H	-4.76736714568404	9.40064642122821	-0.15578939782895
H	-2.79791443469039	10.12902974286733	1.13942676140646
C	-7.22169868052860	-2.48606185820840	-1.38117800823212
C	-7.63508910445398	-1.38327520736508	-0.64777595450508
C	-7.86350157557105	-3.70477395900410	-1.19637665359584
C	-8.67856702071390	-1.49432168756893	0.25502285078626
H	-7.13089996971744	-0.43891929007763	-0.78508600553396
C	-8.90615734994104	-3.81654679610754	-0.29406657245469
H	-7.54268561226554	-4.56852378203045	-1.76079887175176
C	-9.31552005556093	-2.71076220697855	0.43318932544791
H	-8.99086078237081	-0.63037229006845	0.82311845196051
H	-9.40020349769996	-4.76727437618774	-0.15650274794478

H	-10.12877510934177	-2.79786427151638	1.13867148093491

C_4 crown-*out* in chloroform
Energy total = -359.525416637668 a.u.

Symbol	X	Y	Z
C	2.85154634462514	-1.45582780632149	1.35146380491146
C	3.72094260538547	-1.50318872126430	0.25880658974631
C	4.31807635512472	-0.34647678532369	-0.25144821450211
C	3.99397580097871	0.88297600432537	0.31883540975779
C	3.07120389292207	0.96150856334528	1.36742821064872
C	2.51229526179873	-0.20743006111476	1.84886593774108
O	4.05275108306998	-2.68622015797597	-0.35735319273418
O	4.52589266519615	2.06082302628927	-0.12885835916556
H	1.80179172389985	-0.13647884412402	2.65326395006814
C	2.73625411417829	2.31487508426803	1.96539689364413
C	1.45593943106332	2.85173163936531	1.35146176551629
C	1.50326410413260	3.72133158533479	0.25896734831909
C	0.34652553759377	4.31851948420791	-0.25115867994427
C	-0.88291010236861	3.99425184208024	0.31907356894432
C	-0.96140196382020	3.07124382652021	1.36746218689076
C	0.20756404511122	2.51229701038123	1.84878703002917
O	2.68629468832758	4.05327744999146	-0.35712748136797
H	0.13665760566409	1.80162412677750	2.65304033244810
C	-2.31472601759078	2.73614112661700	1.96544836735070
C	-2.85154634044211	1.45582769508483	1.35146382527104
C	-3.72094251274520	1.50318857732191	0.25880653402586
C	-4.31807637369644	0.34647663610628	-0.25144814753166
C	-3.99397589845122	-0.88297611041424	0.31883559659462
C	-3.07120390647232	-0.96150867227102	1.36742831545444
C	-2.51229527740062	0.20742997761266	1.84886602448828
O	-4.05275079862675	2.68621989674599	-0.35735352278595
O	-4.52589298292780	-2.06082315014879	-0.12885791904948
H	-1.80179179658590	0.13647886350167	2.65326409345517
C	-2.73625417208457	-2.31487518873873	1.96539703735598
C	-1.45593949865560	-2.85173182785827	1.35146192036687
C	-1.50326416081350	-3.72133169140847	0.25896742683719
C	-0.34652557592858	-4.31851958606408	-0.25115861266156
C	0.88291003538981	-3.99425184633653	0.31907356739868
C	0.96140191756030	-3.07124396563141	1.36746231276624
C	-0.20756413815912	-2.51229727748450	1.84878725419790
O	-2.68629473679075	-4.05327737033327	-0.35712750539605
O	2.06078042058503	-4.52621945771472	-0.12849676405037
H	-0.13665776798208	-1.80162447718237	2.65304063917734
C	2.31472599931110	-2.73614120411803	1.96544841187639
C	2.73166105038306	2.26870256951294	3.50379340958803
C	-2.26844164429922	2.73150299294831	3.50384446519634
H	-2.98907302848124	3.55533925434157	1.69237778021595
C	-2.73166120215050	-2.26870256029927	3.50379355312020
H	-3.55548604386474	-2.98914905884086	1.69224973262592
C	2.26844170827318	-2.73150290028356	3.50384451178943
H	2.98907300352351	-3.55533934625095	1.69237784170209
H	3.49460123848918	1.54736112444740	3.80584002973701
H	1.77405805747909	1.92102283141114	3.88632595098869
C	3.06360436447959	3.61428181080863	4.16060019720246
H	-1.54697231790546	3.49433238275316	3.80586535858335
H	-1.92086659022364	1.77384561019158	3.88633217446870
C	-3.61392404069812	3.06361900631816	4.16075461102226
H	-3.49460151993176	-1.54736118380197	3.80584002191725
H	-1.77405829536844	-1.92102267029634	3.88632617155662
C	-3.06360443629520	-3.61428177015101	4.16060043483065
H	1.54697232187497	-3.49433218060649	3.80586554557406
H	1.92086677348816	-1.77384541768819	3.88633207876700
C	3.61392408727201	-3.06361892106583	4.16075469827357
H	3.55548591469005	2.98914900865455	1.69224951384741
C	-0.43906502295712	-5.25439386728254	-1.43618949902723
H	3.36247185930572	3.36101955056181	-0.20826354009234
H	-3.36062836262853	3.36247474828096	-0.20821105796169
H	-5.28019335600335	-1.93518288020032	-0.76826805976724
H	-3.36247196507962	-3.36101962814644	-0.20826312408457
H	1.93517637964627	-5.28068784358180	-0.76772154938847
H	3.36062836034831	-3.36247483515811	-0.20821123531080
O	-2.06078046162834	4.52621972125917	-0.12849646291612

C	5.25372158180064	-0.43906097323635	-1.43665793488964
H	5.28019342663484	1.93518287063631	-0.76826819075237
C	0.43906504138060	5.25439386656976	-1.43618946182943
H	-1.93517627891223	5.28068788647691	-0.76772144297684
C	6.73478149986407	-0.33639170651378	-1.11021444865591
C	7.47512975713997	-1.42628106768911	-0.68609304865185
C	7.34621124649921	0.92754691589938	-1.29636218887198
O	6.68692040369586	1.95288280392269	-1.59485529386002
C	0.33638600116051	6.73539593673827	-1.10948344194533
C	1.42627534615206	7.47569437267040	-0.68527152304160
C	-0.92756686552938	7.34683973524043	-1.29549570866617
O	-1.95291563355610	6.68757544187844	-1.59400218873124
C	-5.25372159088866	0.43906085724321	-1.43665787906516
C	-6.73478150222256	0.33639165157257	-1.11021440023135
C	-7.34621133544388	-0.92754684651008	-1.29636219836716
O	-6.68692049386774	-1.95288279133666	-1.59485563476371
C	-7.47512973155919	1.42628115423987	-0.68609289290069
C	-0.33638582302321	-6.73539596276291	-1.10948369232204
C	0.92756698903064	-7.34683979631346	-1.29549579915622
O	1.95291588952975	-6.68757545197139	-1.59400183754450
C	-1.42627530133238	-7.47569429650959	-0.68527173522783
H	0.37425829462486	-5.02190706757195	-2.13236488643365
H	-1.37741723352413	-5.05565577988249	-1.95815453950013
H	5.02111939893162	0.37424958051791	-2.13280907964214
H	5.05487268002306	-1.37742026058212	-1.95856628781960
H	-0.37425836810439	5.02190728722353	-2.13236481519852
H	1.37741717771946	5.05565572632472	-1.95815462884587
H	-5.02111950464502	-0.37424979603293	-2.13280896186617
H	-5.05487257502426	1.37742007822268	-1.95856629342503
C	1.96066617210510	4.64794438662705	3.94700759103957
C	3.30076715304125	3.40992609872788	5.65767157054953
C	3.40943870323337	-3.30061668808543	5.65783379381789
C	4.64777958013482	-1.96087349669026	3.94710989329555
C	-3.30076713323640	-3.40992598923999	5.65767181165170
C	-1.96066626820115	-4.64794435080021	3.94700775700400
C	-3.40943873306580	3.30061688329945	5.65783369697200
C	-4.64777947667974	1.96087354024864	3.94710976025487
H	3.99039487523718	3.99631583242467	3.71574137490897
H	-3.99581940640233	3.99051510336103	3.71599572248618
H	-3.99039499368604	-3.99631580754790	3.71574172599593
H	3.99581945187603	-3.99051504671633	3.71599587257257
H	1.80000081175477	4.83740512204509	2.88955105462363
H	1.02443070228526	4.29277935741161	4.37187883539274
H	2.22430258271548	5.58607818828480	4.43119506889761
H	2.40311014408595	3.01981146440666	6.13323410045888
H	4.11376498133188	2.70710708929180	5.82747274282999
H	3.55825218355816	4.35265990075076	6.13535187762777
H	3.01945576324527	-2.40284964981590	6.13329695136584
H	2.70646252547471	-4.11347418631065	5.82765866951423
H	4.35209618620482	-3.55823151453190	6.13559368273333
H	4.83720543926800	-1.80024321392321	2.88964082141302
H	4.29281332866903	-1.02457373184811	4.37200595225145
H	5.58589267385247	-2.22468768558222	4.43123977145836
H	-2.40311012799814	-3.01981130042742	6.13323430462719
H	-4.11376494009060	-2.70710695564462	5.82747296767008
H	-3.55825218101414	-4.35265976550134	6.13535216374259
H	-1.80000104897337	-4.83740513602182	2.88955120827362
H	-1.02443074873489	-4.29277931462142	4.37187888206596
H	-2.22430262322274	-5.58607812710274	4.43119530976471
H	-3.01945585236110	2.40284988346745	6.13329697481169
H	-2.70646256259189	4.11347439841525	5.82765852396673
H	-4.35209623734366	3.55823177722365	6.13559350771867
H	-4.83720528323191	1.80024331492672	2.88964067102762
H	-4.29281319310356	1.02457375266286	4.37200573913683
H	-5.58589258782961	2.22468764231774	4.43123965232224
C	-8.83438987459917	-1.06315549098710	-1.08217988735729
H	-8.97949593564947	-1.35984564198490	-0.03790766072606
H	-9.19210639995983	-1.88640427536599	-1.70442550479960
C	-9.59701579776524	0.22895788518525	-1.36474942262411
C	-8.96154278396813	1.33811761650941	-0.51958497749820
H	-9.40947067714873	2.29297846148575	-0.79391673873630
H	-9.17726128529996	1.15194618976529	0.53764255238827
C	0.22894830194628	9.59763892434952	-1.36367112470916
C	1.33808072677728	8.96208012664598	-0.51853579282298
H	2.29294570675960	9.41006877187419	-0.79275110671413
H	1.15184318070249	9.17764225471485	0.53871208114101

C	-1.06317175889046	8.83499865505791	-1.08117048322996
H	-1.35986063258086	8.98001312057526	-0.03688522484810
H	-1.88642008635336	9.19277395710771	-1.70338320837473
C	-1.33808075485780	-8.96208004978544	-0.51853606824318
H	-2.29294576333676	-9.41006860670472	-0.79275142270676
H	-1.15184331143850	-9.17764226903505	0.53871179760273
C	-0.22894835150584	-9.59763894655753	-1.36367135011771
C	1.06317179126037	-8.83499877944740	-1.08117069822348
H	1.35986067747891	-8.98001315386728	-0.03688542436788
H	1.88642005906795	-9.19277426459599	-1.70338340001358
C	8.83438987035289	1.06315554390511	-1.08218026177151
H	8.97949621884577	1.35984597028525	-0.03790814733493
H	9.19210607511667	1.88640420429654	-1.70442624889267
C	9.59701576653992	-0.22895784595701	-1.36474977443462
C	8.96154280670001	-1.33811752394577	-0.51958520429474
H	9.40947075862878	-2.29297837280994	-0.79391680378191
H	9.17726135792714	-1.15194587262556	0.53764227029510
C	0.58200773688878	9.51229068792124	-2.85044206498693
H	-0.21093837268279	9.95117208609458	-3.45199854761777
H	1.50526396005308	10.04978436360530	-3.05451079707393
H	0.70875996067052	8.47743431139636	-3.15945816815998
C	0.06771947007902	11.06712690026653	-0.96826409470978
H	0.99385032458151	11.60950101142739	-1.14406859574890
H	-0.71885760353826	11.53256588193635	-1.55737397766927
H	-0.19390935349639	11.15955373659728	0.08357255531584
C	9.51155951645295	-0.58199566869280	-2.85151909760807
H	9.95036081220798	0.21097759520140	-3.45309864407195
H	10.04907244320564	-1.50522753091037	-3.05564801146401
H	8.47668198677661	-0.70878177043893	-3.16045120441496
C	11.06653041867223	-0.06771295144356	-0.96944527413592
H	11.60889653350342	-0.99384687744146	-1.14525671372045
H	11.53192792435815	0.71884948901993	-1.55860711608361
H	11.15902229881702	0.19394758316431	0.08237806929451
C	-9.51155959008932	0.58199566912998	-2.85151875895698
H	-9.95036051477826	-0.21097774093753	-3.45309833752440
H	-10.04907284870112	1.50522736356328	-3.05564769907689
H	-8.47668204703327	0.70878147870257	-3.16045093937797
C	-11.06653042067456	0.06771287581651	-0.96944488440951
H	-11.60889652147627	0.99384684163687	-1.14525592735929
H	-11.53192796512283	-0.71884935793997	-1.55860703664911
H	-11.15902229005725	-0.19394725062999	0.08237856279250
C	-0.58200766292158	-9.51229074739122	-2.85044233062906
H	0.21093853422746	-9.95117217839350	-3.45199871373851
H	-1.50526382445801	-10.04978444592023	-3.05451118844518
H	-0.70876036296525	-8.47743440819261	-3.15945836630066
C	-0.06771978413135	-11.06712694790456	-0.96826426566330
H	-0.99385077304676	-11.60950084263861	-1.14406888679816
H	0.71885721737160	-11.53256615992168	-1.55737402089059
H	0.19390950253042	-11.15955380764535	0.08357226701312
N	-6.88684226824034	2.58163117603500	-0.38102190982762
N	2.58164735589651	6.88738525729882	-0.38032801848878
N	-2.58164725581716	-6.88738514747996	-0.38032820124404
N	6.88684230456704	-2.58163120241860	-0.38102196219452
H	-5.88165562897733	2.64178759915838	-0.49574801360920
H	2.64182844693404	5.88222188934072	-0.49523532814713
H	5.88165566046440	-2.64178764617603	-0.49574778747441
H	-2.64182883405671	-5.88222178881126	-0.49523543893951
C	-3.79075059989479	-7.54657604205723	0.06198710514734
H	-3.55985172035649	-8.26640329071312	0.85215049306672
H	-4.43670477901895	-6.77265253228247	0.48565987218242
C	-7.54608460617529	3.79077214001628	0.06110816504390
H	-8.26611562425301	3.55992540400335	0.85110040705550
H	-6.77223687086079	4.43670413418849	0.48495499147573
C	3.79075068509054	7.54657618581643	0.06198730215170
H	3.55985163496297	8.26640336649747	0.85215071123989
H	4.43670489851983	6.77265271028874	0.48566006000257
C	7.54608468292535	-3.79077199725163	0.06110834860396
H	8.26611567693548	-3.55992506156301	0.85110054125396
H	6.77223695156189	-4.43670392815128	0.48495530812095
C	-4.52629216613606	-8.23544996103011	-1.06687009701928
C	-4.97030451329107	-9.54480290975049	-0.93649429190617
C	-4.76847074866789	-7.55326509772573	-2.25322228676245
C	-5.64180451623047	-10.16506452117283	-1.97646891934913
H	-4.79368210027680	-10.08257099528694	-0.01567996207595
C	-5.43372661332368	-8.17520574424239	-3.29466851513823
H	-4.43577575703230	-6.53117972892819	-2.35985790966440

C	-5.87100720050001	-9.48282706460041	-3.15921240716047
H	-5.98291558887412	-11.18378433163801	-1.86410166966456
H	-5.61474474958954	-7.63588613680021	-4.21289477145197
H	-6.39064960461223	-9.96713402999076	-3.97280304449668
C	-8.23464566213359	4.52631336891617	-1.06794092101489
C	-9.54396084576704	4.97051914163951	-0.93784944443397
C	-7.55220388346151	4.76829629906816	-2.25418548491603
C	-10.16393212007615	5.64201785795460	-1.97799846210770
H	-10.08192634373076	4.79405182968148	-0.01712062011462
C	-8.17385690191569	5.43354441218912	-3.29580815960824
H	-6.53014296601403	4.43545521389864	-2.36059926190057
C	-9.48144255290779	5.87101945156261	-3.16063564047982
H	-11.18262155583242	5.98329076189297	-1.86584672249183
H	-7.63433878554582	5.61440651037100	-4.21394885095937
H	-9.96552606576502	6.39065624830668	-3.97436366870433
C	4.52629223336905	8.23545015319918	-1.06686988631752
C	4.97030478556294	9.54480303111137	-0.93649409176548
C	4.76847069871886	7.55326524610363	-2.25322208318640
C	5.64180489721719	10.16506447285873	-1.97646874224872
H	4.79368251510688	10.08257122050696	-0.01567978628472
C	5.43372661886153	8.17520577008605	-3.29466835665106
H	4.43577559290793	6.53117992080653	-2.35985764480590
C	5.87100744587937	9.48282700113096	-3.15921224074613
H	5.98291625618805	11.18378419402995	-1.86410151091334
H	5.61474466281176	7.63588611297368	-4.21289460409455
H	6.39064992031254	9.96713386215585	-3.97280290547849
C	8.23464573403630	-4.52631336659447	-1.06794064135913
C	9.54396089672974	-4.97051916974976	-0.93784909845784
C	7.55220397461687	-4.76829632740178	-2.25418520977351
C	10.16393213825383	-5.64201803975309	-1.97799805588841
H	10.08192645724917	-4.79405181729120	-0.01712032749252
C	8.17385693675975	-5.43354456114227	-3.29580780217706
H	6.53014307198648	-4.43545515263205	-2.36059902688896
C	9.48144253171458	-5.87101971297203	-3.16063522808558
H	11.18262152951064	-5.98329098775128	-1.86584624909750
H	7.63433880794638	-5.61440669160419	-4.21394850919964
H	9.96552603671446	-6.39065664104197	-3.97436316662613

C_2 in DMSO

Energy total = -359.491513610977 a.u.

Symbol	X	Y	Z
C	-1.37734233723367	-2.81023210082263	1.72193230702131
C	-1.36643643992684	-3.58355085394301	0.55923425576550
C	-0.17399520803506	-4.10084847997989	0.04333589910111
C	1.02777096676996	-3.80694603859941	0.68528021994823
C	1.04418919563475	-2.98669627269935	1.81915017922978
C	-0.15621723624293	-2.49509915346037	2.29679449667558
O	-2.51602466867680	-3.89792891096000	-0.12142513812245
O	2.22720150691932	-4.28039021260040	0.23569311382479
H	-0.13404032716092	-1.85986720173421	3.16548902137768
C	2.36808591367014	-2.67914508323480	2.49344175325379
C	2.96043114704171	-1.42481520488443	1.87838243056368
C	3.96321495823838	-1.49922962295461	0.90825829155234
C	4.53045886728696	-0.34540610730651	0.35857023546040
C	4.08205571078133	0.90261613465932	0.79125042523197
C	3.08904457620924	0.99696710278597	1.77291471531494
C	2.53057522223051	-0.16610673955167	2.26706750753581
O	4.46366082500808	-2.70514504190512	0.47463525095448
H	1.74330834077689	-0.08122303431989	2.99710523352158
C	2.69728516489594	2.35872522770785	2.31424820603746
C	1.37734273166390	2.81023188428912	1.72193378052586
C	1.36643641922771	3.58355090812989	0.55923604851362
C	0.17399581848113	4.10084844648428	0.04333666603097
C	-1.02777121628508	3.80694604511398	0.68528041999709
C	-1.04418892215985	2.98669577288364	1.81914943754758
C	0.15621726826293	2.49509833139173	2.29679415984460
O	2.51602625934895	3.89792950329051	-0.12142273820680
O	-2.22720043358329	4.28039106256651	0.23569326815964
H	0.13403827528826	1.85986608768161	3.16548829302635
C	-2.36808518695274	2.67914450406427	2.49344134142576
C	-2.96043006889286	1.42481451156748	1.87838183480314
C	-3.96321584417589	1.49922834387395	0.90825714688892
C	-4.53045666336575	0.34540694229194	0.35856580015277

C	-4.08205308948085	-0.90261815853556	0.79124274169979
C	-3.08904578374723	-0.99696765283707	1.77290937295558
C	-2.53057634581334	0.16610612533646	2.26706536895692
O	-4.46366302439541	2.70514581442726	0.47463901405619
O	-4.58183706981121	-2.07274471620629	0.29786690887739
H	-1.74331082737450	0.08121537590979	2.99710353742309
C	-2.69728599121917	-2.35872534954112	2.31424558958403
C	2.24426158476662	-2.62627374839353	4.02535289330719
C	2.74538885303215	2.37162912316528	3.85228189707549
H	3.47437654736751	3.06248033201553	1.99654370599416
C	-2.24426058028549	2.62627387713054	4.02535253780271
H	-3.03243322356671	3.52339370616149	2.28386631731191
C	-2.74538905124282	-2.37162861924856	3.85227972989714
H	-3.47437715748058	-3.06248151900463	1.99654139890613
H	1.49912832004293	-3.36963599762802	4.32085090683688
H	1.89356321105654	-1.65099489706600	4.35970601777749
C	3.54971061197092	-2.94932531653501	4.76195197122061
H	3.54357168110624	1.69118108730624	4.16050625398006
H	1.81655262766365	2.00280879207992	4.28437990182925
C	3.04982577170528	3.75389353968715	4.43975133472895
H	-1.49912672567111	3.36963665301035	4.32085109556322
H	-1.89356212819741	1.65099581684311	4.35970542371640
C	-3.54971081457567	2.94932514997094	4.76194984268457
H	-3.54357076970300	-1.69118050349763	4.16050480315333
H	-1.81655175011495	-2.00280990889486	4.28437839141788
C	-3.04982748382854	-3.75389253201753	4.43974994541814
H	3.03243430130067	-3.52339474611384	2.28386581258441
C	-5.67389541413182	0.46084936799704	-0.62705430384354
H	3.77440937475126	-3.39133494577869	0.55897520137886
H	3.24373131923500	3.28532164751203	0.10295117268242
H	-2.12640051527871	4.92943463367957	-0.50007519252951
H	-3.77441316217958	3.39133657910565	0.55898146835181
H	-5.20018579471625	-1.93072178013173	-0.45984818428415
H	-3.24373169429897	-3.28532317758032	0.10294772670157
O	4.58184236689386	2.07274587500046	0.29788075096193
C	-0.20499403998060	-4.93837178835698	-1.21610032915978
H	2.12640261293515	-4.92943448512437	-0.50007454975994
C	5.67389575681661	-0.46084936431000	-0.62704955128380
H	5.20019270512617	1.93072071726516	-0.45982945901570
C	-0.00146656124014	-6.42680189901297	-0.99711657397569
C	-1.01109535137487	-7.24009642598374	-0.52450714784787
C	1.28069856913173	-6.95275822838578	-1.31104532756572
O	2.24144594537229	-6.21992888481441	-1.62029063999073
C	5.31011451653952	-0.25178339484811	-2.08726361481208
C	4.81455123643791	-1.27809500299224	-2.86681827695907
C	5.59851612371269	1.02775194175108	-2.63879197357786
O	6.02416148888039	1.97573881272374	-1.95236533868045
C	0.20499456159347	4.93837331135354	-1.21609922518725
C	0.00146725918432	6.42680313924151	-0.99711311922008
C	-1.28069811165196	6.95276076628221	-1.31104149770497
O	-2.24144702744288	6.21993143346222	-1.62028203714755
C	1.01109693353437	7.24009686435520	-0.52450455019703
C	-5.31011713606777	0.25178657987191	-2.08726905974936
C	-5.59850376392590	-1.02774988383784	-2.63879660297712
O	-6.02415606979390	-1.97573565155069	-1.95237152008670
C	-4.81455283153177	1.27809944871481	-2.86682190118494
H	-6.14232966653119	1.43871735221828	-0.50014109331799
H	-6.42977163413921	-0.28953510026648	-0.37453540663848
H	0.58554196958371	-4.59830720842230	-1.89194461744473
H	-1.15880001054088	-4.76818348952680	-1.71804965481823
H	6.14232744253385	-1.43871895764098	-0.50013837581624
H	6.42977340922018	0.28953335972897	-0.37453001870879
H	-0.58554124992011	4.59830946187981	-1.89194404710354
H	1.15880125204815	4.76818626097493	-1.71804758049738
C	4.60701457251841	-1.86649274246232	4.56178908980280
C	3.26683497556667	-3.12932035940453	6.25472554877634
C	-3.27729215578524	-3.63575995616713	5.94768671308663
C	-1.93760467809638	-4.76005462136887	4.15659264030259
C	-3.26683771910876	3.12932099146106	6.25472375358611
C	-4.60701352576635	1.86649159640932	4.56178560092291
C	3.27729059620642	3.63576062927907	5.94768799999584
C	1.93760102004975	4.76005325971608	4.15659294413730
H	3.94490263514453	-3.89585429315362	4.37306996897729
H	3.97267514572318	4.12461109269183	3.97906396549168
H	-3.94490237826237	3.89585385947473	4.37306679334117
H	-3.97267774489713	-4.12460944529192	3.97906320568581

H	4.87306817286143	-1.77046090230729	3.51389736416898
H	4.23204912250017	-0.90503706624860	4.90610412398586
H	5.50613015520307	-2.10751712935941	5.12571625748253
H	2.86694836329130	-2.20957652141287	6.67717190644589
H	2.54410826492852	-3.92610148725434	6.41831347953282
H	4.18012874668382	-3.38122370637204	6.78939364884360
H	-2.37887648940990	-3.26702304065253	6.43924596563561
H	-4.09239339946098	-2.94767874727912	6.16203604115307
H	-3.52620738508964	-4.60457902130329	6.37534872588315
H	-1.82179089444664	-4.91706370418297	3.08854793533708
H	-0.99034607459623	-4.40068474206434	4.55257578881502
H	-2.16571803135311	-5.71506934201996	4.62569841363810
H	-2.86695120327923	2.20957773740335	6.67717148232164
H	-2.54411154856702	3.92610229049673	6.41831320690033
H	-4.18013254206596	3.38122348639334	6.78938999122349
H	-4.87306482873582	1.77045959603796	3.51389337106111
H	-4.23204786158828	0.90503638628557	4.90610230919399
H	-5.50613054979766	2.10751532980578	5.12571122651554
H	2.37887521625037	3.26702178768521	6.43924638387526
H	4.09239274882426	2.94767975840734	6.16203685648697
H	3.52620492275063	4.60457976506980	6.37534989787719
H	1.82178826279046	4.91706149142938	3.08854799288622
H	0.99034334350157	4.40068107656987	4.55257593380533
H	2.16571181309302	5.71506918488665	4.62569798018978
C	-1.50419841099373	8.44682447690200	-1.21493327611050
H	-1.93471716593746	8.63935836111402	-0.22644494220136
H	-2.25700453778460	8.72994934092608	-1.95366147564087
C	-0.22728400048651	9.26388491160261	-1.38732127478121
C	0.80958234139294	8.72153261533902	-0.39665171636455
H	1.75752041597403	9.23862724696802	-0.55220661176398
H	0.47994277086105	8.93895750455586	0.62500589134125
C	4.28388707012389	0.38378870274475	-4.70210279311237
C	4.54467806371742	-1.08043916260652	-4.33217854514302
H	3.67778420139680	-1.67466865950194	-4.62707882417929
H	5.40620743357106	-1.44586642447462	-4.90122422639371
C	5.42673401822306	1.21924071028297	-4.13195675462872
H	6.36915213275238	0.92894430763148	-4.60826401364066
H	5.27607479040681	2.28264406949278	-4.32809853485537
C	-4.54467663327837	1.08044237456921	-4.33218081758490
H	-3.67777578524396	1.67466367145161	-4.62707803609426
H	-5.40619963968143	1.44587705009432	-4.90123240108319
C	-4.28389203880982	-0.38378642317361	-4.70210896763631
C	-5.42673989714612	-1.21923759786531	-4.13196349562840
H	-6.36916348537018	-0.92894072955727	-4.60826891653672
H	-5.27608416659387	-2.28263873129630	-4.32810200428372
C	1.50419629894020	-8.44682359418830	-1.21494524576037
H	1.93471717254207	-8.63935909530920	-0.22646008054569
H	2.25700049526007	-8.72994852274689	-1.95367705731546
C	0.22728164805038	-9.26388397200467	-1.38732939897832
C	-0.80958011490601	-8.72153207288275	-0.39665442953145
H	-1.75751865135207	-9.23862725807610	-0.55220320933368
H	-0.47993267081918	-8.93895498220376	0.62500092271469
C	2.94539712258100	0.84063138796496	-4.11770693124184
H	2.77996439089940	1.89396170175676	-4.33009412304462
H	2.12717959306181	0.26996757523789	-4.55151507542709
H	2.92806230611736	0.70076506531064	-3.04062668222738
C	4.24383561741805	0.52580409551126	-6.22631243746722
H	3.45307109700235	-0.09211814045963	-6.64636431672254
H	4.05411586451947	1.55978938006839	-6.50433804759297
H	5.18973518766356	0.22153788891902	-6.67004767845891
C	-0.29623334896863	-9.13777955091153	-2.82059181630947
H	0.44042273175181	-9.51736406509556	-3.52484220887802
H	-1.21280944327508	-9.71079185608320	-2.94330165267153
H	-0.50053165051662	-8.09875650750357	-3.06566338238295
C	0.50449865202663	-10.73826801311398	-1.08188488617414
H	-0.40299667256524	-11.32804863554060	-1.19091281168856
H	1.25184252627591	-11.13149669175352	-1.76694230784621
H	0.87460930731564	-10.86080334505757	-0.06612960553318
C	0.29622167501949	9.13778062244136	-2.82058694747239
H	-0.44044010280166	9.51736312162064	-3.52483264726240
H	1.21279605539463	9.71079388417544	-2.94330634998297
H	0.50051926186655	8.09875755042144	-3.06565867188483
C	-0.50449837317772	10.73826849918489	-1.08187403039517
H	0.40299731800725	11.32804811525359	-1.19090455934195
H	-1.25184425002785	11.13149904909626	-1.76692832952490
H	-0.87460514177946	10.86080338849488	-0.06611714035688

C	-2.94540638133248	-0.84064270587648	-4.11771443028052
H	-2.77998796876836	-1.89397574130177	-4.33010134809629
H	-2.12717818180846	-0.26999142467101	-4.55151849485087
H	-2.92807129801316	-0.70078054426300	-3.04063333390613
C	-4.24384018868518	-0.52579329504190	-6.22631903501612
H	-3.45307260787402	0.09212900535735	-6.64636336655125
H	-4.05412562243896	-1.55977766860325	-6.50435370325720
H	-5.18973791487599	-0.22152106620337	-6.67005605887198
N	2.18786188549384	6.73769332029176	-0.14629959015034
N	4.53304185031122	-2.46715672213102	-2.32646516976386
N	-4.53303828198120	2.46715805964512	-2.32646464192607
N	-2.18785928332969	-6.73769413862229	-0.14630086333906
H	2.33198938585035	5.73428719389044	-0.21741538985996
H	4.68063194932876	-2.55543716939792	-1.32232184355673
H	-2.33199349712735	-5.73428849347902	-0.21741245357652
H	-4.68062646363169	2.55543955518704	-1.32232110270394
C	-4.33426394858105	3.73097100375298	-2.98775133042907
H	-3.54442692043311	4.27763746526907	-2.46643945935203
H	-4.01918380937452	3.58064145441833	-4.02316934862912
C	3.29524261583967	7.47826335787553	0.41764981751929
H	3.70392158628639	8.17131681198965	-0.33244411888520
H	2.95527379355397	8.06921119291141	1.27622811038584
C	4.33426645388674	-3.73097032860693	-2.98774905367014
H	3.54442993066658	-4.27763577079638	-2.46643556494656
H	4.01918609078485	-3.58064501089610	-4.02316814713615
C	-3.29523924388907	-7.47826729330451	0.41765161705630
H	-3.70391871120192	-8.17132024743282	-0.33244179682549
H	-2.95526856573326	-8.06921427846427	1.27622982688806
C	-5.58793534348111	4.57962868285745	-2.97949480843431
C	-5.49956788936678	5.93599180747827	-2.69553440306967
C	-6.82963140807281	4.02634138879388	-3.26271746964418
C	-6.63383057500653	6.72881090891940	-2.70977621362336
H	-4.53668990311897	6.36067296225501	-2.45547257792794
C	-7.96496790617269	4.81782815456515	-3.27143344431870
H	-6.91442009140076	2.96800582998832	-3.46662769481094
C	-7.86880463661133	6.17246216008748	-3.00028146433244
H	-6.55661648003814	7.78368261285563	-2.48899512753479
H	-8.92665105285809	4.37664365776704	-3.49126573271969
H	-8.75495193872796	6.79036755225481	-3.00962822816809
C	4.38398776893491	6.53046696083526	0.84798988809943
C	4.99137252764349	5.70445519100680	-0.09080892842419
C	4.79481544798058	6.46589021744240	2.17137801247386
C	5.98660554237920	4.82186317719655	0.28720927456939
H	4.67883826096528	5.74731446790879	-1.12339193007986
C	5.80020053894745	5.59119064614835	2.54972168106422
H	4.32796910892855	7.09962229178342	2.91181631003137
C	6.39363581090166	4.76552020175024	1.61022630437715
H	6.43466735228171	4.17370888674924	-0.44927277582847
H	6.11765533021456	5.55289539639894	3.58214366214632
H	7.16958410351832	4.07635027964819	1.90896317726667
C	5.58793858536989	-4.57962820188058	-2.97949237057158
C	5.49957218222811	-5.93599150175403	-2.69553335126239
C	6.82963396776986	-4.02633999439664	-3.26271301722696
C	6.63383573120973	-6.72880951522229	-2.70977407154819
H	4.53669483232882	-6.36067470550620	-2.45547401996017
C	7.96497148220580	-4.81782576362657	-3.27142815264410
H	6.91442348033159	-2.96800458864357	-3.46662304464311
C	7.86880986158669	-6.17245905787401	-3.00027680179073
H	6.55662441762544	-7.78368128193421	-2.48899448706122
H	8.92665344067089	-4.37663773500557	-3.49125928616157
H	8.75495801251095	-6.79036326916611	-3.00962225851061
C	-4.38398364369163	-6.53047202185006	0.84799223755019
C	-4.99136896766614	-5.70445990217998	-0.09080731695194
C	-4.79481095704424	-6.46589328904639	2.17138146374187
C	-5.98660167949193	-4.82186658199355	0.28721080111703
H	-4.67883557073224	-5.74732008727384	-1.12339048377984
C	-5.80019624620482	-5.59119244854879	2.54972503300647
H	-4.32796445639534	-7.09962409370368	2.91182028872778
C	-6.39363257676260	-4.76552193816338	1.61022857588140
H	-6.43466295078561	-4.17371259575526	-0.44927206351419
H	-6.11765089722346	-5.55289525214984	3.58214701773476
H	-7.16958083203020	-4.07635162114314	1.90896431135991

C_4 crown-*in* in DMSO
Energy total = -359.493810787477 a.u.

Symbol	X	Y	Z
C	-3.10188840926871	0.80951889185275	2.82597550526507
C	-3.86014728563770	1.36667411716647	1.78657592567090
C	-3.53705635519201	2.61988826726344	1.26095984645127
C	-2.42055607971782	3.29633165400490	1.76801261792535
C	-1.65153354106121	2.75307759079459	2.79717654373215
C	-2.00026267625565	1.50408301154189	3.28372664071138
O	-4.93279475428401	0.64361024588392	1.35172845264808
O	-2.13755436217638	4.53363864454613	1.24128915682896
H	-1.39973873368913	1.07752956834760	4.06858628395246
C	-0.51258903064294	3.53394703742590	3.42654091536656
C	0.80963176838819	3.10187278457062	2.82604194975797
C	1.36686094073400	3.86020188859238	1.78673064965563
C	2.62015115005317	3.53720400204064	1.26123823192008
C	3.29659364292225	2.42071861011187	1.76831382508023
C	2.75324569383253	1.65159680329986	2.79735717433060
C	1.50419214737392	2.00024945425351	3.28380841923185
O	0.64378417282255	4.93282069206012	1.35183304971200
H	1.07757937289815	1.39965795916227	4.06858585811563
C	3.53405723715967	0.51255902916335	3.42661695772411
C	3.10189587933768	-0.80955518710593	2.82595261220881
C	3.86015522739929	-1.36669517087186	1.78654218492521
C	3.53706154964761	-2.61989855034255	1.26090478632758
C	2.42056325835648	-3.29635153950515	1.76794768802429
C	1.65154048323785	-2.75311239008814	2.79712241531877
C	2.00026793815315	-1.50412374714204	3.28368802816389
O	4.93279740058796	-0.64362207553037	1.35169602849261
O	2.13756453097743	-4.53364762268150	1.24119708042364
H	1.39974884763005	-1.07758584217181	4.06855972741716
C	0.51259884055023	-3.53398702947302	3.42648753964890
C	-0.80962359803305	-3.10190934398612	2.82599616612709
C	-1.36685759869275	-3.86022197120586	1.78667283504039
C	-2.62014767221299	-3.53721258608155	1.26118819802702
C	-3.29658514023850	-2.42073244914095	1.76828716411721
C	-2.75323620079174	-1.65163240941309	2.79733732487333
C	-1.50418178052155	-2.00029246128692	3.28378107195097
O	-0.64378351701465	-4.93283483851975	1.35175472831380
O	-4.53399278371117	-2.13781612056848	1.24175349139540
H	-1.07756464645106	-1.39971053534712	4.06856361079381
C	-3.53405540492504	-0.51260802340478	3.42660728150605
C	-0.56979365607098	3.45644718460789	4.96292214102579
C	3.45659146386674	0.56958404334162	4.96300038969535
H	4.59303998005747	0.64705671135439	3.18764095691671
C	0.56981171592544	-3.45651315900011	4.96286686147652
H	0.64713275018467	-4.59296104908492	3.18748901461094
C	-3.45657794067032	-0.56965244410926	4.96299480816879
H	-4.59303122971841	-0.64710918458199	3.18759430386607
H	-1.62233216478180	3.39469852547573	5.25237653448470
H	-0.07207483556649	2.56153360493322	5.33331366734754
C	0.04219753991389	4.67498477036850	5.66343456486275
H	3.39501670413524	1.62209707886005	5.25258816615083
H	2.56160128827617	0.07196805706810	5.33334412897787
C	4.67504846132948	-0.04270387259785	5.66339818998986
H	1.62235011871416	-3.39476129995380	5.25231827246518
H	0.07208777190066	-2.56160422373379	5.33326637317884
C	-0.04217695715504	-4.67506238919953	5.66336110399696
H	-3.39500771640126	-1.62217092236076	5.25256520277812
H	-2.56158962250038	-0.07204829258870	5.33335765056257
C	-4.67503004048821	0.04261773838903	5.66341511666060
H	-0.64712769201501	4.59292012453207	3.18753912236598
C	-3.31675981615450	-4.45862081273323	0.27810247627094
H	1.00111424339087	5.31124091394012	0.50471385040315
H	5.31133342363520	-1.00099083846537	0.50465403287189
H	1.19975576817364	-4.76197496940709	1.40006120413319
H	-1.00111691758051	-5.31124135520196	0.50463223523887
H	-4.76222855386922	-1.19996011434384	1.40045671112077
H	-5.31133792871956	1.00099394591808	0.50469520884348
O	4.53399200570690	2.13781457541024	1.24175372886687
C	-4.45844134261538	3.31645794250526	0.27782271564794
H	-1.19974717442929	4.76196212568972	1.40016974174094
C	3.31675674913901	4.45862569757187	0.27816042255341
H	4.76222756164367	1.19995173375687	1.40041329693133

C	-4.29081082503105	3.01767632490605	-1.20362555137777
C	-3.48457685381355	3.79387193549245	-2.01802221700032
C	-5.08581301286373	1.97684877588441	-1.74737229291567
O	-5.82109015348707	1.24596040800796	-1.05434619438847
C	3.01825903654455	4.29079789601123	-1.20332382036461
C	3.79482370795472	3.48474976403129	-2.01756246156508
C	1.97725198027623	5.08542377130268	-1.74726949329341
O	1.24606412674911	5.82057388384866	-1.05442218837872
C	4.45843928605701	-3.31644882583594	0.27774751306437
C	4.29080707609136	-3.01764286934940	-1.20369575411369
C	5.08581273037050	-1.97681114274141	-1.74742696071102
O	5.82108521815257	-1.24592824366135	-1.05438909237202
C	3.48457305100479	-3.79382825136156	-2.01810358703691
C	-3.01826592118954	-4.29077100550107	-1.20338018545738
C	-1.97726321056406	-5.08539133756116	-1.74734116353776
O	-1.24607554051168	-5.82055411308415	-1.05450713915304
C	-3.79483007563027	-3.48470637943441	-2.01760315445532
H	-3.04678590609040	-5.48838240448018	0.53566725837287
H	-4.39258058207496	-4.36280618386928	0.43919265820323
H	-5.48822219777131	3.04670842460629	0.53554775131763
H	-4.36242761736347	4.39229417837728	0.43870008878500
H	3.04678373814210	5.48838352632931	0.53574132655723
H	4.39257823357857	4.36280861478388	0.43924582248699
H	5.48822141976704	-3.04670499797740	0.53547405673468
H	4.36242529001829	-4.39228796658169	0.43860580130346
C	1.54750374284588	4.77565610139791	5.43061080345266
C	-0.25001093448767	4.60723548863389	7.16345591443972
C	-4.60737939517148	-0.24944765320045	7.16346728170844
C	-4.77544521933883	1.54791651974978	5.43042356935123
C	0.25001920318407	-4.60731718406904	7.16338413249161
C	-1.54747861001975	-4.77575114019916	5.43052070332536
C	4.60738290434752	0.24932041474638	7.16345764231656
C	4.77547482684146	-1.54799506537883	5.43036460431738
H	-0.43149398882487	5.58037619201417	5.26434882747530
H	5.58050839716129	0.43089164597381	5.26435179004928
H	0.43153092446055	-5.58044606527776	5.26427364274822
H	-5.58049260595660	-0.43095837972887	5.26434995305638
H	1.77026689105292	4.89008007119117	4.37449195495379
H	2.04563675809744	3.87666877589781	5.78736859546675
H	1.95646024345417	5.63100559384636	5.96497621648972
H	0.20055357531516	3.71635670531583	7.59683853078481
H	-1.32198900353139	4.57489206546201	7.34725800671380
H	0.15789237667168	5.47779356630522	7.67260860837691
H	-3.71629807439222	0.20078361124472	7.59677833726244
H	-4.57548959063833	-1.32141909250942	7.34738602364691
H	-5.47775123786817	0.15888628704353	7.67259215580120
H	-4.88989749130518	1.77058229012824	4.37428727325504
H	-3.87634805208255	2.04593119102878	5.78706724996001
H	-5.63068789929364	1.95708754756667	5.96479583686002
H	-0.20056342752263	-3.71644775745760	7.59676795768370
H	1.32199444525049	-4.57496040707953	7.34719793473005
H	-0.15787836415900	-5.47788421650193	7.67252628265503
H	-1.77022990467640	-4.89017761267955	4.37440022106935
H	-2.04562414374967	-3.87676843821592	5.78727331208489
H	-1.95643123014236	-5.63110418350781	5.96488311728141
H	3.71630274105753	-0.20092937586493	7.59675209507983
H	4.57548790669401	1.32128605767316	7.34740842106141
H	5.47775510815158	-0.15902474909819	7.67257348191989
H	4.88993853998134	-1.77062854595156	4.37422313944482
H	3.87637682691675	-2.04602553085018	5.78698432151288
H	5.63071451291763	-1.95717691198466	5.96473322003084
C	5.00700289741814	-1.71653841049676	-3.23859418977331
H	5.91898021296787	-1.20242677122733	-3.54798992008998
H	4.16584186962754	-1.03778574194318	-3.41004099124935
C	1.71718477071718	5.00632535559753	-3.23845751919235
H	1.20270748105249	5.91803099023047	-3.54803753881346
H	1.03884088753910	4.16483358573362	-3.40989840232350
C	-5.00700935631667	1.71660441132177	-3.23854464968663
H	-5.91898782692711	1.20250005122498	-3.54794880192414
H	-4.16585084583909	1.03785081215835	-3.41000162819636
C	-1.71720724920393	-5.00628589045194	-3.23853141272174
H	-1.20274770401590	-5.91799836944785	-3.54812168729101
H	-1.03884783072554	-4.16480614429898	-3.40996816220939
C	-2.99962434334368	-4.79862789556470	-4.03873727427414
C	-3.66382816835487	-3.52151670961160	-3.51518433844824
H	-3.06817344752604	-2.65647694297532	-3.82649727948239

H	-4.65195635737375	-3.42773504323345	-3.96854402326123
C	-4.79889510673810	2.99882204316791	-4.03895668019565
C	-3.52151268055005	3.66265025835152	-3.51557672498240
H	-2.65670370055377	3.06663913317211	-3.82684571618406
H	-3.42740324310329	4.65067817685367	-3.96908603454535
C	2.99960219894701	4.79869782355785	-4.03867110532933
C	3.66382121857077	3.52158371378170	-3.51514372734286
H	3.06817574656983	2.65654305265541	-3.82647082901346
H	4.65194960057393	3.42782054616534	-3.96850512636899
C	4.79889347764548	-2.99874611150901	-4.03902351760641
C	3.52151037404985	-3.66258382127587	-3.51565676447628
H	2.65670097079544	-3.06656906883345	-3.82691696167883
H	3.42740306123075	-4.65060343840375	-3.96918453476793
C	5.99702783844275	-3.93506044789676	-3.86317621965488
H	5.84707939352143	-4.85587893971174	-4.42292515885878
H	6.90464814724173	-3.45830100701479	-4.22619548654876
H	6.14113171386448	-4.18372929955173	-2.81449365834637
C	4.63296610861202	-2.67130104889216	-5.52563161322762
H	3.79720992742504	-1.99258841939856	-5.68295585974247
H	5.53368391073696	-2.19999801880502	-5.91145968379507
H	4.45129640948532	-3.57920893138238	-6.09657278501471
C	-3.93546972283419	-5.99711211527718	-3.86277121878308
H	-4.85640422789474	-5.84752202273984	-4.42242570929645
H	-3.45840867952245	-6.90456051218999	-4.22582499824177
H	-4.18397465344756	-6.14129055013589	-2.81405990140646
C	-2.67249390677699	-4.63251782898523	-5.52539351394082
H	-1.99413922363100	-3.79648613306818	-5.68279963499169
H	-2.20089507715543	-5.53303403293960	-5.91133065696789
H	-3.58056796260887	-4.45118809391337	-6.09617825517746
C	-5.99702764529925	3.93513628416095	-3.86309737910325
H	-5.84706998741365	4.85596752151994	-4.42282244921844
H	-6.90464663509674	3.45838874447884	-4.22613588749698
H	-6.14113966080041	4.18378105319246	-2.81441040376122
C	-4.63296650774118	2.67139820376144	-5.52556904842808
H	-3.79721018044635	1.99268768643037	-5.68290140907320
H	-5.53368351862158	2.20010052162227	-5.91140536629996
H	-4.45129549731960	3.57931419397837	-6.09649665302984
C	3.93543389964416	5.99718970500314	-3.86268799735090
H	4.85636660001905	5.84762195210298	-4.42235179981998
H	3.45835942215822	6.90463909156029	-4.22572119078593
H	4.18394381013486	6.14135110199793	-2.81397549867467
C	2.67246903370394	4.63260803194509	-5.52532929136169
H	1.99412125363148	3.79657327519482	-5.68274811064575
H	2.20086147388982	5.53312693710741	-5.91124978419302
H	3.58054342220878	4.45129670476498	-6.09611955815172
N	-2.62736924596577	4.66729414393659	-1.49246856792085
N	-4.66843291837395	-2.62780882751859	-1.49186388030691
N	2.62736246482635	-4.66725046426547	-1.49255706469510
N	4.66843339403104	2.62784758104116	-1.49183956901292
H	4.70219273333494	2.56812116750538	-0.47485576328263
H	-2.56762875702501	4.70125339698196	-0.47549686338233
H	-4.70218992202974	-2.56809744541033	-0.47487654547369
H	2.56762953741642	-4.70121955605161	-0.47558590480698
C	1.80500294355736	-5.63072829082029	-2.17426509279498
H	0.87250073930377	-5.73795130591265	-1.61295651105980
H	1.54349455235405	-5.27122205227204	-3.17445991021443
C	5.63218889357806	1.80559167614540	-2.17328878336780
H	5.73901813590341	0.87289197950725	-1.61223027457641
H	5.27323078493975	1.54445998581096	-3.17377236620414
C	-1.80498770088263	5.63073780727866	-2.17419505753827
H	-0.87254129769501	5.73805209368590	-1.61281150727084
H	-1.54336453637379	5.27113302118362	-3.17432590327248
C	-5.63219015880219	-1.80553978542456	-2.17329674938884
H	-5.73901548776941	-0.87284915403764	-1.61222309026848
H	-5.27323479020817	-1.54439271408847	-3.17377731332909
C	2.44770520932218	-6.99587206759609	-2.29781134946336
C	1.63583434908581	-8.12100806164476	-2.37668895639724
C	3.82518169942775	-7.15411981144046	-2.35452169959403
C	2.19108103800409	-9.37910197875084	-2.52392410787015
H	0.56407530448094	-8.00463508883450	-2.31116650050681
C	4.38249996076108	-8.41370725090058	-2.49784155748187
H	4.47170949489813	-6.29200655083417	-2.27959211741902
C	3.56744242885838	-9.52891274775450	-2.58626336025517
H	1.55022096042873	-10.24682069589994	-2.58308777640821
H	5.45659451382834	-8.52304940806083	-2.53945893134628
H	4.00200295736247	-10.51135067798496	-2.69838784873734

C	6.99743715357914	2.44826123319337	-2.29578648316206
C	8.12269976298617	1.63643358790424	-2.37313199641842
C	7.15564171843068	3.82572778684203	-2.35298018458648
C	9.38092759991334	2.19171030321040	-2.51915308081263
H	8.00632095110820	0.56469279666638	-2.30732267122139
C	8.41534262073579	4.38307342963993	-2.49515573104327
H	6.29338691263667	4.47221743713161	-2.27928002069868
C	9.53071436331281	3.56805353563416	-2.58187906356492
H	10.24875877866718	1.55088525801016	-2.57708012828702
H	8.52465819796571	5.45715796024873	-2.53714488097315
H	10.51325143471422	4.00263717025056	-2.69305434370882
C	-2.44771511583835	6.99584997842510	-2.29797401163114
C	-1.63584573410903	8.12096310449712	-2.37721745596080
C	-3.82519444874620	7.15409489917660	-2.35456051508302
C	-2.19109811050269	9.37901977800189	-2.52473216984289
H	-0.56407860526686	8.00459925280920	-2.31178443644607
C	-4.38251813167926	8.41365109143756	-2.49814574559239
H	-4.47172333357864	6.29200785101782	-2.27933081362881
C	-3.56746411725577	9.52882336279931	-2.58697256926709
H	-1.55023599749740	10.24671804819177	-2.58420341171479
H	-5.45661876304380	8.52299055598311	-2.53965922097003
H	-4.00202932046099	10.51123455347632	-2.69931744530971
C	-6.99743942892593	-2.44820388609446	-2.29579862637137
C	-8.12269708816265	-1.63637128102931	-2.37316397009967
C	-7.15565157230276	-3.82567400540725	-2.35297807355614
C	-9.38092817305621	-2.19164240988056	-2.51919221185033
H	-8.00631402297005	-0.56463157999929	-2.30735259541266
C	-8.41535343751565	-4.38301310617283	-2.49515723574695
H	-6.29340192017591	-4.47216377451896	-2.27927904640929
C	-9.53072288829377	-3.56798390309864	-2.58190210994285
H	-10.24875470557532	-1.55081430212286	-2.57711829578194
H	-8.52467549735902	-5.45709435726491	-2.53714563099662
H	-10.51326076588511	-4.00256429048161	-2.69307551831205

C_4 crown-*out* in DMSO

Energy total = -359.500009048132 a.u.

Symbol	X	Y	Z
C	2.86470026360134	-1.44566390538128	1.31672447738609
C	3.75747368752706	-1.49372588075468	0.24253296187585
C	4.35358781001200	-0.33354605060500	-0.26270584033267
C	4.00741945035857	0.89873127879349	0.29088279255200
C	3.07007937206745	0.97293844649364	1.32805774451935
C	2.51107981751967	-0.19769269035131	1.80383626786242
O	4.11019209194952	-2.67852219394557	-0.35617363355172
O	4.52454995252927	2.07873686169220	-0.16104930622834
H	1.78662330364009	-0.12883590820223	2.59735245956362
C	2.72544929832199	2.32392804353147	1.92507094054821
C	1.44530293189273	2.86333212292340	1.31544604722107
C	1.49288539009952	3.75394309993765	0.23943667982406
C	0.33249335171666	4.34927463719425	-0.26626626126582
C	-0.89950051377551	4.00454732372117	0.28886863725300
C	-0.97328584714914	3.06905216066590	1.32774334912053
C	0.19753964087583	2.51074358363068	1.80383776619431
O	2.67732053379970	4.10523220950920	-0.36079868015896
H	0.12903786331173	1.78778787680405	2.59874203952712
C	-2.32402345598946	2.72547905031355	1.92593028226851
C	-2.86470016890006	1.44566357791733	1.31672458016424
C	-3.75747410961480	1.49372552746674	0.24253357716580
C	-4.35358879391933	0.33354582788246	-0.26270480887745
C	-4.00742059143930	-0.89873151320054	0.29088393892171
C	-3.07007962178766	-0.97293866281089	1.32805813705609
C	-2.51107955049307	0.19769240426721	1.80383631422589
O	-4.11019187894370	2.67852171656610	-0.35617353692068
O	-4.52455121583833	-2.07873703628208	-0.16104805776624
H	-1.78662410604432	0.12883559516105	2.59735348784961
C	-2.72544965615467	-2.32392835595614	1.92507145410689
C	-1.44530334018407	-2.86333265661707	1.31544684458087
C	-1.49288577725128	-3.75394300160031	0.23943704827512
C	-0.33249365323316	-4.34927419695071	-0.26626623578834
C	0.89950018114645	-4.00454686893811	0.28886858147850
C	0.97328549053632	-3.06905242676031	1.32774388853356
C	-0.19754005263322	-2.51074444932215	1.80383894924310
O	-2.67732083913869	-4.10523207220798	-0.36079857274235

O	2.07960043702538	-4.52146566708659	-0.16300645862543
H	-0.12903824076224	-1.78778795965089	2.59874254238707
C	2.32402327774462	-2.72547938516341	1.92593028122194
C	2.71856752049340	2.27332233701806	3.46357525640042
C	-2.27231754688476	2.71830788874041	3.46438678651834
H	-3.00116831134124	3.54331107041085	1.65956731888809
C	-2.71856743630702	-2.27332319333297	3.46357576984023
H	-3.54280716934416	-3.00144490238316	1.65819355283713
C	2.27231709601568	-2.71830880258826	3.46438673999815
H	3.00116792668150	-3.54331144420950	1.65956677363353
H	3.48282877605112	1.55398357685970	3.76932471317006
H	1.76184747942821	1.92078291763147	3.84541862424308
C	3.04265055876640	3.61782657839291	4.12574772705401
H	-1.55235179333477	3.48211814523530	3.76977717128514
H	-1.92007442809463	1.76127867326282	3.84574553707531
C	-3.61615791254353	3.04295138655952	4.12762774135157
H	-3.48282857502042	-1.55398471115020	3.76932504630871
H	-1.76184742157801	-1.92078351308756	3.84541899073895
C	-3.04265075376306	-3.61782704129133	4.12574879628450
H	1.55235080131968	-3.48211884940450	3.76977748327261
H	1.92007467433171	-1.76127955779445	3.84574575900615
C	3.61615784410758	-3.04295191619582	4.12762710160876
H	3.54280668510750	3.00144467736235	1.65819268106918
C	-0.42402634134977	-5.30308771566335	-1.43771955804399
H	3.35100272327665	3.40323545170860	-0.25545717581961
H	-3.40713206160884	3.35150110834234	-0.25334857829202
H	-5.29476973254721	-1.96216858763312	-0.77252802125609
H	-3.35100333700049	-3.40323575171839	-0.25545644818639
H	1.96312729015139	-5.29034816262676	-0.77621148129473
H	3.40713262908322	-3.35150192535939	-0.25334851004683
O	-2.07960081053960	4.52146554985890	-0.16300678450566
C	5.30996887669901	-0.42569910161230	-1.43199771528404
H	5.29476858799935	1.96216857720653	-0.77252915465617
C	0.42402565719584	5.30308774572140	-1.43771989326199
H	-1.96312777887489	5.29034830697793	-0.77621154655284
C	6.78719919502852	-0.33220764801319	-1.08609516436268
C	7.51436875478053	-1.42747167526272	-0.66959443875119
C	7.40876534828088	0.93667385952631	-1.25639688489885
O	6.77308437698293	1.96061623841499	-1.57090710167615
C	0.33132407946689	6.78113577320351	-1.09512831120628
C	1.42721838376595	7.50895214536974	-0.68139765606143
C	-0.93749549030861	7.40274623054928	-1.26573658120031
O	-1.96194820535535	6.76669955167871	-1.57784877247230
C	-5.30996920331332	0.42569871658795	-1.43199718937667
C	-6.78719936465133	0.33220791576836	-1.08609485536984
C	-7.40876627037199	-0.93667303069893	-1.25639811639237
O	-6.77308572118637	-1.96061617312348	-1.57090636368380
C	-7.51436838482981	1.42747212972167	-0.66959397241504
C	-0.33132454787977	-6.78113604034089	-1.09512909417043
C	0.93749476516269	-7.40274660182963	-1.26573890685021
O	1.96194788345266	-6.76669948862039	-1.57784904620133
C	-1.42721872017064	-7.50895242838455	-0.68139777530614
H	0.39194880692907	-5.08506719878090	-2.13366225465983
H	-1.36019657623063	-5.10970264262461	-1.96503339949463
H	5.09324781831277	0.38962714394452	-2.12909828629714
H	5.11809123626563	-1.36237924090072	-1.95895780893891
H	-0.39194998999272	5.08506746960153	-2.13366219360420
H	1.36019556180793	5.10970266977470	-1.96503442874555
H	-5.09324880358352	-0.38962769597718	-2.12909784557439
H	-5.11809180518072	1.36237882639934	-1.95895737261852
C	1.93260893919842	4.64487270816228	3.91660673665925
C	3.28322707024699	3.41231201226769	5.62248839987527
C	3.40933301623228	-3.28349554607404	5.62419587314867
C	4.64378550334000	-1.93328126442422	3.91936275976375
C	-3.28322680410377	-3.41231232093530	5.62248967895518
C	-1.93260904568562	-4.64487272771991	3.91660700420364
C	-3.40933267418183	3.28349537379921	5.62419654533188
C	-4.64378512525556	1.93328052465489	3.91936305355090
H	3.96677511967278	4.00832097614379	3.68236843741097
H	-4.00664655795442	3.96721817189681	3.68455114086352
H	-3.96677552813212	-4.00832142161165	3.68236989318426
H	4.00664603476585	-3.96721869245175	3.68455005525272
H	1.76434399083698	4.82689037761338	2.85975476001595
H	1.00172036275592	4.28460733797134	4.34923180917303
H	2.19288796355803	5.58629516870230	4.39635964010530
H	2.38846505514880	3.01523012345724	6.09790346045469

H	4.10040133213992	2.71396785894331	5.79035273727509
H	3.53479018208290	4.35537409447352	6.10282317078684
H	3.01206603351781	-2.38864460446834	6.09928939783361
H	2.71065442470577	-4.10050691551459	5.79147028206375
H	4.35193171500200	-3.53528695533618	6.10532046204057
H	4.82659060059137	-1.76492118767389	2.86266236450074
H	4.28357202244233	-1.00231822091470	4.35187769212521
H	5.58477070686635	-2.19399432869318	4.39973659924375
H	-2.38846452483307	-3.01523035739421	6.09790412432005
H	-4.10040095374320	-2.71396804293035	5.79035427238505
H	-3.53478965106808	-4.35537438033349	6.10282475401959
H	-1.76434448043386	-4.82689050323015	2.85975489752841
H	-1.00172025454672	-4.28460740923114	4.34923175188852
H	-2.19288809512049	-5.58629493991963	4.39636002032807
H	-3.01206556296583	2.38864441535410	6.09928969766163
H	-2.71065397195660	4.10050672988629	5.79147103890035
H	-4.35193129091351	3.53528656418656	6.10532146939945
H	-4.82659051961380	1.76492040966113	2.86266256816150
H	-4.28357133396901	1.00231761357896	4.35187805584321
H	-5.58477001843416	2.19399378501438	4.39973707093125
C	-8.89583722874725	-1.05367323389548	-0.99128676357679
H	-9.01403538794096	-1.30804830736199	0.06710860442042
H	-9.28182993965924	-1.89331741615668	-1.57287259670505
C	-9.65536636055152	0.23287011441995	-1.29915510311483
C	-9.00298876856577	1.36020321464419	-0.49293497032247
H	-9.44110439358054	2.31239956808509	-0.79525727641965
H	-9.21908231211727	1.21202476828509	0.57085522904654
C	0.23273310957190	9.64886392913020	-1.31487239558124
C	1.36056827846537	8.99800499543495	-0.50813674094621
H	2.31263728619148	9.43511521957972	-0.81230035802548
H	1.21338723228698	9.21660893018142	0.55527984864857
C	-1.05376415950799	8.89047752120204	-1.00401136204600
H	-1.30708509535720	9.01124971821340	0.05434593965474
H	-1.89385291673903	9.27536212364731	-1.58569214508095
C	-1.36056895772292	-8.99800529394727	-0.50813753489772
H	-2.31263807419490	-9.43511518991506	-0.81230191280331
H	-1.21338853494224	-9.21660975486762	0.55527900900298
C	-0.23273287711857	-9.64886368702717	-1.31487233018282
C	1.05376419923200	-8.89047720473304	-1.00401077578565
H	1.30708407414114	-9.01124817542094	0.05434718825814
H	1.89385399034327	-9.27536194082392	-1.58569000376242
C	8.89583637926563	1.05367535553944	-0.99128731302667
H	9.01403445539861	1.30805155732602	0.06710777692699
H	9.28182821173270	1.89331955590176	-1.57287415928868
C	9.65536555315048	-0.23286851928594	-1.29915559956420
C	9.00298904552320	-1.36020214956211	-0.49293590331645
H	9.44110559811661	-2.31239871804061	-0.79525744250597
H	9.21908238966148	-1.21202274061441	0.57085384672843
C	0.54163139824213	9.57523169052397	-2.81210508550522
H	-0.26918667101477	10.01819051486090	-3.38601821348928
H	1.45786229672571	10.11518593481416	-3.03950599014107
H	0.65961084782279	8.54257885127667	-3.12990727736597
C	0.09237772751132	11.11674731152447	-0.90336903642921
H	1.01769308749681	11.65485516949020	-1.09718821260628
H	-0.70531460286901	11.59267685268171	-1.46867294573365
H	-0.14212037345461	11.20249203207166	0.15565589260421
C	9.58536294708040	-0.54312280281811	-2.79628608834412
H	10.02944715847666	0.26731249220085	-3.36987581353072
H	10.12611051127141	-1.45940161147419	-3.02160230725087
H	8.55349762948220	-0.66166733254570	-3.11644315372849
C	11.12221205828983	-0.09163486865846	-0.88426270672129
H	11.66111686809709	-1.01693947795761	-1.07590997073565
H	11.59923480848669	0.70570320550642	-1.44914768869586
H	11.20531066763970	0.14388069732769	0.17474736319976
C	-9.58536360195935	0.54312187223884	-2.79628613366825
H	-10.02944746245239	-0.26731354007226	-3.36987676817818
H	-10.12611115331300	1.45940041181713	-3.02160177188893
H	-8.55349821209347	0.66166705749992	-3.11644253300465
C	-11.12221314317829	0.09163673040411	-0.88426278694201
H	-11.66111853852260	1.01694082152370	-1.07591106947742
H	-11.59923539913968	-0.70570220577950	-1.44914712246609
H	-11.20531191216765	-0.14387826459818	0.17474732994220
C	-0.54163057282884	-9.57523159144833	-2.81210517688838
H	0.26918831473785	-10.01818976942728	-3.38601754173970
H	-1.45786088653997	-10.11518665308719	-3.03950680861777
H	-0.65961057852035	-8.54257882925155	-3.12990748944555

C	-0.09237742548648	-11.11674675951538	-0.90336860627787
H	-1.01769265274969	-11.65485459177163	-1.09718811578537
H	0.70531502890458	-11.59267625721207	-1.46867225475247
H	0.14212029435409	-11.20249156973717	0.15565642828362
N	-6.91527519579283	2.58677613653338	-0.38207337315764
N	2.58666069908677	6.91021168808088	-0.39371731177560
N	-2.58666070710726	-6.91021186741393	-0.39371674694450
N	6.91527603200315	-2.58677590479425	-0.38207450092891
H	-5.90702847220927	2.63132868473789	-0.48912730436622
H	2.63076022780543	5.90167532806097	-0.49827539343183
H	5.90702933094995	-2.63132872685215	-0.48912892280471
H	-2.63076032568169	-5.90167552566148	-0.49827491356196
C	-3.80293560996292	-7.55825473874924	0.03778078087180
H	-3.58901328279906	-8.27575234711361	0.83667744475843
H	-4.45014089683387	-6.77921882420251	0.44896871677510
C	-7.56266592086845	3.80255316557685	0.05178649755889
H	-8.27871201231485	3.58777041133341	0.85174308174615
H	-6.78296281734737	4.44943646540910	0.46221737185341
C	3.80293541936131	7.55825458881783	0.03778067054968
H	3.58901268779087	8.27575226349442	0.83667715774583
H	4.45014089253980	6.77921890579158	0.44896876085683
C	7.56266611787996	-3.80255300700353	0.05178588824817
H	8.27871089591958	-3.58777199897212	0.85174445644680
H	6.78296098846057	-4.44943509010275	0.46221473325788
C	-4.53783137665173	-8.25171461140021	-1.08953118950914
C	-5.02254752325705	-9.54436895137936	-0.93834389627100
C	-4.74443070311928	-7.58848788059702	-2.29320997185893
C	-5.69903750018961	-10.16681359557324	-1.97397961572260
H	-4.87187296744206	-10.06844931467609	-0.00467912303577
C	-5.41565360317686	-8.21180210802779	-3.32995641049005
H	-4.37718355917213	-6.58074109013901	-2.41575513692160
C	-5.89406695818524	-9.50272530400868	-3.17325856355553
H	-6.07128652312845	-11.17233928337938	-1.84472756034703
H	-5.56968148343858	-7.68750934043098	-4.26158217224380
H	-6.41806806704308	-9.98915372883591	-3.98260355382274
C	-8.25819025604830	4.53841811127149	-1.07361989676434
C	-9.55063754392846	5.02283322645049	-0.91975688664544
C	-7.59707913305797	4.74615279811851	-2.27826853102899
C	-10.17497851300641	5.70011188353671	-1.95373827861797
H	-10.07306059752249	4.87132179431830	0.01470244130804
C	-8.22229170062154	5.41815790932875	-3.31336082106616
H	-6.58951237178063	4.37910875848898	-2.40288901695631
C	-9.51301129471847	5.89624861616912	-3.15400925323478
H	-11.18030698688402	6.07215783926794	-1.82239838460190
H	-7.69964478160316	5.57302941589534	-4.24577192020313
H	-10.00092190611853	6.42082227981902	-3.96209119853409
C	4.53783142726030	8.25171441709695	-1.08953127422635
C	5.02254234294354	9.54437090453593	-0.93834600963092
C	4.74443639405657	7.58848537070836	-2.29320773637418
C	5.69903145816704	10.16681596370152	-1.97398204508873
H	4.87187020946515	10.06845041860772	-0.00468028433528
C	5.41566009451621	8.21179924447619	-3.32995389374460
H	4.37718651916272	6.58073966399275	-2.41575423141206
C	5.89406697901398	9.50272515317299	-3.17325860230947
H	6.07128383254789	11.17234025144534	-1.84472860238667
H	5.56968483337809	7.68750776137159	-4.26158090594039
H	6.41806870896045	9.98915331503276	-3.98260336151338
C	8.25819144895548	-4.53841831085080	-1.07361977297199
C	9.55063103262481	-5.02285103910095	-0.91974897962275
C	7.59708899502422	-4.74613402441311	-2.27827654963142
C	10.17496944779921	-5.70013761212531	-1.95372691636274
H	10.07306332041441	-4.87131733546756	0.01470155156258
C	8.22230523017897	-5.41813223228458	-3.31337122009030
H	6.58951320093539	-4.37911220600572	-2.40288895775420
C	9.51301351995346	-5.89624950850036	-3.15400809181708
H	11.18031050898469	-6.07215354093512	-1.82239792538037
H	7.69964611221305	-5.57303378026186	-4.24577053942203
H	10.00092517152766	-6.42082217658806	-3.96209014232416
